# Emulating evolutionary processes to morph aureothin-type modular polyketide synthases and associated oxygenases

**DOI:** 10.1038/s41467-019-11896-1

**Published:** 2019-09-02

**Authors:** Huiyun Peng, Keishi Ishida, Yuki Sugimoto, Holger Jenke-Kodama, Christian Hertweck

**Affiliations:** 1 Department of Biomolecular Chemistry, Leibniz Institute for Natural Product Research and Infection Biology (HKI), Beutenbergstrasse 11a, 07745 Jena, Germany; 2 Graduate School of Agricultural and Life Sciences, The University of Tokyo, 1-1-1 Yayoi, Bunkyo-ku, Tokyo, 113-8657 Japan; 3 Faculty of Biological Sciences, Friedrich Schiller University Jena, 07743 Jena, Germany

**Keywords:** Metabolic engineering, Natural product synthesis, Antibiotics, Antifungal agents

## Abstract

Polyketides produced by modular type I polyketide synthases (PKSs) play eminent roles in the development of medicines. Yet, the production of structural analogs by genetic engineering poses a major challenge. We report an evolution-guided morphing of modular PKSs inspired by recombination processes that lead to structural diversity in nature. By deletion and insertion of PKS modules we interconvert the assembly lines for related antibiotic and antifungal agents, aureothin (*aur*) and neoaureothin (*nor*) (aka spectinabilin), in both directions. Mutational and functional analyses of the polyketide-tailoring cytochrome P450 monooxygenases, and PKS phylogenies give contradictory clues on potential evolutionary scenarios (generalist-to-specialist enzyme evolution *vs*. most parsimonious ancestor). The KS-AT linker proves to be well suited as fusion site for both excision and insertion of modules, which supports a model for alternative module boundaries in some PKS systems. This study teaches important lessons on the evolution of PKSs, which may guide future engineering approaches.

## Abstract (web summary)

The wealth of complex polyketides is an essential source for drug discovery. Here, the authors report an evolution-guided rational morphing of modular polyketide synthases (PKSs) for aurothin and neoaurothin biosynthesis, and reveal engineering site suitable for diversifying PKS systems.

## Introduction

The wealth of complex polyketides produced by bacteria is an essential source for antifungal, antitumor, antiparasitic, and immunosuppressive agents that are currently used in the clinics^1^. Whereas their chemical synthesis is often challenging, bacteria produce these valuable compounds from simple carboxylic acid building blocks by means of modular (type I) polyketide synthases (PKSs)^2^. Each module of these highly versatile assembly lines consists of a minimal set of domains for chain elongation: a ketosynthase (KS) catalyzing Claisen condensations of the activated acyl and malonyl building blocks, an acyltransferase (AT) for selecting and loading extender units, and an acyl carrier protein (ACP) domain that serves as an anchor for the growing chain^3^. Additional β-keto processing domains for ketoreduction (KR), dehydration (DH), and enoyl reduction (ER) increase the structural diversity of the polyketide chain. The architecture of the megasynthase is typically arranged in a way that each module catalyzes a single chain elongation, ensuring that polyketide assembly progresses in a unidirectional manner until the full-length product is released by a thioesterase (TE) domain^3^. Consequently, in most bacterial type I PKSs the PKS assembly line directly corresponds to the chemical structure of the resulting polyketide.

The paradigm of successive assembly and co-linearity between assembly steps and final product sets the basis for the in silico prediction of polyketide structures from type I PKS genes, thus enabling the discovery of natural products by genome mining^4^. Likewise, the assembly line logic inspires the rational reprogramming of PKSs^2^. In many cases, such engineering attempts lead to the production of natural product derivatives^5,6^ and provide insight into the mechanisms of polyketide assembly^7^. However, routine PKS engineering is challenging not only because of constraints in DNA manipulation and recombineering^8^ but also because of the intricacies and complex dynamics of the megasynthases. For a holistic view on the molecular machineries insight into catalytic dynamics, spatial constraints, potential incompatibilities, and substrate specificities of the catalytic domains is required. It is not surprising that many combinatorial biosynthesis approaches in a mix-and-match fashion are unsatisfactory, as domains were swapped or recombined neglecting the structural impact, including non-functional regions, on protein–protein interactions. Consequently, domains and/or modules of an engineered PKS are impaired or simply non-functional^9^, which results in dramatically reduced yields or complete loss of production^10^.

Regardless of current constraints in PKS engineering, nature provides us with successful strategies to diversify polyketide structures through evolutionary processes^11^. Extensive bioinformatics analyses suggest that nature harnesses point mutations, gene duplication, gene loss, homologous recombination, and horizontal gene transfer in the evolution of type I PKSs^12–15^. However, there is little experimental evidence for such mechanisms being at work in the evolution of modular PKSs. Emulating evolutionary processes could address some of the key questions in PKS research: What are the prerequisites for the morphing of one pathway into another? Which impacts have polyketide-tailoring enzymes that coevolved with the scaffold-assembly lines? Which sites are suited for recombinations and fusions and allow the reprogramming of polyketide assembly lines?

**Fig. 1:**
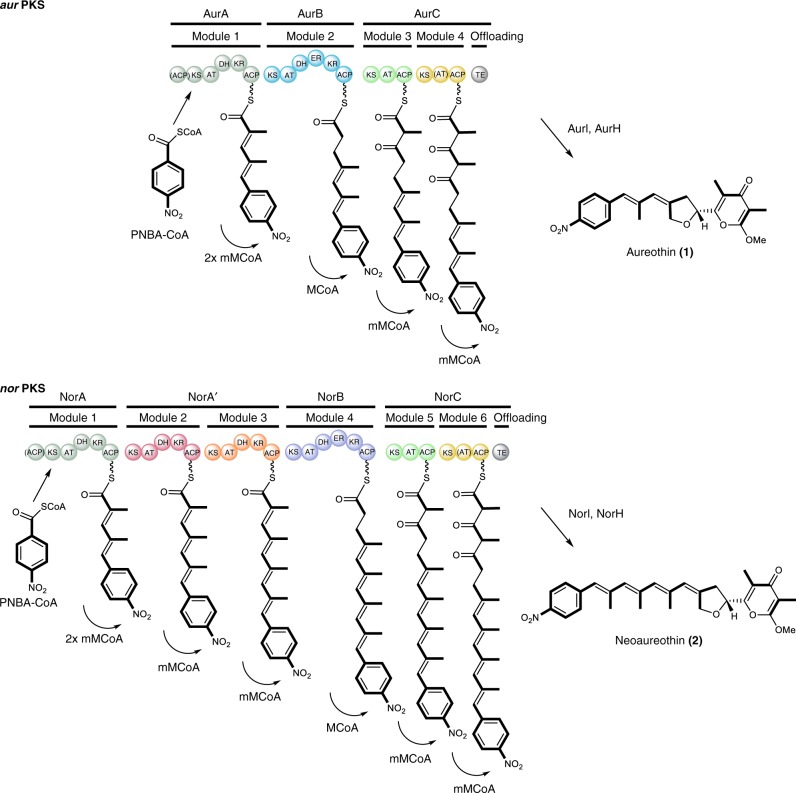
Non-colinear polyketide assembly lines for aureothin and neoaureothin biosynthesis. AurA and NorA catalyze two chain elongations. PNBA: *p*-nitrobenzoic acid, mM-CoA: methylmalonyl-CoA, M-CoA: malonyl-CoA, KS: ketosynthase, AT: acyltransferase, DH: dehydratase, ER: enoylreductase, KR: ketoreductase, ACP: acyl carrier protein, TE: thioesterase. The inactive domains in (AurA/NorA) and (AurC/NorC) are indicated with brackets. Color code in module indicates homologous proteins (except for AurB (cyan) and NorB (purple), for which different colors are used to distinguish the engineered site in Figs. 3d and 5.) These color codes correspond with those shown in Fig. 2c

We reason that similar polyketide structures and the requisite homologous biosynthesis gene clusters would be a good starting point to study the evolutionary processes in modular PKSs and answer such general questions. Owing to their compact, yet densely functionalized polyketide products, the homologous biosynthetic pathways for the antibacterial and antifungal compounds aureothin (**1**)^16^ and neoaureothin (**2**)^17^ are used as model in a proof-of-concept study. Both the *aur* PKS and *nor* PKS (Fig. 1) employ CoA-activated *p*-nitrobenzoic acid (PNBA) as starter unit^18^, and in both pathways homologous PKS modules generate polyene-pyrone backbones^19,20^, which are then subjected to further enzymatic tailoring by *O*-methyltransferases (AurI/NorI) and cytochrome P450 monooxygenases (AurH/NorH)^21^. A remarkable feature of the *aur* and *nor* PKSs is that both assembly lines contain iteratively acting domains and modules^22–24^. The only difference between the two pathways is the size of the products’ polyene backbones; compared to **1**, compound **2** is composed of two additional propionate units, which are introduced by means of two additional modules in the *nor* PKS (Fig. 1)^25^. The close relationship of the two systems suggests that one has emerged from the other. Yet, the evolutionary scenarios involved have remained a riddle.

Here we report the successful, bidirectional morphing of modular PKSs from two distinct biosynthetic pathways, employing evolutionary models deduced from in silico analyses. Surprisingly, tailoring enzymes give important clues about the direction of evolution. We also show that the KS-AT linker is a suitable engineering site, which points to alternative module boundaries in these assembly lines and may thus facilitate future engineering approaches in related PKS systems.

## Results

### Genome mining and phylogenetic analyses of gene clusters

**Fig. 2:**
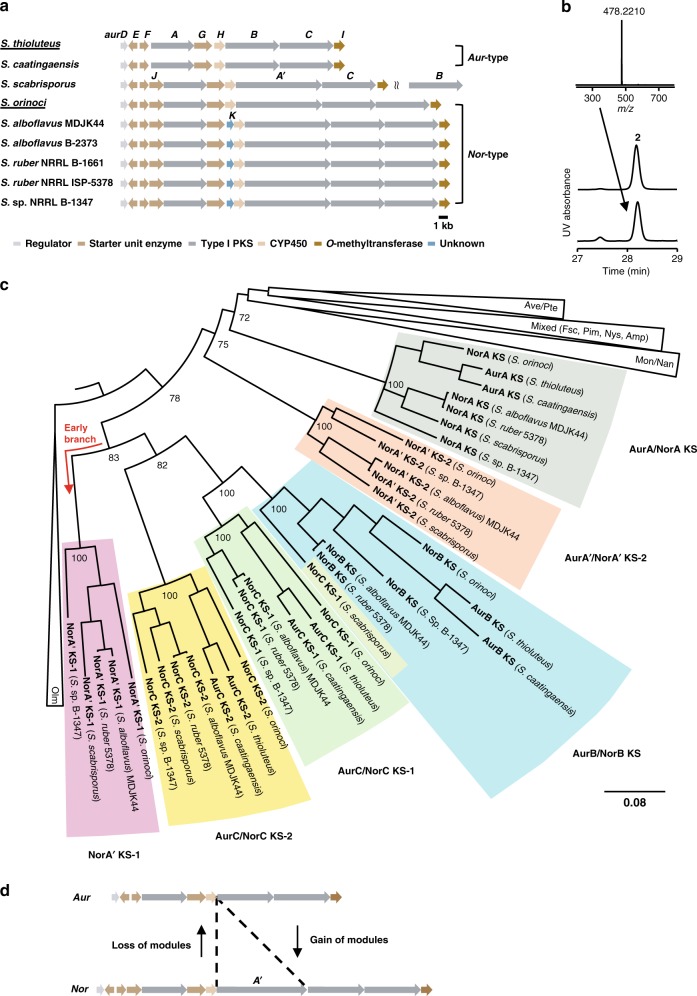
Phylogenetic analysis of the *aur* PKS and *nor* PKS. **a** The *aur*-type and *nor*-type gene clusters. The original producers of aureothin and neoaureothin are underlined. **b** The HPLC profile of authentic reference of neoaureothin (**2**, middle panel), and *S. scabrisporus* culture (bottom panel). HR-MS spectrum of neoaureothin in top panel. UV detection is at 350 nm. **c** Phylogenetic tree of KS domain amino acid sequences of the *aur*-type and *nor*-type PKSs and other actinobacterial PKSs. The tree was reconstructed by Bayesian inference. Numbers at nodes indicate clade credibility values. Branch length represents the number of amino acid changes per position. Olm, oligomycin; Tyl, tylacton; Nan, nanchangmycin; Mon, monensin; Fsc, candicidin; Pim, pimaricin; Nys, nystatin; Amp, amphotericin; Ave, avermectin; Pte, polyene macrolide from *S. avermitilis*. Color code for KS clades corresponds with those shown in Fig. 1 **d**. Possible evolutionary scenarios between *aur* PKS and *nor* PKS. The source data underlying Fig. 2c are provided as a Source Data file

To gain insight into potential gene evolution scenarios we searched for gene loci coding for assembly lines related to the *aur* and *nor* PKSs. Genome mining using BLAST (Basic Local Alignment Search Tool) in the NCBI (National Center for Biotechnology Information) database identified several gene loci with high homology to the *aur* and *nor* gene clusters (Fig. 2a). These gene clusters share the genes for PNBA starter unit biosynthesis, polyketide chain elongation, post-PKS modification and regulation (Supplementary Table 1).

Although eight of the gene clusters can be grouped either into *aur-* or *nor-*type, one orphan gene cluster from *Streptomyces scabrisporus* (DSM41855) deviates from the others as it lacks a *norB/aurB* homolog. The absence of this gene and the correct assembly of the contigs were confirmed by four pairs of PCR primers (Supplementary Fig. 1). To elucidate the product of the encoded cryptic assembly line, we cultured *S. scabrisporus* and monitored its metabolic profile. Unexpectedly, we found that this strain produces neoaureothin (Fig. 2b and Supplementary Fig. 11) despite the absence of a *nor*B homolog in the identified gene cluster (Fig. 2a). It is conceivable that a NorB homolog is encoded elsewhere in the genome. Indeed, we identified a candidate for a freestanding NorB gene (WP_078978330.1) in the yet incomplete genome sequence of *S. scabrisporus*. This finding of a split *nor* gene cluster is intriguing as it shows that gene rearrangements take place in *aur-*/*nor*-type gene clusters. Such rearrangements could in fact drive the evolution of metabolic diversity in the aureothin family.

With the enlarged set of gene clusters at hand, we aimed at gaining insight into their phylogenetic relationship, which could give clues about their evolution. Therefore, the amino acid sequences of the KS domains from those homologous gene clusters were aligned with the KS domain sequences from other actinobacterial PKS gene clusters by the GUIDANCE2 Server^26^. The aligned sequences were subjected to phylogenetic analyses, and the evolutionary tree was constructed by Bayesian inference with the MrBayes software^27^ (Fig. 2c, Supplementary Fig. 2, Supplementary Table 2). For simplification, some KS domains from *aur*- and *nor*-type gene clusters were excluded as they showed the exact same sequences.

Each module of the *aur* and *nor* clusters is monophyletic, i.e. the sequences form their own branches without intermingling of sequences from other modules. The only apparent exception is the NorC KS-1 sequence of *S. scabrisporus*, which clusters together with NorB KS sequences. An alignment of the *S. scabrisporus* NorC KS-1 with selected AurB KS/NorB KS and AurC KS-1/NorC KS-1 sequences (Supplementary Fig. 3) revealed its hybrid nature. When comparing positions that have a characteristic amino acid residue or indel pattern in the NorB KS or NorC KS-1 group, it becomes clear that the *S. scabrisporus* sequence resembles more the module B type in the amino-terminal region whereas the carboxy-terminal stretch shows higher similarity to the module C KS-1 type. This is probably due to a recombination event. Since the similarity with the module B type prevails, the sequence is located in the module B KS-1 branch of the tree.

AurA/NorA as well as NorA′-2 sequences form separate branches. The other modules, however, originated from a common ancestor. It is important to note that the NorA′-1 cluster, which comprises sequences exclusive to the *nor* cluster, forms an early branch within that big monophyletic group. Therefore, from a phylogenetic perspective, it is reasonable to propose that *aur*-type PKSs emerged from *nor*-type PKSs, possibly through gene deletion (Fig. 2d). This result is in line with the previous analysis, which suggested that a *nor*-to-*aur* PKS evolution would be the most parsimonious scenario^20^.

### Morphing the *nor* PKS into an *aur* PKS

**Fig. 3:**
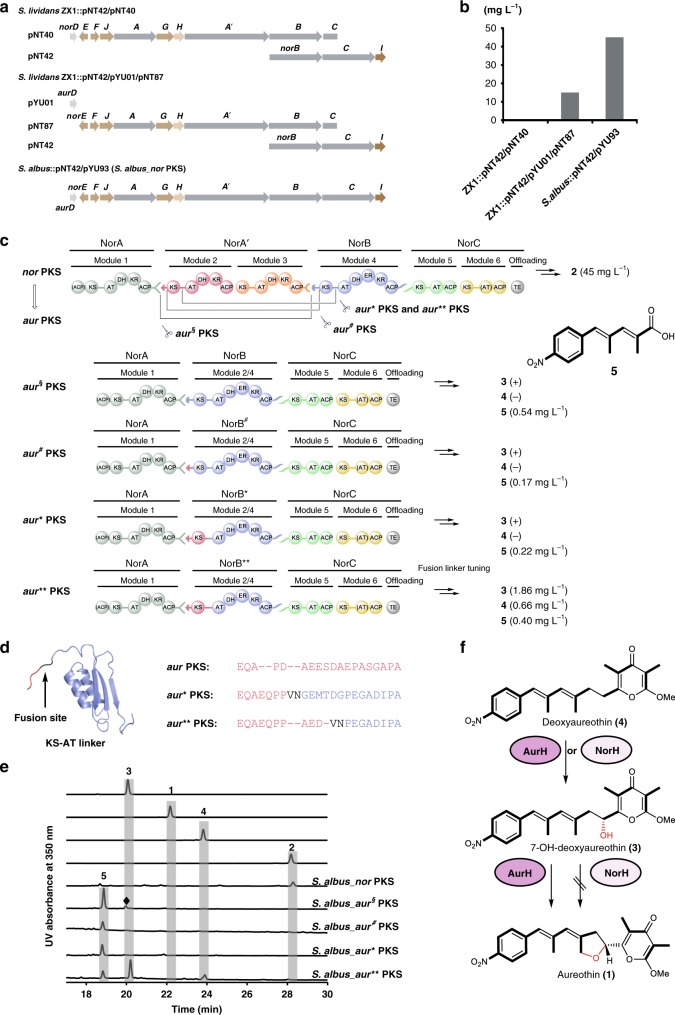
Overview of the modified *nor* PKS variants. **a** The optimization of the *nor* gene cluster heterologous expression system. The repressor gene *norD* is replaced by the activator gene *aurD*, and the intact *nor* gene cluster is assembled. **b** The titers of neoaureothin in different mutants are presented in the bar chart. **c** Domain architectures of the *aur*^§^ PKS, *aur*^*#*^ PKS, aur* PKS, and aur** PKS. Different docking domain pairs are in different shapes. The titers of polyketides in different mutants are given. Plus (+) indicates minute production only detected by LC-MS. Minus (−) indicates no production. **d** The fusion site VN is within the KS-AT linker and induced by the restriction enzyme *Hpa*I. The amino acid sequences of the KS-AT linker region among the *aur* PKS, aur* PKS and aur** PKS are aligned. **e** HPLC profile of authentic reference of 7-OH-deoxyaureothin (**3**), aureothin (**1**), deoxyaureothin (**4**), neoaureothin (**2**), *S. albus* mutant strains. The peak (marked with a diamond) with the similar retention time of **3** corresponds to a pigment produced by the host (*S. albus*). UV detection is at 350 nm. **f** NorH converts **4** to **3**. It cannot catalyze the tetrahydrofuran ring formation. The source data underlying Fig. 3b are provided as a Source Data file

For functional analyses and PKS engineering approaches, we needed to establish a robust expression system. Initially, the heterologous expression of the *nor* gene cluster was achieved in *S. lividans* by coexpression of the transcriptional regulator AurD from the *aur* gene cluster. The titer of neoaureothin was, however, unsatisfactory (15 mg L^−1^)^28^. This low yield might result from the non-concerted expression of the *nor* biosynthesis genes using a three-plasmid system. To increase neoaureothin production we optimized the heterologous expression system to reassemble the *nor* gene cluster in a continuous gene region (Fig. 3a). First, a part of the *nor* gene cluster (pNT42) was integrated into the genome of the heterologous expression host *S. albus* by site-specific recombination. The complete *nor* gene cluster was then obtained by a homologous recombination using a suicide vector (pYU93) harboring the left part of the gene cluster. The resulting strain (*S. albus*::pNT42/pYU93; *S. albus*_*nor* PKS) produced threefold higher titers (45 mg L^−1^) of neoaureothin compared to the previous construct (Fig. 3b). Thus, *S. albus*_*nor* PKS was used as a platform for PKS engineering.

To emulate the presumed evolutionary processes involved in the *nor-*to*-aur* PKS transformation (Fig. 2d) we attempted to morph the *nor* PKS into an assembly line producing aureothin. Since the polyketide backbone of aureothin lacks two methylmalonyl-derived C2 units, two requisite modules in the *nor* PKS needed to be deleted. As (AurA/NorA), (AurB/NorB), and (AurC/NorC) share high identities on the DNA and amino acid levels, we excised the gene regions for modules 2 and 3 in the *nor* gene cluster. Thereby, it was essential to consider the compatibility of the docking domains between the individual PKS proteins^29–33^, because the sequence elements at the extreme C- and N-termini of PKS subunits help mediate their interactions. The amino acid sequence alignment among PKS proteins indicated that the ^C^DD (C-terminal docking domain) of AurA/NorA and the ^N^DD (N-terminal docking domain) of AurB/NorA′ is a class 1a docking domain system (Supplementary Fig. 4)^32,33^. On the other hand, the ^C^DD of NorA′ and the ^N^DD of NorB are a class 1b system (Supplementary Fig. 4)^32,33^. In a control experiment neglecting ^C^DD/^N^DD compatibility we generated a mutant (*S. albus*_*aur*^*§*^ PKS) lacking *norA*′ (Fig. 3c). Surprisingly, this strain produced intermediate (**5**) and trace amounts of 7-hydroxydeoxyaureothin (**3**), but not aureothin (**1**) (Fig. 3e and Supplementary Fig. 12). Apparently, the different types of docking domains between NorA and NorB can communicate, albeit only weakly. To achieve higher compatibility between ^C^DD of NorA and ^N^DD of NorB, we constructed two recombinant PKS variants with different fusion sites. Initially, we have employed *S. lividans* as heterologous host^28^, but to increase neoaureothin production we reconstructed an *S. albus*_*nor* PKS expression system. Whereas the first system had a fusion site at the docking domain region (*aur*^*#*^ PKS), the second one has a fusion site at the KS-AT linker region (aur* PKS) (Fig. 3c, d and Supplementary Fig. 5). In both cases, we have swapped the ^N^DD of NorB for that of NorA′, the natural partner of the ^C^DD of NorA. In modular PKS, two hot spots for evolutionary recombination events have been suggested, KS-AT linker and post AT linker^34,35^. We initially chose one fusion site upstream of the conserved KS-AT linker, as we have already succeeded in engineering aureothin congeners using this site for recombinations (Supplementary Fig. 5)^28,36^. The constructs were introduced into *S. albus via* triparental conjugation to generate *S. albus*_*aur*^*#*^ PKS and *S. albus*_aur* PKS.

The verified recombinant strains were fermented, and the ethyl acetate extracts of the cultures were monitored by HPLC. In both recombinant strains the production of intermediate (**5**, Supplementary Figs. 19–23, Supplementary Table 8) and a trace amount of 7-hydroxydeoxyaureothin (**3**) but no aureothin (**1**) could be detected (Fig. 3e, and Supplementary Fig. 12). All recombinant strains aur^§^_PKS, aur^#^_PKS, and aur*_PKS produce elevated amounts of intermediate (**5**) compared to AurA alone^24^, indicating that the docking domains promote protein interactions. However, the low production of the full-length polyketide pointed to incorrect protein folding, likely because of suboptimal fusion sites. Thus, we revisited the fusion site in the aur* PKS and found that the recombinant KS-AT linker in the aur* PKS bore four amino acids more than that in the genuine *aur* PKS (Fig. 3d). Since this difference might have an impact on protein configuration and interactions in the aur* PKS, we shortened the recombinant KS-AT linker region by λRed-mediated recombination, yielding, in part serendipitously, the aur** PKS gene cluster (Supplementary Figs. 5 and 6).

In the extract of the culture broth of S. albus_aur** PKS, the target molecule (**1**) could still not be detected by HPLC. Instead, we noted the formation of other metabolites (Fig. 3e and Supplementary Fig. 12). Through HPLC-HRMS analysis and by comparison with an authentic reference, the compounds were identified as 7-OH-deoxyaureothin (**3**) and 7-deoxyaureothin (**4**), which differs only from **1** in that they do not form the tetrahydrofuran ring (Supplementary Fig. 12). Notably, the polyketide backbones of **1**−**3** are identical, which indicated that the *nor* PKS has been successfully morphed into an aureothin assembly line. Yet, the enzymatic tailoring of the polyketide scaffold proved to be erratic.

From in vivo and in vitro studies we know that the formation of the tetrahydrofuran ring is the last step in aureothin biosynthesis and that its installation involves two sequential C-O-bond formations catalyzed by a single cytochrome P450 monooxygenase, AurH^37,38^. Furthermore, the AurH-mediated oxygenation processes are highly fine-tuned, and changes in the enzyme or in the size of the substrate result in incomplete transformations or alternative reaction channels^39,40^. By analogy, the short aureothin backbone does not appear to be the preferred substrate of the homologous oxygenase (NorH) from the *nor* pathway. NorH is only able to convert deoxyaureothin (**4**) into the hydroxylated congener **3**, whereas the second oxidation and thus also heterocyclization do not take place (Fig. 3f). In case of the tentative *nor*-to-*aur* PKS evolution, not only the PKS needed to morph, but also the tailoring enzyme (AurH) needed to adjust.

### Mutagenesis and cross-complementation

**Fig. 4:**
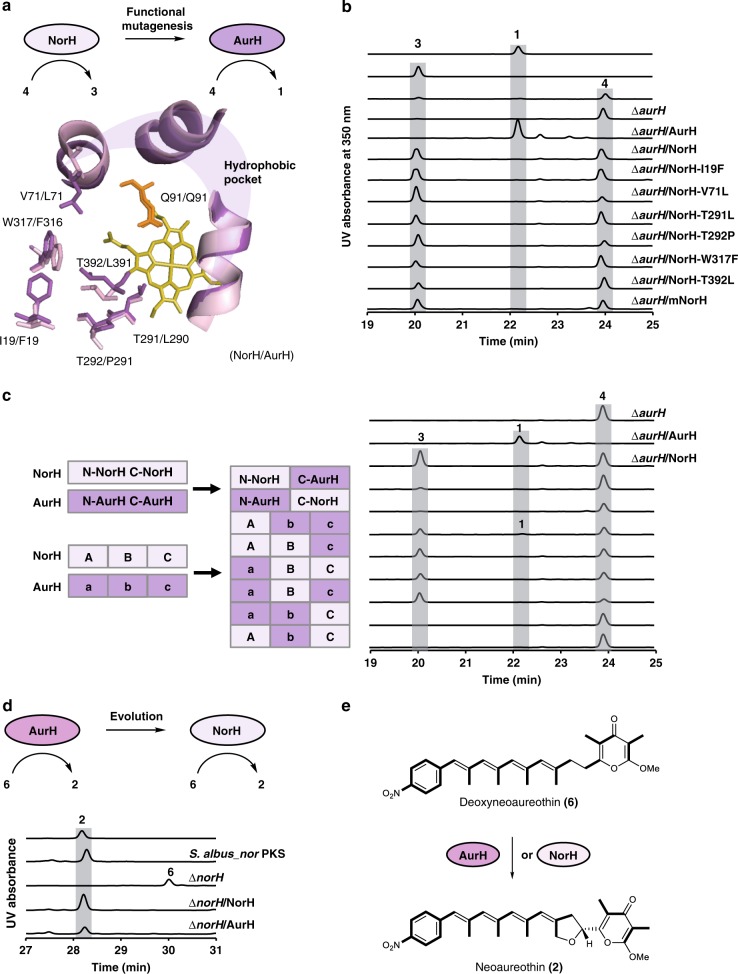
Mutagenesis and comparison of oxygenase functions. **a** Site-directed mutagenesis of NorH. The active center of NorH/AurH model shows a hydrophobic pocket. Residues that may contribute to the different sizes of the cavity are presented in stick. The catalytic residue glutamine (orange) and the cofactor heme (yellow) are indicated. **b** The HPLC profile of authentic standard of aureothin (**1**), 7-OH-deoxyaureothin (**3**), deoxyaureothin (**4**), and NorH variants. UV detection is at 350 nm. **c** Schematic presentation of strategies to prepare hybrid NorH/AurH variants, and profiling of biotransformations. The hybrid NorH/AurH variants (left panel) correspond to HPLC profiles of extracts (right panel) of those variants. UV detection at 350 nm. **d** The HPLC profile of authentic standard of **2** and *S. albus* mutant strains. **e** AurH and NorH catalyze the biotransformation from **6** to **2**

To gain insight into possible changes in the CYP450 we compared NorH and AurH. Both enzymes share similar amino acid sequences (64% identity, 75% positives), and recognize similar substrates. Thus, the overall structure of NorH is likely similar to AurH^39^. Threading of the NorH amino acid sequence onto the AurH crystal structure revealed a conserved hydrophobic pocket for binding the pyrone ring of deoxyaureothin. However, the modeling indicated that NorH has a wider cavity around the active center, as some amino acid residues possess smaller or more flexible side chains than those found in AurH (Fig. 4a). Based on this information, a number of point mutations (I19F, V71L, T291L, T292P, W317F, and T392L) could, in principle, reconfigure the active site of NorH to recognize deoxyaureothin^39^.

To test this hypothesis, we altered the active site of NorH by site-directed mutagenesis. Thus, we constructed a range of *norH* variants, including the I19F, V71L, T291L, T292P, W317F, and T392L mutants, and cloned them individually into expression vectors for complementation of an *aurH* knock-out mutant (Δ*aurH*)^37^ (Fig. 4b and Supplementary Table 3). Expression vectors containing wild-type *norH* and *aurH* served as negative and positive controls, respectively. All plasmids were introduced into the ∆*aurH* mutant by triparental conjugation, and the metabolic profiles of the individual transformants were monitored by HPLC-MS (Fig. 4b and Supplementary Fig. 13).

The ∆*aurH* mutant produces exclusively **4**; when complementing the mutant with native AurH, **4** is readily converted into **1** (positive control). In contrast, the mutant complemented with NorH partly transformed **4** into **3**, and it was not capable of forming **1** (negative control) All ∆*aurH* mutant strains complemented with point-mutated NorH variants showed the same chemotype. The only difference was a slightly increased **3**-to -**4** ratio for NorH-V71L and NorH-T292P (Fig. 4b and Supplementary Fig. 13). These results indicate that these individual point mutations of NorH are not sufficient to reconfigure its active site to generate the THF ring of aureothin.

Therefore, we generated an expression plasmid for a NorH variant containing all six point mutations (I19F-V71L-T291L-T292P-W317F-T392L). Yet, in the metabolic profile of the ∆*aurH* strain complemented with the multiple point-mutated NorH variant, **1** could not be detected, either (Fig. 4b and Supplementary Fig. 13). Thus, we scrutinized the highly similar P450 monooxygenases NorH and AurH (Supplementary Fig. 7 and Supplementary Table 4) and attempted to target the protein domains that are relevant for THF-ring formation by constructing chimeric NorH variants. AurH variants adopt different conformations mainly at the B2 and B2′ two-helix-bundle, FG-loop and β2-loop that surround the active center and approach the center after binding to substrate^39^. These residues likely generate steric pressure to bend the intermediate and push it towards the reaction center, facilitating THF-ring formation. In order to test whether these residues are important for THF-ring formation, five gene regions around these residues from AurH were amplified and used to replace each corresponding region in the NorH gene (Supplementary Fig. 8, Supplementary Table 5). We noted, however, that these chimeras also produce exclusively 7-OH deoxyaureothin (**3**) (Supplementary Figs. 14 and 15). We also created AurH/NorH hybrids differing at the N-terminal end of the α helix (Supplementary Fig. 8, Supplementary Table 6). These head/tail exchange hybrids showed reduced catalytic activity (Fig. 4c and Supplementary Fig. 16). Thus, the exchanged region was narrowed down to avoid possible deleterious effects on the overall structure. The fusion sites were placed within the C helix and the K helix (Supplementary Fig. 8). Thus, NorH was divided into three areas, part A, B, and C. Correspondingly, AurH was dissected into parts a, b, and c (Fig. 4c). The HPLC profiles of the obtained hybrids, NorH/AurH ABc, AbC, aBC, Abc, aBc, and abC variants, indicated that only hybrid Abc variant could transform 7-deoxyaureothin (**4**) to aureothin (**1**), albeit only incompletely (Fig. 4c, Supplementary Fig. 16).

Taken together, structure-based, rational mutations and domain swapping of NorH are not sufficient to reconfigure the active site of NorH to function like AurH. The 70% C-terminal AurH hybrid NorH (Abc) shows only substantially reduced activity. These results indicate that complex evolutionary processes would be required to maintain THF ring formation activity when mutating NorH to AurH. Consequently, we also considered the reverse scenario and aimed at emulating a potential *aur*-to-*nor* PKS evolution.

Therefore, we interrogated the substrate specificity of AurH with respect to the enzyme’s ability to transform deoxyneoaureothin (**6**) into **2**. To this end, *norH* was deleted in *S. albus*_*nor* PKS using the λRed system. The resulting ∆*norH* mutant was fermented, and the culture extract was analyzed by HPLC. The HPLC profile showed that ∆*norH* lost the ability to produce **2**. Instead, a different metabolite was detected (Fig. 4d and Supplementary Fig. 17). The structure of this metabolite was determined as **6** by ^1^H and ^13^C NMR, ^1^H-^1^H COSY, HSQC and HMBC (Supplementary Figs. 17 and 24–29, Supplementary Table 9).

The NorH and AurH expression plasmids were introduced into the ∆*norH* mutant by triparental conjugation. As expected, NorH restored the production of **2** (Fig. 4d and Supplementary Fig. 17). Surprisingly, complementing the ∆*norH* mutant with AurH also restored the production of **2** (Fig. 4d and Supplementary Fig. 17). This result demonstrates that AurH is relatively flexible in substrate specificity and highly efficient in converting **6** to **2** (Fig. 4e and Supplementary Table 7). The broader substrate specificity of AurH indicates that an evolution from AurH to NorH is a more probable scenario according to the generalist-to-specialist model in enzyme evolution, where ancestral enzymes show higher promiscuity and the more specialized enzymes are evolved to catalyze specific reactions^41,42^.

### Morphing the *aur* PKS into a *nor* PKS

**Fig. 5:**
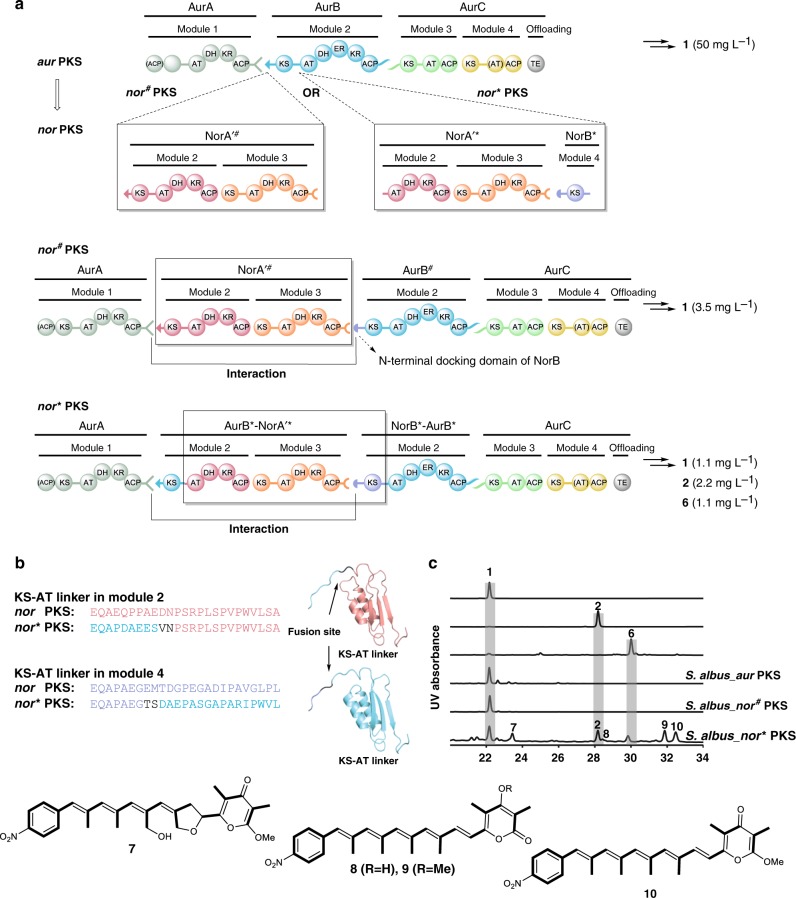
Overview and metabolic profile of modified *aur* PKS variants *nor*^*#*^ PKS and nor* PKS. **a** Domain architectures of the *nor*^*#*^ PKS and nor* PKS. The N-terminal docking domain of *aurB* is swapped to that of *norB* in the *nor*^*#*^ PKS. In the nor* PKS, the length of recombinant KS-AT linker is manipulated to match that of the *nor* PKS. **b** The fusion sites are within the KS-AT linker and induced by the restriction enzyme *Hpa*I and *Spe*I. The amino acid sequences at the fusion regions of *nor* PKS and nor* PKS are aligned. **c** HPLC profile of authentic standard of aureothin (**1**), neoaureothin (**2**), deoxyneoaureothin (**6**), and *S. albus* mutant strains. UV detection is at 350 nm

Since AurH catalyzes the transformation of **6** into **2**, we investigated the possibility of an *aur*-to-*nor* gene cluster evolution. Therefore, we aimed at integrating modules 2 and 3 of the *nor* PKS between modules 1 and 2 of the *aur* PKS. To achieve this goal, two chimeras with different recombination sites were constructed: one fusion site is in the docking domain region (*nor*^*#*^ PKS), and the other one in the KS-AT linker region (nor* PKS) (Fig. 5a).

For the first construct (*nor*^*#*^ PKS), we generated a fusion site at the N-terminal docking domain region of *aurB* and PCR-amplified intact *norA*′ including its N- and C-terminal docking domains (*norA*′^#^) (Supplementary Fig. 9). The alignment of amino acid sequences of the docking domains showed that the interaction between AurA/AurB and NorA/NorA′ are of class 1a, and the interaction between NorA′/NorB is of class 1b (Supplementary Fig. 4)^29,30^. To facilitate the interaction between NorA′ and AurB, the N-terminal docking domain of AurB was swapped with that of NorB to generate the *nor*^*#*^ PKS variant. For the second construct (nor* PKS), we chose a fusion site within the KS-AT linker region of AurB, and amplified the gene fragments for the region between the NorA′-AT2 domain and the NorB-KS4 domain (NorA′*-NorB*) for recombination (Supplementary Figs. 5 and 10). To maintain the overall conformation of proteins in the nor* PKS, the length of the recombinant KS-AT linker was adjusted to match the size of the genuine *nor* PKS (Fig. 5b and Supplementary Fig. 5).

The verified constructs were introduced into *S. albus* to generate *S. albus_nor*^*#*^ PKS and S. albus_nor* PKS. In the metabolic profile of *S. albus_nor*^*#*^ PKS compound **2** could not be detected. Yet, PNBA and **1** were produced, indicating that gene expression and polyketide production were functional (Fig. 5c). In the case of *S. albus*_nor* PKS, we detected **1** and **2** (Fig. 5c and Supplementary Fig. 18), which indicated that we successfully modified the *aur* PKS to produce the homologous polyketide **2**. Thus, we showed that it is possible to modify the *aur* gene cluster to the *nor* gene cluster through PKS engineering in a manner that emulates natural evolutionary processes. The strain expressing the nor* PKS genes also produces a series of congeners, non-oxidized 7-deoxyneoaureothin (**6**), over-oxidized 11a-hydroxyneoaureothin (**7**), non-reduced 7-deoxy-7-dehydroneoaureothin (**10**), non-reduced 2-pyrone-7-deoxy-7-dehydronoeaoreothin (**9**), and 2-pyrone-4-desmethyl-7-deoxy-7-dehydronoeaoreothin (**8**). The structures of all compounds were elucidated by NMR analyses (**7**, Supplementary Figs. 30–34, Supplementary Tables 10 and 8, Supplementary Figs. 35–40, Supplementary Tables 11 and 9, Supplementary Figs. 41–46, Supplementary Tables 12 and 10, Supplementary Figs. 47–52, Supplementary Table 13). The presence of three highly instable, non-reduced congeners indicates that the enoylreductase domain of AurB cannot process the polyketide intermediate accurately, likely because the length of the intermediate is different to the original substrate. This observation is in line with the remarkable finding that the nor* PKS can still produce **1**. A plausible explanation could be that two modules are skipped during polyketide chain elongation. The phenomenon of PKS module skipping has previously been reported in a PKS engineering study where module 2 from the rapamycin PKS was inserted between module 1 and 2 in DEBS1-TE^43^. In the hybrid PKS the polyketide chain underwent direct ACP-to-ACP transfer to pass through rapamycin module 2^44^. In the case of nor* PKS, the formation of the shorter chain may be rationalized by a similar ACP-to-ACP transfer or by the interaction between the C-terminal docking domain of AurA and the N-terminal docking domain of NorB, which is also present in the *nor*^*#*^ PKS.

To corroborate this model, we generated another strain (*S. albus*::pHY129) in which the N-terminal docking domain of AurB was swapped to that of NorB without inserting NorA′^#^ (Supplementary Fig. 9). By LC-MS monitoring we found that the recombinant strain produces aureothin. This experiment confirms that the C-terminal docking domain from AurA in fact recognizes the N-terminal docking domain from NorB. Thus, the shortcut in the nor* PKS can be rationalized. It remains unclear why the class 1a C-terminal docking domain of AurA cannot interact with the class 1a N-terminal docking domain of NorA′ in *S. albus*_nor#PKS. Weak interactions between different docking domain types have also been observed in other PKS systems^45^.

## Discussion

With the aim of rationally designing therapeutics by synthetic biology approaches^46^, a giant body of knowledge on modular polyketide assembly lines has been built in the past three decades. Inspired by pioneering works on the erythromycin PKS, many natural product derivatives have been created by mutations or replacements of PKS domains and swaps of entire PKS modules or subunits^7^. Yet, information on the structures and dynamics of modular PKSs has been limited^47^, and many trial-and-error engineering approaches gave unsatisfactory results because of incompatibilities, non-functional constructs and low yields. Only recently, cryo-electron microscopy provided the first complete type I PKS module structure, which may grant insights that could facilitate rational PKS engineering^47–49^. Complementary to structure-guided engineering approaches, studying nature’s strategies to evolve structural diversity may give important clues for synthetic biology. In silico analyses of PKS domains, modules, and gene clusters have suggested the impressive wealth of polyketide structures has evolved through point mutations^50^, gene duplications^51^, homologous recombinations^52^, and horizontal gene transfer^53^. An evolution-guided reprograming of PKSs appears to be a promising strategy to generate desired polyketide metabolites^54–56^.

In this study we used the related *aur* and *nor* gene clusters as model system to study and emulate potential evolutionary processes that lead to the structural diversity of complex polyketides. Through genome mining we identified nine homologous *aur*- and *nor*-type gene clusters, including one split *nor*-type gene cluster that already hinted to gene rearrangement events implicated in pathway evolution. The phylogenetic analyses of the *aur*- and *nor*-type PKSs predicted that the *aur* and *nor* gene clusters have a common evolutionary ancestor, and that the *nor* gene cluster likely represents the older type. Thus, we established a robust expression system for the expression of the *nor* biosynthetic gene cluster and emulated the possible natural *nor-*to-*aur* PKS evolution. By deleting two modules in the *nor* PKS we succeeded in the PKS morphing to generate the aureothin backbone. Yet, we unexpectedly found that the narrow substrate tolerance of the polyketide-tailoring enzyme NorH hampers *O*-heterocyclization, the final biosynthetic step towards aureothin. Notably, multiple point mutations and enzyme domain swapping are not sufficient to adjust the enzyme function. Eventually, we succeeded to create a chimera that partially converts deoxyaureothin (**4**) into aureothin (**1**). The homolog AurH, however, was found to have broader substrate specificity than NorH and readily transforms the deoxy precursor of neoaureothin into the final product.

**Fig. 6:**
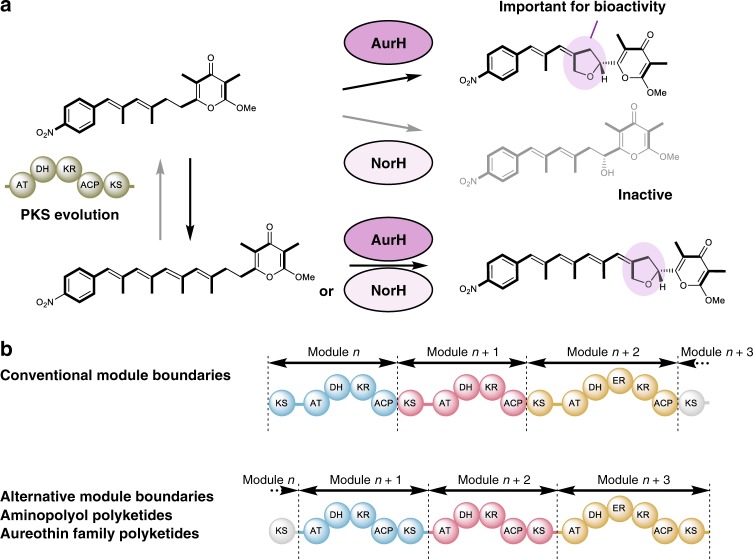
Model of plausible evolutionary processes in the *aur/nor* modular type I PKSs and tailoring enzymes. **a** Insertion of two additional modules yields a functional *nor* assembly line in which bioactivity is maintained. **b** Scheme showing paradigm shift related to conventional and alternative PKS module boundaries that are in line with the proposed module insertions into the KS-AT linker regions

According to the model of generalist-to-specialist enzyme evolution^41,57^, the substrate specificities of the oxygenases may indicate that AurH is more ancient than the more specialized NorH (Fig. 6a). Furthermore, since the presence of the tetrahydrofuran ring is crucial for the antifungal activity of aureothin^58^, the intermediate stage of an *aur* PKS and an oxygenase yielding the less active 7-OH-deoxyaureothin would not be preferred from a functionalistic point of view^12^. Presuming that the post-PKS gene coevolved with the PKS genes in the same gene locus, the *aur* gene cluster would represent the ancestor. The successful reverse (*aur*-to-*nor*) PKS morphing by insertion of two modules corroborated the revised model, although it contrasts with the conclusion drawn from the phylogeny. This is a rare example of successfully engineering two additional modules into a PKS and shows that the *aur* and *nor* gene cluster share the same evolutionary origin.

The general lessons learned from the *aur*/*nor* engineering experiments are that (1) evolutionary models guide the morphing of modular PKSs from two distinct biosynthetic pathways into another, and that (2) the specificity of tailoring enzymes may give additional clues on the direction of evolution. Phylogenies alone should be interpreted with care. The third important lesson relates to the design of functional PKS constructs; (3) the KS-AT linker is particularly well suited as engineering site for both excision and insertion of modules. As in a plethora of other, mainly unpublished, studies, engineering the *aur* and *nor* PKSs has been a tour de force because of suboptimal recombination sites that led to less active or fully non-functional constructs. In our efforts to alter the *aur* and *nor* PKSs we found that best results for excising or inserting modules were obtained when the recombination sites were located in the KS-AT linker region. Only in this way it has been possible to create a nor* PKS that produces the aureothin backbone, and an aur** PKS assembling the neoaureothin scaffold. Still, the product yields of the engineered aur**_PKS (aureothin backbone) and the nor*_PKS (neoaureothin backbone) are substantially lower (10–20-fold) than those of the corresponding native assembly lines, and could be optimized by further adjusting the KS-AT linker region^59^, for example. It should be pointed out that the constructs with recombination sites within the docking domains (*aur*^#^ PKS and *nor*^*#*^ PKS) were non-functional.

Our results of the engineering experiments, specifically the location of the fusion sites, are particularly intriguing in light of a recently proposed revision of the PKS module boundaries. Contrary to the textbook architecture of modules (KS-AT-X-ACP), in the phylogeny-based model a PKS module would start with an AT domain and end with the KS domain that was previously assigned to the downstream module (AT-X-ACP-KS)^52,60^. Notably, in the alternative model the boundary for each module is the KS-AT linker, which in our case proved to be the best site for adding or deleting modules (Fig. 6b). Therefore, our results strongly support this alternative PKS module definition, and suggest that the KS-AT linker is an alternative construction site for future PKS engineering. Although *trans*-AT PKSs^61^ likely evolved in a fundamentally different fashion from *cis*-AT systems like the ones investigated here, it should be noted that their mosaic structure^14^, the KS substrate specificities^62^, and ACP-KS relationship^63^ are in accord with the revised module boundaries. It is remarkable that this scheme extends to the related modular non-ribosomal peptide synthetases (NRPSs). Recently, a platform has been established that allows a swift recombination of NRPS modules to create artificial peptides^64,65^. Interestingly, for the de novo design and engineering of NRPSs this strategy employs the C-A (condensation-adenylation) linker region as fusion point^66^, which corresponds to the KS-AT linker in PKSs.

In conclusion, our work is a proof-of-concept for a successful evolution-guided reprogramming of modular PKSs. Beyond insight into nature’s chemical diversification by evolving PKS and post-PKS enzymes, the knowledge gained supports a paradigm for module boundaries for at least some assembly lines, which may advance the field of rational PKS engineering and synthetic biology.

## Methods

### Strains and media

For routine subcloning, *E. coli* strains TOP10 (Invitrogen) and XL1-Blue (Agilent) were used, *E. coli* HB101/pRK2013 for triparental conjugation^67^, and BW25113/pIJ790 for PCR-targeting procedures^68^. For plasmid selection, *E. coli* strains were cultured in Luria-Bertani (LB) medium supplemented with spectinomycin (100 µg mL^−1^, Sigma-Aldrich), apramycin (50 µg mL^−1^, Sigma-Aldrich), kanamycin (25 µg mL^−1^, Sigma-Aldrich) or chloramphenicol (25 µg mL^−1^, Carl Roth). *S. albus* (kindly provided by Prof. Dr. Jose A. Salas, University of Oviedo, Spain) was used as host strain for heterologous expression experiments. For metabolite production, *Streptomyces* strains were grown at 28 or 30 °C with 150 or 180 rpm orbital shaking in TSB, R5 (103 g L^−1^ sucrose, 10 g L^−1^ glucose, 5 g L^−1^ yeast extract, 5.73 g L^−1^ TES, 1 g L^−1^ casamino acids, 0.05 g L^−1^ KH_2_PO_4_, 0.25 g L^−1^ K_2_SO_4_, 10.12 g L^−1^ MgCl_2_·6H_2_O, 2.94 g L^−1^ CaCl_2_·2H_2_O, 0.28 g L^−1^ NaOH, 1.5 ml 20% l-proline, 2 mL trace element solution) or J-medium (100 g L^−1^ sucrose, 30 g L^−1^ tryptic soy broth, 10 g L^−1^ yeast extract, 4.7 g L^−1^ MgCl_2_) for 2 days. Seed cultures (10 mL) were used to inoculate 100 mL of the up-scaled culture in R5, modified R5 (R5 medium without sucrose and 1 g L^−1^ casamino acids) or M10 medium (10 g L^−1^ malt extract, 4 g L^−1^ yeast extract, 4 g L^−1^ glucose, pH 7.3). The fermentation was stopped after 5 days cultivation (orbital shaking at 28 °C). For sporulation, *Streptomyces* strains were grown on MS (mannitol soya flour) agar plates for 7 days. For conjugation MS agar plates supplemented with MgCl_2_ (10 mM) were used, which were overlaid after one-day incubaction with nalidixic acid (20 µg mL^−1^, Carl Roth) and appropriate antibiotics.

### General DNA procedures

All routine experiments such as PCR, DNA isolation, plasmid preparation, restriction digests, gel electrophoresis, ligation, and transformation were performed according to standardized methods for *E. coli*^69^. Restriction enzyme digested and PCR-amplified DNA fragments were purified from agarose gel using the Monarch DNA Gel Extraction Kit (New England Biolabs).

### Phylogenetic tree construction

The amino acid sequences of the KS domains of the aureothin and neoaureothin gene clusters and other selected actinobacterial PKS clusters (oligomycin, olm; tylacton, tyl; nanchangmycin, nan; monensin, mon; candicidin, fsc; pimaricin, pim; nystatin, nys; amphotericin, amp; polyene macrolide from *Streptomyces avermitilis*, pte; avermectin, ave) were aligned using the GUIDANCE2 Server^26^. Alignment columns with a score below 0.93 were removed from the alignment. Tree reconstruction was done by the method of Bayesian inference using version 3.2.6 of the MrBayes software^27^. The calculation used the model jumping option and was run with four independent chains for 1.5 million generations sampling one tree per 1000 generations. Trees were summarized with a burnin of 300 trees.

### Construction of *S. albus*_*nor* PKS

The 0.8 kb *Bsa*AI-*Sap*I DNA fragment from pBluescript II SK (+) (Stratagene) was blunted and ligated into *Afe*I-digested pYU01^28^, in which *aurD* was cloned downstream of constitutive promoter, *actII*-ORF4^70^, to yield the plasmid pYU91. The 34.5 kb *Xba*I DNA fragment from pNT87^28^ was cloned into *Spe*I-digested pYU91. The resulting suicide vector pYU93 was introduced into *S. albus*::pNT42 containing *norB*, *C*, and *I* by triparental conjugation to give the recombinant strain *S. albus*::pNT42/pYU93 (*S. albus_nor* PKS).

### Construction of *S. albus_aur*^*§*^ PKS

To remove the *norA*′ gene, primer pair NorA′-HpaI-fw (5′-CCGGACGTCCTGCCACTGCGCTTCGGCGCCTGAGCGAGCGTTAACGAACACGTAGAAAGCCAGTC-3′) and NorA′-HpaI-rv (5′-GCAGCCCTTCGGCGTCCAGTCGGTCGATGGAGCCGGGTTAACTTGGTCGGTCATTTCGAACC-3′) were used to amplify the kanamycin resistance cassette from pK19^71^. The obtained amplicon was introduced into *E. coli* BW25113/pIJ790 harboring pYU93. The targeted *norA*′ gene was replaced by the kanamycin resistance cassette through the λRED system to generate pYU93 ΔnorA′-Kan. This plasmid was restricted by *Hpa*I and then self-ligated to generate pYU93 ΔnorA′. This plasmid was introduced via triparental mating into *S. albus*::pNT42 to generate *S. albus*::pNT42/pYU93 Δ*norA*′ (*S. albus_aur*^§^ PKS).

### Construction of S. albus_aur* PKS and *S. albus_ aur*^*#*^ PKS

The 23.7-kb *Xba*I DNA fragments from pYU48^28^ and pYU69^28^ were individually ligated into *Spe*I-digested pYU91. The resulting suicide vectors pYU95 and pYU98 were introduced into *S. albus*::pNT42 to generate *S. albus*::pNT42/pYU95 (S. albus_aur* PKS) and *S. albus*::pNT42/pYU98 (*S. albus_aur*^*#*^ PKS), respectively.

### Construction of S. albus_aur** PKS

The S. albus_aur** PKS mutant was constructed by a PCR-targeting approach. To adjust the length of the KS-AT linker region in norB**, primer pair KS2_fw_HpaI_2 (5′-GTCATCCTGGAACAGGCCGAGCAGCCCCCGGCCGAGGACGTTAACGGAACTTCGAAGTTCCCGCC-3′) and KS4_rv_HpaI_2 (5′-CAGGGGCAGGCCCACGGCCGGGATGTCCGCGCCCTCCGGGTTAACGGAATAGGAACTTCATGAGC-3′) were designed and used to amplify the spectinomycin resistance cassette *aadA* from pIJ778. The 1.4-kb PCR product was introduced into *E. coli* BW25113/pIJ790 harboring pNT87. Mediated by the λRED system, the targeted DNA region was replaced by the *aadA* cassette to generate pYU71. pYU71 was digested with *Hpa*I to remove the *aadA* cassette and then self-ligated to yield pYU72. The 23.6 kb *Xba*I DNA fragment was cut out from pYU72 and cloned into *Spe*I-digested pYU91 to obtain suicide plasmid pYU99. Finally, pYU99 was introduced into *S. albus*::pNT42 to generate *S. albus*::pNT42/pYU99 (S.albus_aur** PKS).

### Site-directed mutagenesis of NorH

The Zero Blunt PCR Cloning Kit (Invitrogen) was used for *norH* subcloning. The QuikChange II XL Site-Directed Mutagenesis Kit (Agilent Technologies) was used for site-directed mutagenesis. The *E. coli*-*Streptomyces* shuttle vector pMR17^38^ was used for *norH* expression in *S. albus*::pHJ68 (∆*aurH*)^37^ and as the template for site-directed mutagenesis. The primers for site-directed mutagenesis are listed in Supplementary Table 3.

### Chimeric NorH variants with domain swaps

Five pairs of partially overlapping primers (Supplementary Table 5.) were designed using the same strategy as for site-directed mutagenesis, except that the overlapping regions were the corresponding sequences from AurH.

### Hybrid NorH/AurH variants

Two hybrid NorH/AurH variants were constructed with a fusion site (*Fsp*I in the corresponding genes) located at the N-terminal end of I helix (Supplementary Fig. 8). The N-terminal region of *norH* was amplified with primer pair N-NorH-fw/rv, while the C-terminal region of *aurH* was amplified with C-AurH-fw/rv. Then, the two fragments were assembled with pMR17/*Spe*I + *Ssp*I using the NEBuilder^®^ HiFi DNA Assembly Cloning Kit to generate the N-NorH-C-AurH hybrid. The same procedure was used to generate the N-AurH-C-NorH hybrid. Primer pairs N-AurH-fw/rv and C-NorH-fw/rv-1 were employed to amplify the N-terminal region of *aurH* and C-terminal region of *norH*, respectively. The primers used in these hybrid preparations are listed in Supplementary Table 6.

### Construction of Δ*norH* mutant

The PCR-targeting approach was used to replace the gene *norH* in pYU93 with the kanamycin resistance cassette. The kanamycin resistance cassette was amplified from pCR-Blunt vector (Invitrogen) with primers Kan-NorH-fw-1 (5′-GCGGCAGCAGGCCGCACCCTTGAGCGAAGGACCGTGTTACTGGGCTATCTGGACAAG-3′) and Kan-NorH-rv-1 (5′-GCCCTTCAACCGGAGTTGAGATATCCGATGGCTCGCCCTGATGCGGTGTGAAATAC-3′). The PCR product was introduced into *E. coli* BW25113/pIJ790 harboring pYU93 to generate recombinant and suicide plasmid pHY42. pHY42 was introduced into *S. albus*::pNT42 to yield *norH* knock-out mutant *S. albus*::pNT42/pHY42 (Δ*norH*).

### Construction of *S. albus_nor*^*#*^ PKS

*E. coli*‐*Streptomyces* shuttle plasmid pHJ48^19^ containing the *aur* gene cluster was digested with *Kpn*I. The 15 kb DNA fragment was inserted into the pCR-Blunt vector to yield pHY115, which was used for further gene cloning steps. The PCR-targeting approach was used to insert the spectinomycin resistance cassette *aadA* into the point mutation site. The *aadA* cassette was amplified from pIJ778 with primer pair Spec-norA′-fw (5′-GGGCCGGACGCCCTGCCGCTGCGCTTCGGCGCGGCCTGAAATATTATTCCGGGGATCCGTCGACC-3′) and Spec-norA′-rv (5′-GGGGTAGCGACATCCCATTGACACCACCGCGACGGGCTCCTTAAGTGTAGGCTGGAGCTGCTTC-3′), which also introduced *Ssp*I and *Afl*II restriction sites into the amplicon. The 1.5 kb *aadA* cassette was introduced into *E. coli* BW25113/pIJ790 harboring pHY115. Then, this cassette was inserted into pHY115 by homologous recombination, yielding pHY119. The 16 kb *Kpn*I-digested pHY119 fragment was inserted into *Kpn*I-digested pHJ48-oriT fragment to obtain pHY124. The gene region for the N-terminal docking domain region of AurB was replaced by that of NorB in pHY124 to generate pHY129. A DNA fragment *norA*′^*#*^ including the gene regions for the N- and C-terminal docking domains was constructed as follows: The main part of *norA*′ was cut out from pYU93 by *Eco*RI and *Eco*RV, and sequences upstream and downstream of the main part of *norA*′ were PCR-amplified. The three DNA fragments were ligated into the pCR-Blunt vector to obtain pHY125. The 11 kb DNA fragment was cut out from pHY125 by *Xba*I and *Eco*RI and ligated to pBU12^23^/*Xba*I + *Eco*RI fragment to yield pHY132. pHY129 and pHY132 were co-introduced into *S. albus* by triparental conjugation to generate *S. albus*::pHY129/pHY132 (*S. albus_nor*^*#*^ PKS).

### Construction of S. albus_nor* PKS

The *norA*′* fragment harboring the gene region coding for the *norA*′-AT2 domain to *norB*-KS4 domain, and the KS-AT linker in *aurB* was chosen as insertion site. The strategy to induce the *Hpa*I and *Spe*I restriction site in pHJ48 was the same as above, with primer pair Spec-aurB-fw (5′-CCACCTGATCCTCGAACAGGCCCCCGATGCGGAGGAGTCGGTTAACAT TCCGGGGATCCGTCGACC-3′) and Spec-aurB-rv (5′-GGATGCGTGCGGGCGCGCCGGACGCGGGCTCCGCGTCACTAGTTGTAGGCTGGAGCT GCTTC-3′), and the resulting *aadA* cassette was inserted into pHJ48-oriT to yield pHY133. A DNA fragment coding for the *norA*′-AT2 domain to *norB*-KS4 domain was cloned and inserted into the pCR-Blunt vector to produce pHY121. The 11-kb *Hpa*I/*Spe*I-digested DNA fragment of pHY121 was ligated into the *Hpa*I/*Spe*I fragment of pHY133. The resulting plasmid (pHY134) was introduced into *S. albus* by triparental conjugation to generate *S. albus*::pHY134 (S. albus_nor* PKS).

### Analysis of *nor* gene cluster in *S. scabrisporus* by PCR

Strain *S. scabrisporus* (DSM41855) was cultured in TSB medium (25 mL) with orbital shaking at 30 °C for 3 days. The genomic DNA was purified by the Wizard® Genomic DNA Purification Kit (Promega). The joint region between *norA*′ and *norC* was PCR-amplified using four primer pairs: NorAAfw-Ss (5′-TGCGATGGCGCTGCACGACG-3′)/NorCrv-Ss (5′-GACAACACACTGGCGGCGGC-3′), NorAAfw-Ss/NorAArv2-Ss (5′-CAACGACGCCGAACA CGCCG-3′), NorAAfw2-Ss (5′-GCCTGCTGGACTCGCTGCTG-3′)/NorCrv-Ss, and NorAAfw2-Ss/NorCrv2-Ss. NorAAfw-Ss and NorAAfw2-Ss are derived from *norA*′, while NorCrv-Ss and NorCrv2-Ss are derived from *norC*. The PCR reaction was performed by KAPA 2 G fast HotStart DNA polymerase (KAPABIOSYSTEMS) with 2.5% DMSO.

### General analytical procedures

NMR spectra of compounds dissolved in CDCl_3_ or CDCl_3_/CD_3_OD were measured on Bruker Avance DRX 500 or 600 MHz spectrometers equipped with a cryo probe at 300 K. The residual solvent peak (*δ*_H_ = 7.24, *δ*_C_ = 77.7) was used as reference. LC-HRMS measurements were carried out on a Thermo Fisher Scientific Exactive Orbitrap equipped with an electrospray ion source. Column: Betasil 100-3 C18 (2.1 × 150 mm); elution gradient: solvent A: water + 0.1% formic acid, solvent B: acetonitrile, gradient: 5% B for 1 min, 5–98% B in 15 min, 98% B for 3 min, flow rate: 0.2 mL min^−1^.

### HPLC analysis of *S. albus* mutant strains

To the bacterial culture 1 V ethyl acetate was added, and the mixture was stirred at room temperature overnight. Then, the organic phase was dried over sodium sulfate and concentrated to dryness under reduced pressure. The residue was dissolved in 1 mL methanol. The obtained extract (10 µL) was analyzed by reversed-phase column HPLC (Symmetry C18 5 µm, 4.6 × 150 mm, Waters) using a gradient program (solvent A (water + 0.1% trifluoroacetic acid) and solvent B (acetonitrile), 20% B for 5 min, to 99% B in 30 min and kept for 5 min at a flow rate of 1 mL min^−1^). Aureothin (350 nm), 7–deoxyaureothin (350 nm), 7-hydroxydeoxyaureothin (350 nm), neoaureothin (378 nm), intermediate (338 nm), and 7-deoxyneoaureothin (378 nm) productions were quantified by calculating peak area sizes compared to those of references.

### HPLC analysis of *S. scabrisporus*

*S. scabrisporus* DSM41855 was cultured on five PDA (potato dextrose agar) plates for 7 days. Agar was chopped and stirred in 100 mL ethyl acetate overnight. The extracts were dried over sodium sulfate and concentrated under reduced pressure. The residue was dissolved in 0.5 mL methanol. The reversed-phase HPLC analysis was performed as described above.

### Isolation of intermediate compound 5 from *S. albus* aur^§^_PKS

The ethyl acetate extract (1.5 L) of a *S. albus*::pNT42/pYU93 Δ*norA*′ (*aur*^*§*^_PKS) culture (1.5 L) was filtered through Celite 545. The water phase was further extracted with ethyl acetate (2 × 1.5 L). The combined extracts were dried over anhydrous sodium sulfate and concentrated under reduced pressure. The resulting residue was subjected to silica gel column chromatography (3 × 13 cm, silica gel 60, 0.040–0.063 mm) using a dichloromethane/methanol gradient. The dichloromethane-eluted fractions were subjected to preparative RP-HPLC (column, Nucleosil 100-7C18, 20 × 250 mm; flow rate, 16 mL min^−1^; gradient, solvent A (water containing 0.1% trifluoroacetic acid), B (83% acetonitrile); 20% B in 10 min to 100% B in 30 min and kept for 15 min) to yield **5** (1.0 mg). Pale yellow solid; HRMS: *m/z* [M–H]^−^ = 246.0770 (calculated for C_13_H_12_NO_4_, 246.0761); NMR data see Supplementary Table 8 and Supplementary Figs. 19–23.

### Isolation of compound 6 deoxyneoaureothin

A 2-L culture of *S. albus*::pNT42/pHY42 was extracted with ethyl acetate (3 × 2 L). The organic phases were combined, dried over sodium sulfate, and concentrated under reduced pressure. The extract was subjected to silica gel column chromatography (3 × 15 cm, Silica gel 60, 0.040–0.063 mm) using a dichloromethane/methanol gradient. Three fractions containing **6** were combined and subjected to preparative RP-HPLC (column, Supercosil 100-5C18, 21.2 × 250 mm; flow rate, 12 mL min^−1^; gradient, solvent A (40% acetonitrile), B (100% acetonitrile); 1% B in 10 min to 100% B in 30 min and kept for 10 min) to yield crude **6** (18.9 mg). The crude product was subjected to semipreparative RP-HPLC (column, Phenomenex Phenyl-Hexyl, particle size 5 µm, pore size 100 Å, 10 × 250 mm; flow rate, 6 mL min^−1^; gradient, solvent A (40% acetonitrile), B (100% acetonitrile), 1% B in 10 min to 100% B in 30 min and kept for 10 min) yielding deoxyneoaureothin (**6**) (7.7 mg). Yellow solid; HRMS: *m/z* [M + H]^+^ = 464.2426 (calculated for C_28_H_34_NO_5_, 464.2431); NMR data see Supplementary Table 4 and Supplementary Figs. 24–29.

### Isolation of congeners from *S. albus*_nor^#^ PKS

All isolation steps were carried out in the dark to avoid the degradation of polyketides. The (emulsion-forming) ethyl acetate extract (4.5 L) of a *S. albus* pHY134 (nor^#^_PKS) culture was filtered through Celite 545, and the water phase was again extracted with ethyl acetate (2 × 4.5 L). The combined extracts were dried over anhydrous sodium sulfate, concentrated under reduced pressure and subjected to chromatography on silica gel (column, 5 × 16 cm, Silica gel 60, 0.040–0.063 mm; gradient, dichloromethane/methanol). Further purification was achieved by preparative RP-HPLC (column, Nucleosil 100-7C18, 20 × 250 mm; flow rate, 16 mL min^−1^; gradient, solvent A (water containing 0.1% trifluoroacetic acid), B (83% acetonitrile); 40% B in 5 min to 100% B in 25 min and kept for 15 min) to yield crude **9**. This fraction was further subjected to semipreparative RP-HPLC (column, Nucleodur C18 HTec, 10 × 250 mm; flow rate, 6 mL min^−1^) using an isocratic system 83% acetonitrile/water containing 0.1% trifluoroacetic acid (80:20) to yield **9** (3.5 mg). NMR and MS data see Supplementary Figs. 18 and 41–46, Supplementary Table 12. A fraction containing **8** and **10** was subjected to preparative RP-HPLC (column, Nucleosil 100-7C18, 20 × 250 mm; flow rate, 12 mL min^−1^; gradient, solvent A (water containing 0.1% trifluoroacetic acid), B (83% acetonitrile); 20% B in 10 min to 100% B in 30 min and kept for 15 min) to yield crude **8** and **10**, respectively. Crude **10** was further purified by semipreparative RP-HPLC (column, Nucleodur C18 HTec, 10 × 250 mm; flow rate, 6 mL min^−1^; eluent, isocratic system 83% acetonitrile/water containing 0.1% trifluoroacetic acid, *v/v* 90:10) to yield **10** (5.5 mg). NMR and MS data see Supplementary Figs. 18 and 47–52, Supplementary Table 13. Crude **8** was further purified by semipreparative RP-HPLC (column, Nucleodur C18 HTec, 10 × 250 mm; flow rate, 6 mL min^−1^; eluent, isocratic system 83% acetonitrile/water containing 0.1% trifluoroacetic acid, *v/v* 80:20, followed by isocratic system composed of 83% acetonitrile/water containing 0.1% trifluoroacetic acid, *v/v* 70:30) to yield a mixture containing **8**. This fraction was further subjected to semipreparative RP-HPLC (column, Phenomenex Phenyl-Hexyl, 10 × 250 mm; flow rate, 6 mL min^−1^; eluent, isocratic system methanol/water containing 0.1% trifluoroacetic acid, *v/v* 80:20) to yield **8** (0.8 mg). NMR and MS data see Supplementary Figs. 18 and 35–40, Supplementary Table 11. The fraction containing **7** was subjected to preparative RP-HPLC (column, Nucleosil 100-7C18, 20 × 250 mm; flow rate, 16 mL min^−1^; gradient system, solvent A (water containing 0.1% trifluoroacetic acid), B (83% acetonitrile); 40% B in 5 min to 100% B in 25 min and kept for 15 min) to yield crude **7**. Final purification was achieved by another semipreparative RP-HPLC (column, Nucleodur C18 HTec, 10 × 250 mm; flow rate, 6 mL min^−1^; eluent, isocratic system 83% acetonitrile/water containing 0.1% trifluoroacetic acid, *v/v* 55:45) to yield **7** (2.7 mg). NMR and MS data see Supplementary Figs. 18 and 30–34, and Supplementary Table 10. Due to instability of pure congeners **8** to **10**, these compounds rapidly degraded after NMR measurements.

### Reporting summary

Further information on research design is available in the Nature Research Reporting Summary linked to this article.

## Supplementary information

Supplementary Information

Reporting Summary

Source Data file

## Data Availability

Data supporting the findings of this work are available within the paper and its Supplementary Information files. A reporting summary for this Article is available as a Supplementary Information file. The datasets generated and analyzed during the current study are available from the corresponding author upon request. The source data underlying Figs. 2c and 3b, as well as Supplementary Figs. 1 and 2 are provided as a Source Data file.
