# Supplementary material for: Emulating evolutionary processes to morph aureothin-type modular polyketide synthases and associated oxygenases
Source: Nat Commun. 2019 Sep 2;10:3918. doi: 10.1038/s41467-019-11896-1 (PMC6718629; doi:10.1038/s41467-019-11896-1)
Supplement: Supplementary file 1 — Supplementary Information [file 41467_2019_11896_MOESM1_ESM.pdf]

Emulating evolutionary processes to morph aureothin-type modular polyketide synthases and associated oxygenases

Peng *et al.*

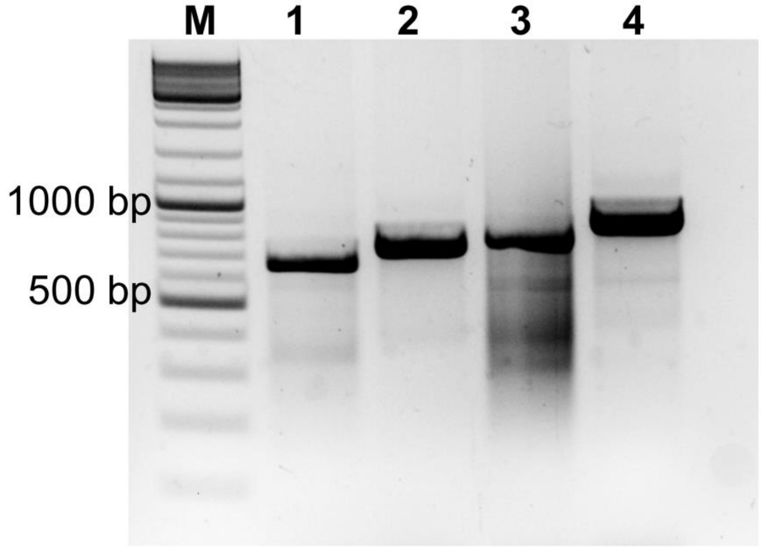

Supplementary Figure 1. PCR confirmation of the missing *norB* gene in the *nor* biosynthetic gene cluster in *Streptomyces scabrisporus* DSM41855. M: DNA size marker; primer pair (expected size): lane 1, NorAAfw-Ss/NorCrv-Ss (648 bp); 2, NorAAfw-Ss/NorCfw2-Ss (721 bp); 3, NorAAfw2-Ss/NorCfw-Ss (769 bp); and 4, NorAAfw2-Ss/NorCfw2-Ss (842 bp). Source data are provided as a Source Data file.

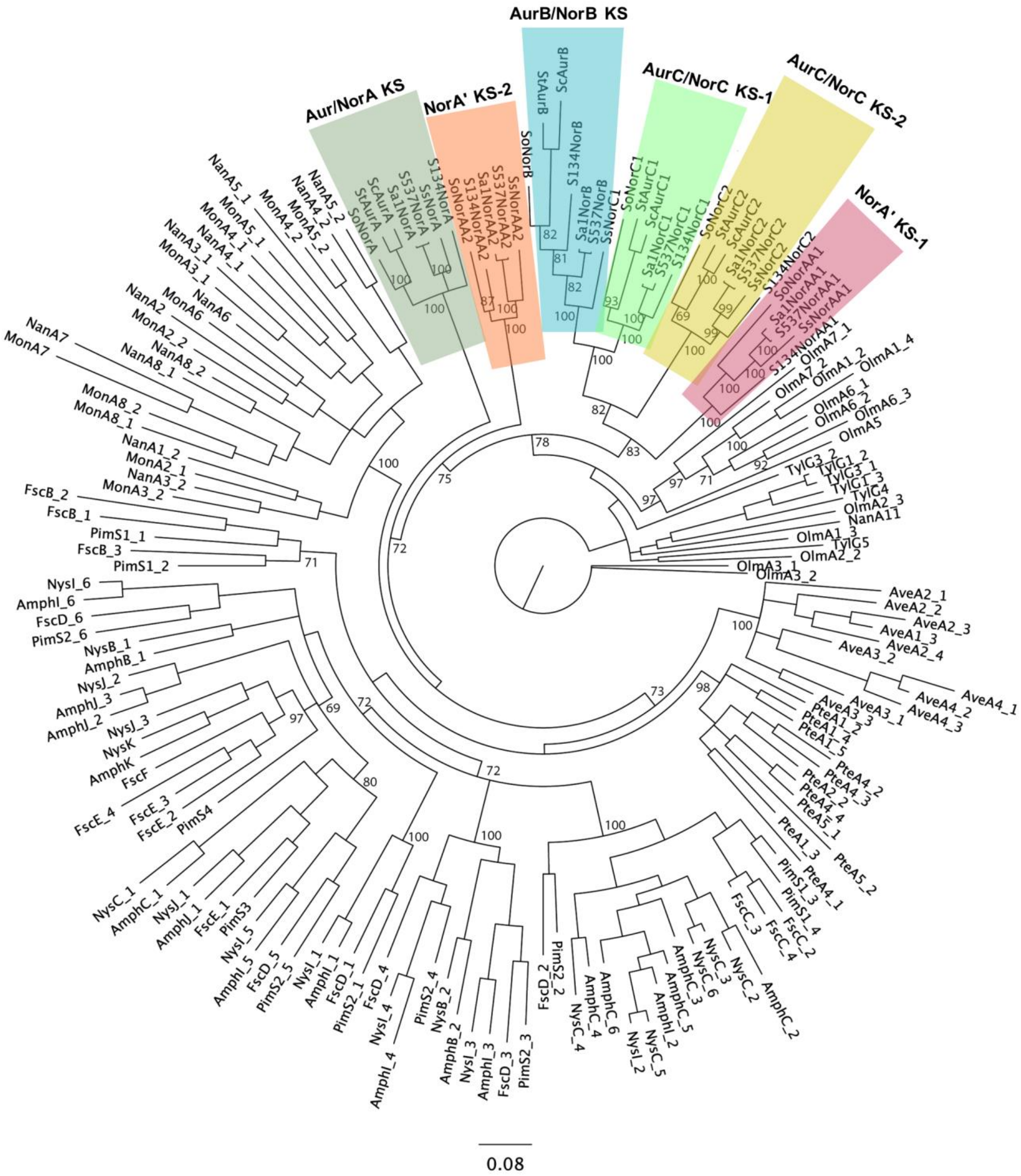

Supplementary Figure 2. Phylogenetic tree of the KS amino acid sequences of the *aur*-type and *nor*-type PKSs and selected other type I PKSs. The tree was reconstructed by Bayesian inference. Numbers at nodes indicated clade credibility values. Accession numbers of proteins used in this phylogenetic analysis are shown in Supplementary Table 2. Source data are provided as a Source Data file.

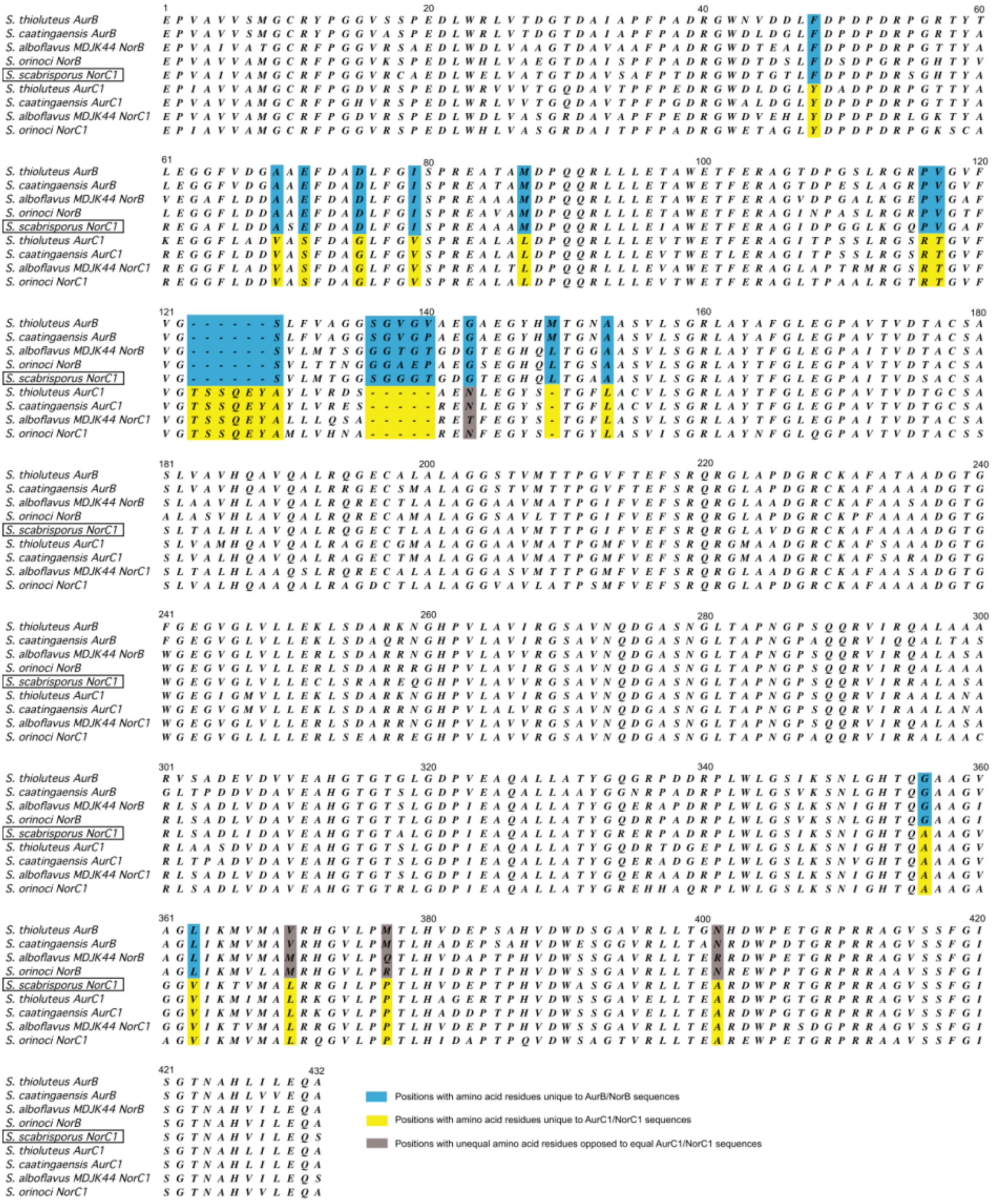

Supplementary Figure 3. Amino acid sequence alignments. Alignment of sequences of *S. scabrisporus* NorC-KS1 (NorC1) with selected AurB/NorB and AurC/NorC-KS1 (AurC1/NorC1) sequences. Columns with the same amino acid residue or indel pattern characteristic of AurB/NorB or NorC-KS1 sequences are highlighted in blue and yellow, respectively.

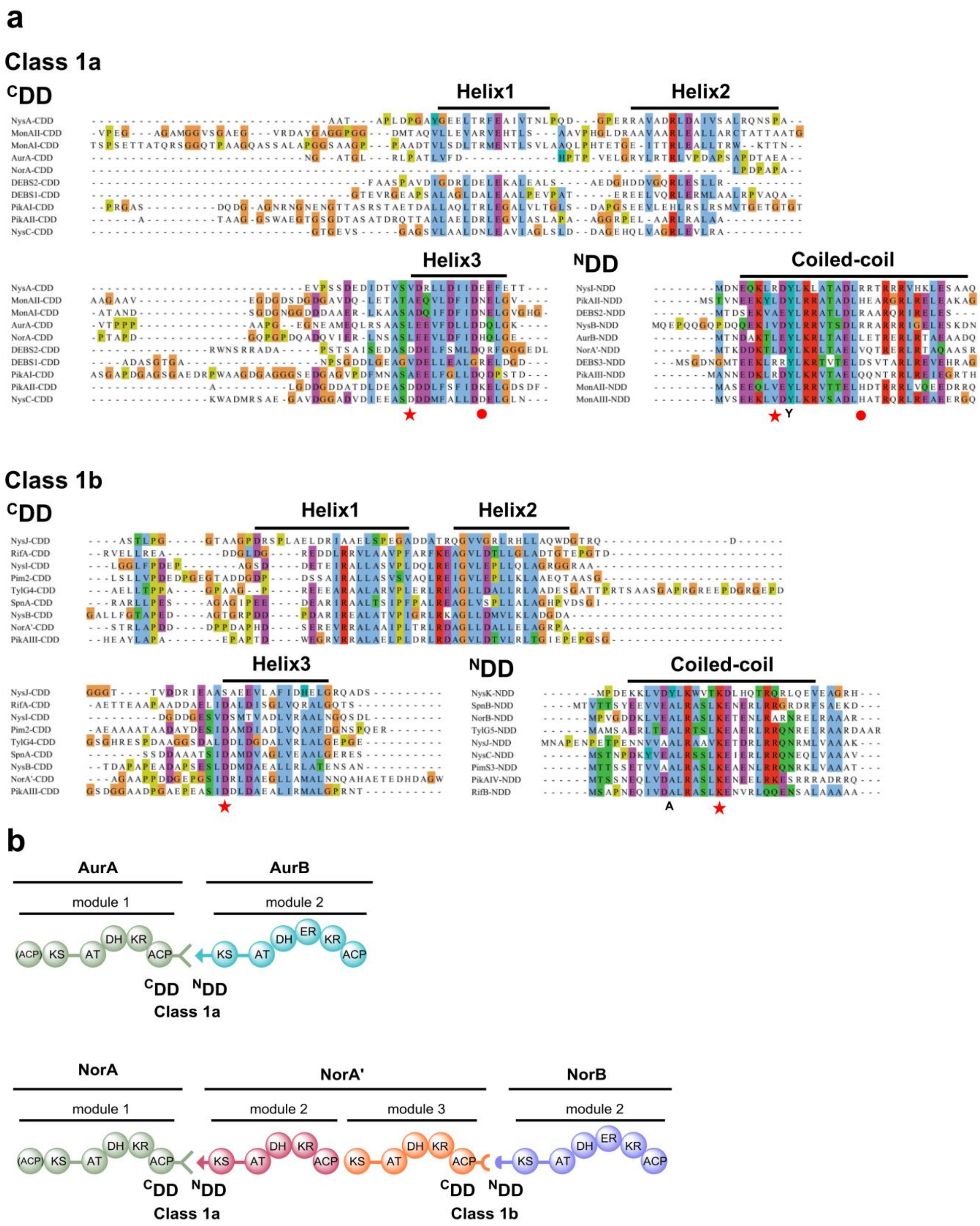

Supplementary Figure 4. The interactions between the type I polyketide C- and N-docking domains. a) Amino acid sequence alignment of docking domains in the *aur* PKS and *nor* PKS with other known docking domains; class 1a and class 1b[1-5]**.** The alignment is constructed by Clustal Omega[6] and shows default Clustal color sheme by Jalview 2.10.5[7] (http://www.jalview.org/). DEBS: erythromycin[8] (*Saccharopolyspora erythraea*), Pik: pikromycin[9] (*Streptomyces venezuelae*), Amph: amphotericin[10] (*Streptomyces nodosus*), Asm: ansamitocin[11] (*Actinosynnema pretiosum*), Nys: nystatin[12] (*Streptomyces noursei* ATCC 11455), Mon: monensin[13] (*Streptomyces cinnamonensis*), Spn: spinosad[14] (*Saccharopolyspora spinosa*), Tyl: tylactone[15] (*Streptomyces fradiae*), Pim: pimaricin[16] (*Streptomyces natalensis*). Rif: rifamycin[17] (*Amycolatopsis mediterranei*). Colored and

shaded boxes indicate basic residues (red), acidic residues (magenta), hydrophobic residues (blue), polar residues (green), aromatic residues (cyan), Gly (orange), and Pro (yellow). Potential charge-charge interactions between ^{C}DD (C-terminal docking domain) and ^{N}DD (N-terminal docking domain) are indicated by matching symbols (red star and circle). **Y** and **A** are well conserved amino acid in this position of ^{N}DD in class 1a and class 1b, respectively[5]. **b**) AurA/AurB and NorA/NorA' docking domains are a class 1a. On the other hand NorA'/NorB docking domain is a class 1b.

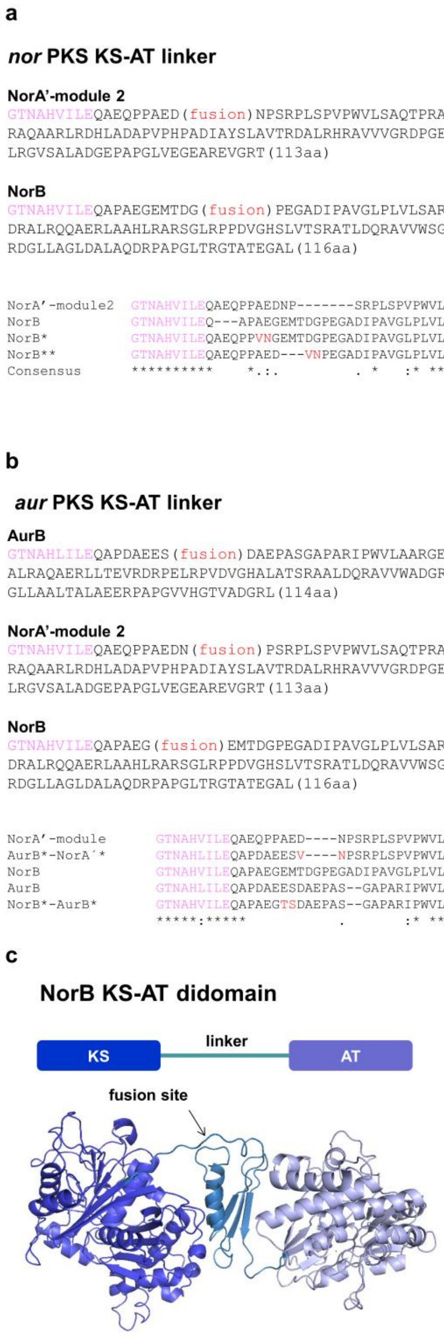

Supplementary Figure 5. Engineering strategy using KS-AT linker fusion in aureothin-type polyketides. **a**) Fusion sites in KS-AT linker applied in *nor*_PKS to *aur*_PKS engineering. The fusion sites induced by restriction enzyme *Hpa*I (VN) is a red letter. The length of original and recombinant KS-AT linker is shown in bracket. The 'optional' fusion site GTNAHVILE reported by Yuzawa *et al.*[18] is indicated in pink letter. The fusion sites chosen in this study are close to this 'optional' site. The resulting KS-AT linker amino acid sequence alignment is shown bottom. **b**) Fusion sites in KS-AT linker applied in *aur*_PKS to *nor*_PKS engineering. The fusion sites induced by restriction enzyme *Hpa*I (VN) and *Spe*I (TS) are red letters. The length of original and recombinant KS-AT linker is shown in bracket. The 'optional' fusion site GTNAHVILE reported by Yuzawa *et al.* is indicated in pink letter. The fusion sites chosen in this study are close to this 'optional' site. The resulting KS-AT linker amino acid sequence alignment is shown bottom. **c**) KS-AT didomain structural model of

NorB. The amino acid sequences of NorB, AurB*, AurB**, AurB, AurB*-NorA'*-module2 and NorB*-AurB*-module4 were submitted to phyre2[19] for modeling. Obtained the most similar structure model is the mammal fatty acid structure 2VZ8[20] (88% coverage, 28% identity). All the six models are based on 2VZ8 and then showed the same 3D structure model.

a

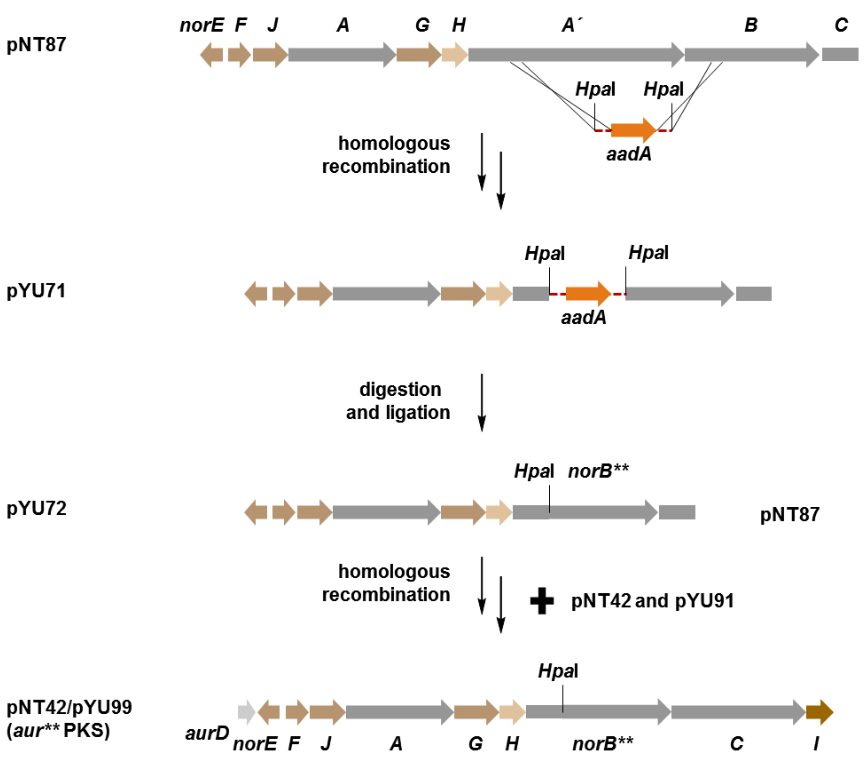

b

fusion site in KS-AT linker:

norA´-module2
-CCC CCG GCC GAG GAC AAC CCC AGC CGG CCG CTC TCC CCG-
P P A E D N P S R P L S P

norB
-GAA GGG GAG ATG ACG GAC GGG CCG GAG GGC GCG GAC ATC-
E G E M T D G P E G A D I

*norB***
(*Hpa*I)
-CCC CCG GCC GAG GAC GTT AAC CCG GAG GGC GCG GAC ATC-
P P A E D V N P E G A D I

Supplementary Figure 6. Schematic strategy to generate the *aur* PKS. a**) Workflow to generate the *aur*** PKS: *norA'* and *norB* are fused at the KS-AT linker of *norA'*-module 2. **b**) Nucleotide/amino acid sequences of the original and fusion sites in the KS-AT linker regions. The fusion site *Hpa*I is highlighted in red.

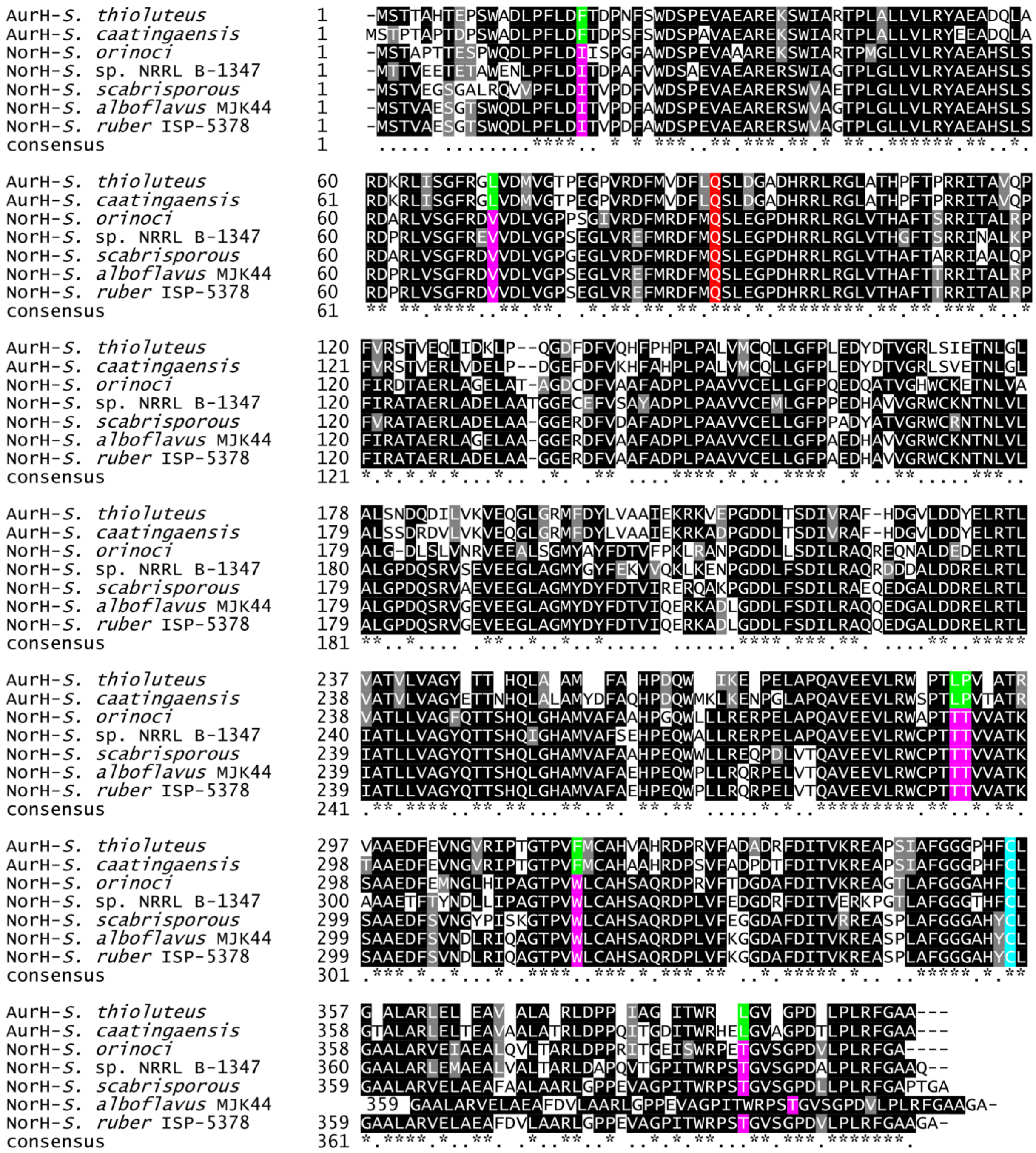

Supplementary Figure 7. Amino acid sequence alignment of AurH and homologues. The alignment is constructed by Clustal Omega and shaded similar (gray)/identical (black) residues by BoxShade (https://embnet.vital-it.ch/software/BOX_form.html). The same residues and similar residues are indicated by star and dot, respectively. The catalytic glutamine is highlighted in red and the conserved cysteine binding to heme is in cyan. The residues important for the size of the catalytic cavity are in green (AurH homologues) and purple (NorH homologues), and they are conserved in AurH and NorH, respectively.

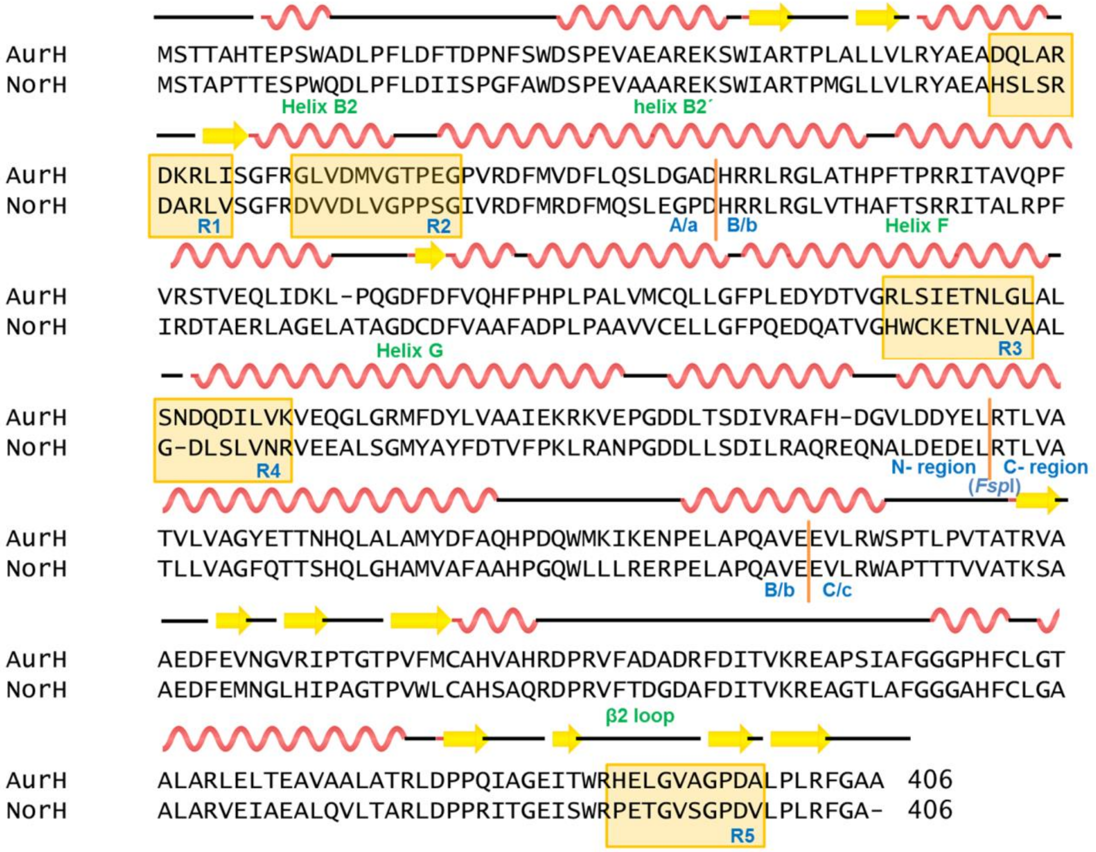

Supplementary Figure 8. Strategies to construct chimeric NorH variants and hybrid NorH/AurH variants. Five swapped regions of NorH are shaded in orange. The combination sites for hybrid NorH/AurH variants constructs are indicated by vertical orange lines.

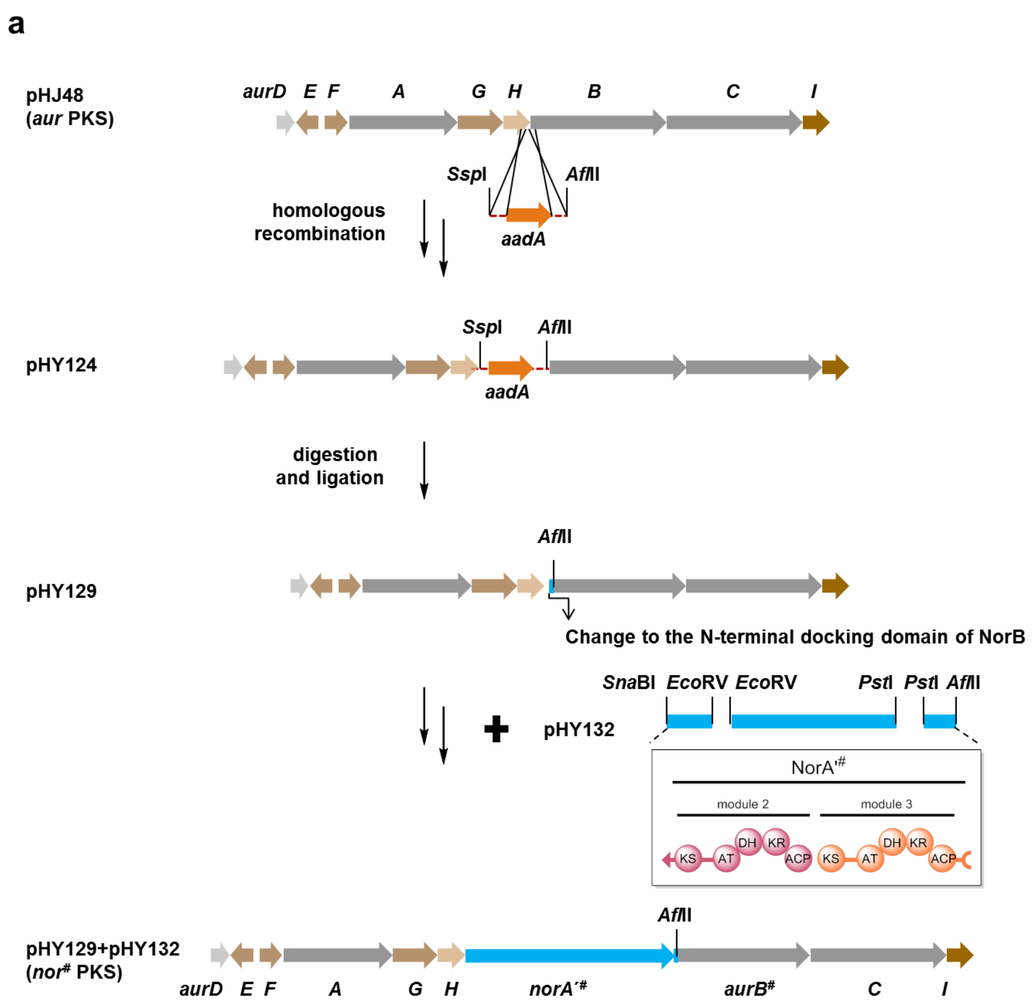

b

fusion site in docking domain:

aurB	-CGC ACC GCG GAG GCC GCG GAC CAG GAG CCC GTC GCG GTG GTG TCA ATG-
	R T A E A A D Q E P V A V V S M
norB	-CGG GAG CTG CGG GCC GCC GCC CGG GAA CCG GTG GCG GTG GTG GCC ATG-
	R E L R A A A R E P V A V V A M
	(*Afl*II)
aurB#	-CGG GAG CTG CGG GCC GCC CTT AAG GAG CCC GTC GCG GTG GTG TCA ATG-
	R E L R A A L K E P V A V V S M

Supplementary Figure 9. Schematic strategy to generate the *nor*# PKS. a) Workflow to generate the *nor*# PKS: the N-terminal docking domain of *aurB* is swapped with that of *norB* to generate pHY129, and *norA´*# including the N- and C-terminal docking domains is expressed separately as pHY132. **b**) Nucleotide/amino acid sequences of the original and fusion sites in the docking domain regions. The fusion site *Afl*II is highlighted in red.

a

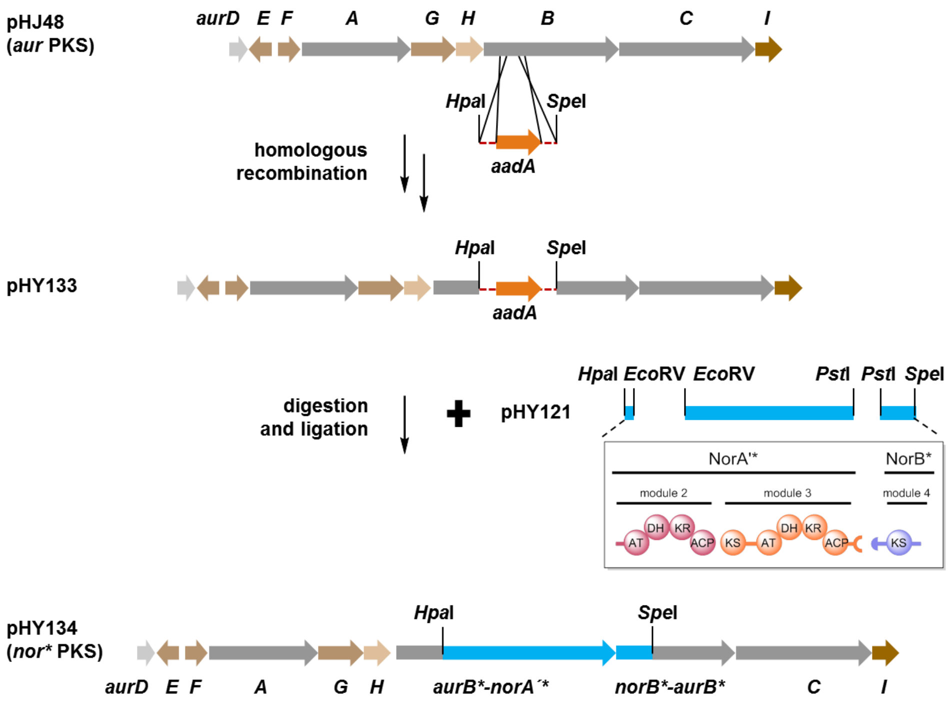

b

fusion site in KS-AT linker:

aurB	-CCC GAT GCG GAG GAG TCG GAC GCG GAG CCC GCG TCC GGC GCG CCC-
	P D A E E S D A E P A S G A P
	(*Hpa*I)
aurB*-norA´*	-CCC GAT GCG GAG GAG TCG GTT AAC CCC AGC CGG CCG CTC TCC-
	P D A E E S V N P S R P L S
	(*Spe*I)
norB*-aurB*	-CCG GCA GAA GGG ACT AGT GAC GCG GAG CCC GCG TCC GGC GCG CCC-
	P A E G T S D A E P A S G A P

Supplementary Figure 10. Schematic strategy to generate the *nor PKS. a**) Workflow to generate the *nor** PKS: the region between the *norA'*-AT2 and *norB*-KS4 (*norA'**-*norB**) is inserted into the KS-AT linker of *aurB* to generate pHY134. **b**) Nucleotide/amino acid sequences of the original and fusion sites in the KS-AT linker regions. The fusion sites *Hpa*I and *Spe*I are highlighted in red.

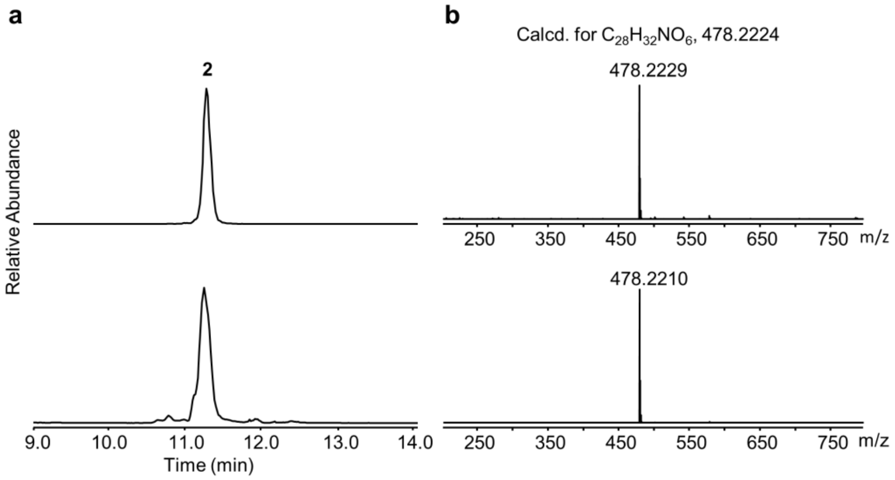

Supplementary Figure 11. The HPLC-HRMS profiles at extracted ion chromatogram. **a**) The HPLC-HRMS profile at EIC *m/z* 477.70−478.70 $(M+H)^+$ of authentic reference of neoaureothin (**2**, upper panel), and the ethyl acetate extracts from *S. scabrisporus* culture (lower panel). **b**) Observed ions corresponding to those of **a**.

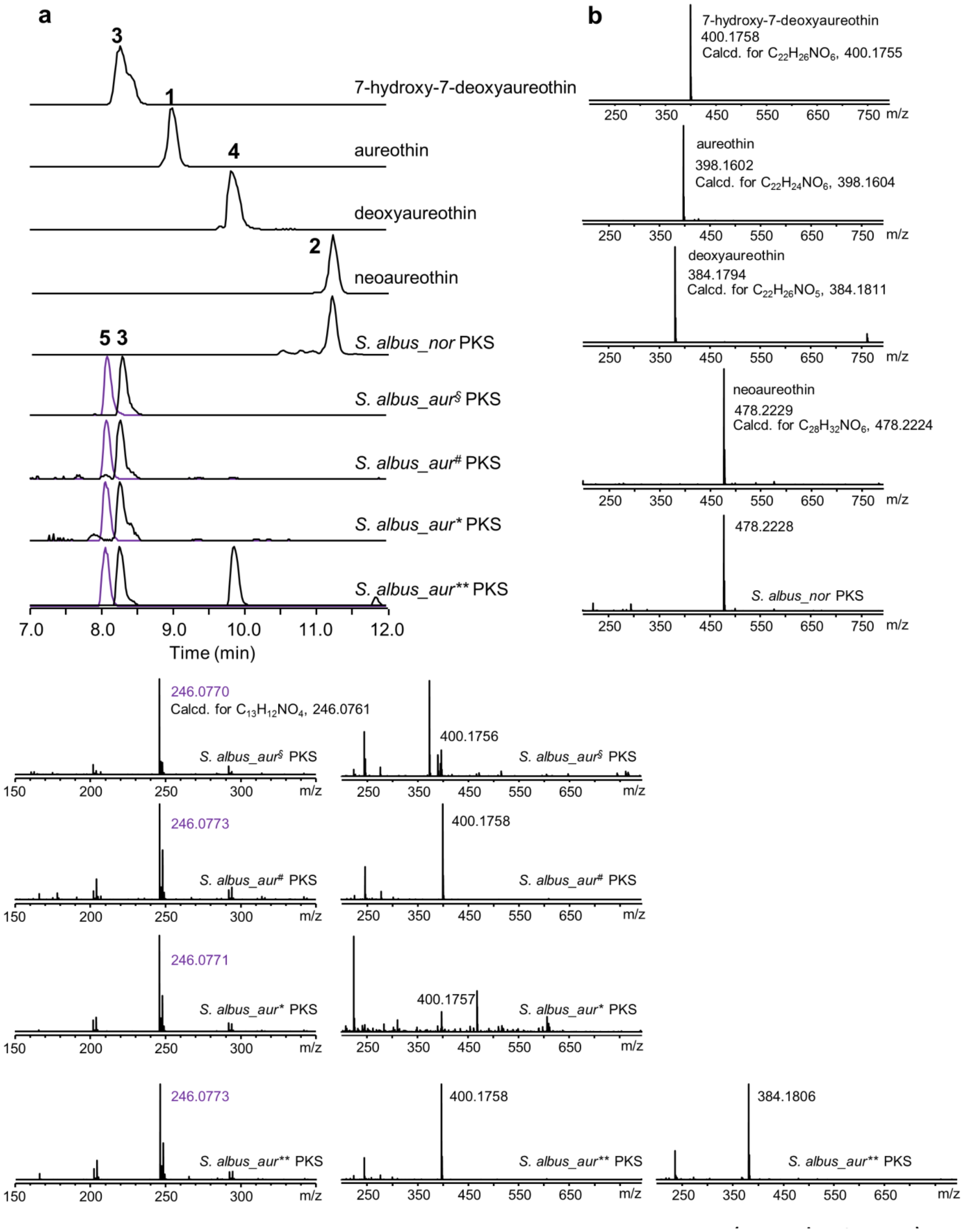

Supplementary Figure 12. HPLC-HRMS profiles at extracted ion chromatogram. **a**) The HPLC-HRMS profile at EIC *m/z* 399.67-400.67 $(M+H)^+$ of authentic reference of 7-hydroxy-7-deoxyaureothin (**3**), *S. albus_aur*§ PKS, *S. albus_aur*# PKS, *S. albus_aur** PKS, *S. albus_aur*** PKS, EIC *m/z* 397.65-398.65 $(M+H)^+$ of authentic reference of aureothin (**1**), EIC *m/z* 477.72-478.72 $(M+H)^+$ of authentic reference of neoaureothin (**2**) and *S. albus_nor* PKS, EIC *m/z* 383.68-384.68 $(M+H)^+$ of authentic reference of 7-deoxyaureothin (**4**) and *S.*

*albus_aur*** PKS, EIC *m/z* 245.57-246.57 $(M-H)^-$ for intermediate (**5**, purple), of *S. albus_aur*§ PKS, *S. albus_aur*# PKS, *S. albus_aur** PKS, *S. albus_aur*** PKS. **b**) Observed ions corresponding to those of **a**.

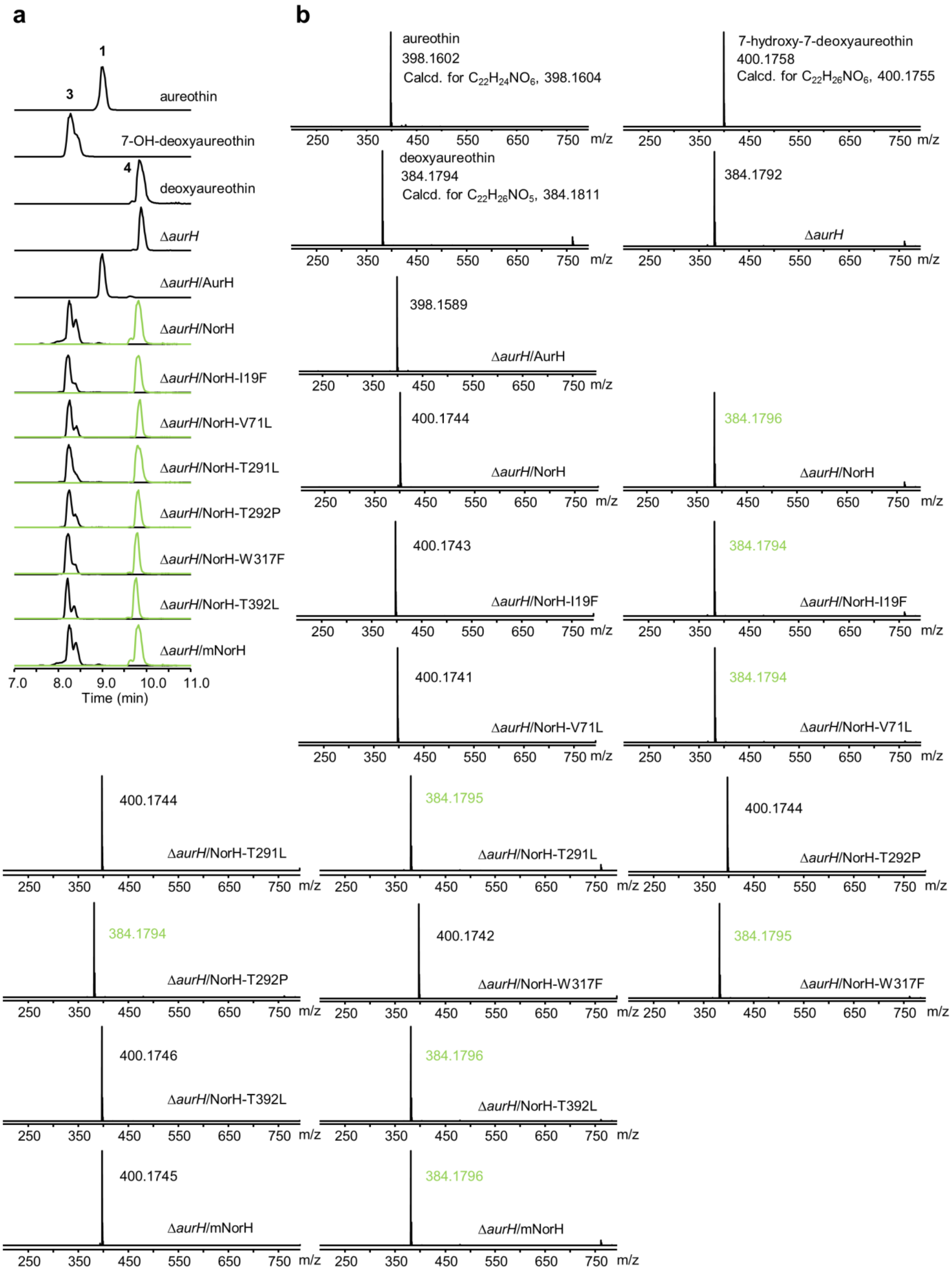

Supplementary Figure 13. The HPLC-HRMS profiles at extracted ion chromatogram (EIC). a) The HPLC-HRMS profile at EIC *m/z* 397.65-398.65 $(M+H)^+$ of authentic reference of aureothin (**1**) and Δ*aurH*/AurH, EIC *m/z* 399.67-400.67 $(M+H)^+$ of authentic reference of 7-OH-deoxyaureothin (**3**), Δ*aurH*/NorH, Δ*aurH*/NorH-I19F, Δ*aurH*/NorH-V71L, Δ*aurH*/NorH-T291L, Δ*aurH*/NorH-T292P, Δ*aurH*/NorH-W317F, Δ*aurH*/NorH-T392L, Δ*aurH*/multiple-point-

mutated NorH, EIC *m/z* 383.67-384.67 $(M+H)^+$ of 7-deoxyaureothin (**4**, yellowgreen), Δ*aurH*, Δ*aurH*/NorH, Δ*aurH*/NorH-I19F, Δ*aurH*/NorH-V71L, Δ*aurH*/NorH-T291L, Δ*aurH*/NorH-T292P, Δ*aurH*/NorH-W317F, Δ*aurH*/NorH-T392L, Δ*aurH*/multiple-point-mutated NorH. **b**) Observed ions corresponding to those of **a**.

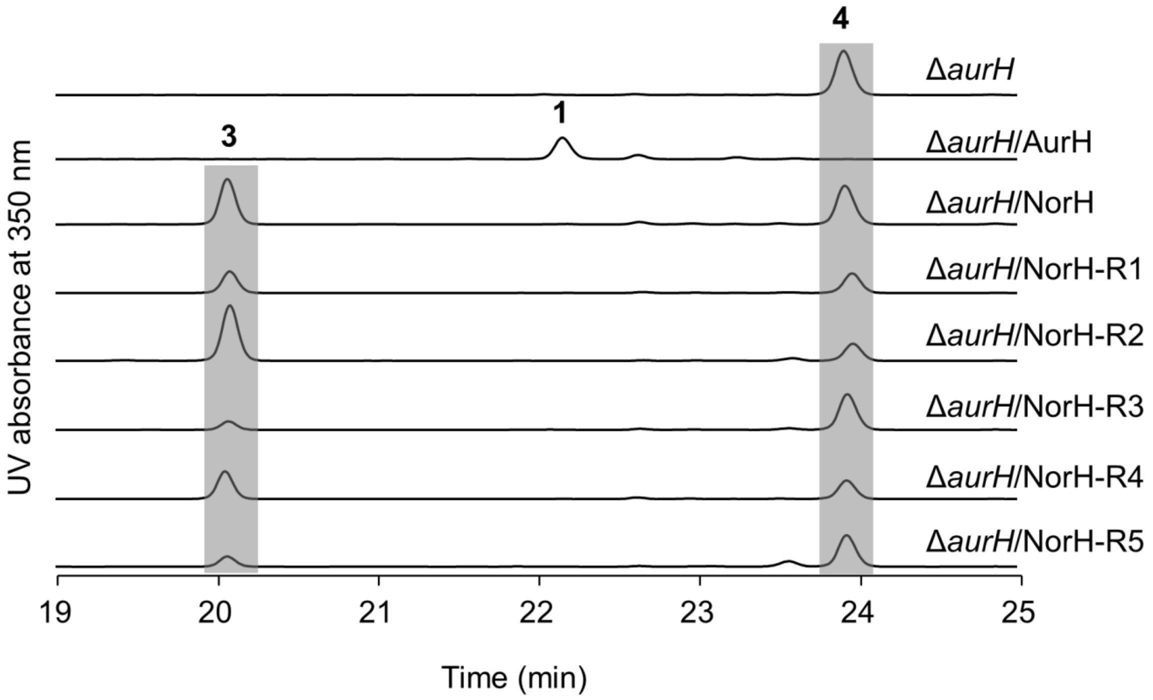

Supplementary Figure 14. HPLC profile of region-swapped chimeric NorHs. The EtOAc extracts of*S. albus* pHJ68 (*ΔaurH*), *ΔaurH*/AurH, *ΔaurH*/NorH, *ΔaurH*/NorH-R1, *ΔaurH*/NorH-R2, *ΔaurH*/NorH-R3, *ΔaurH*/NorH-R4, and *ΔaurH*/NorH-R5. mutant strains.. Aureothin (**1**), 7-OH deoxyaureothin (**3**), 7-deoxyaureothin (**4**).

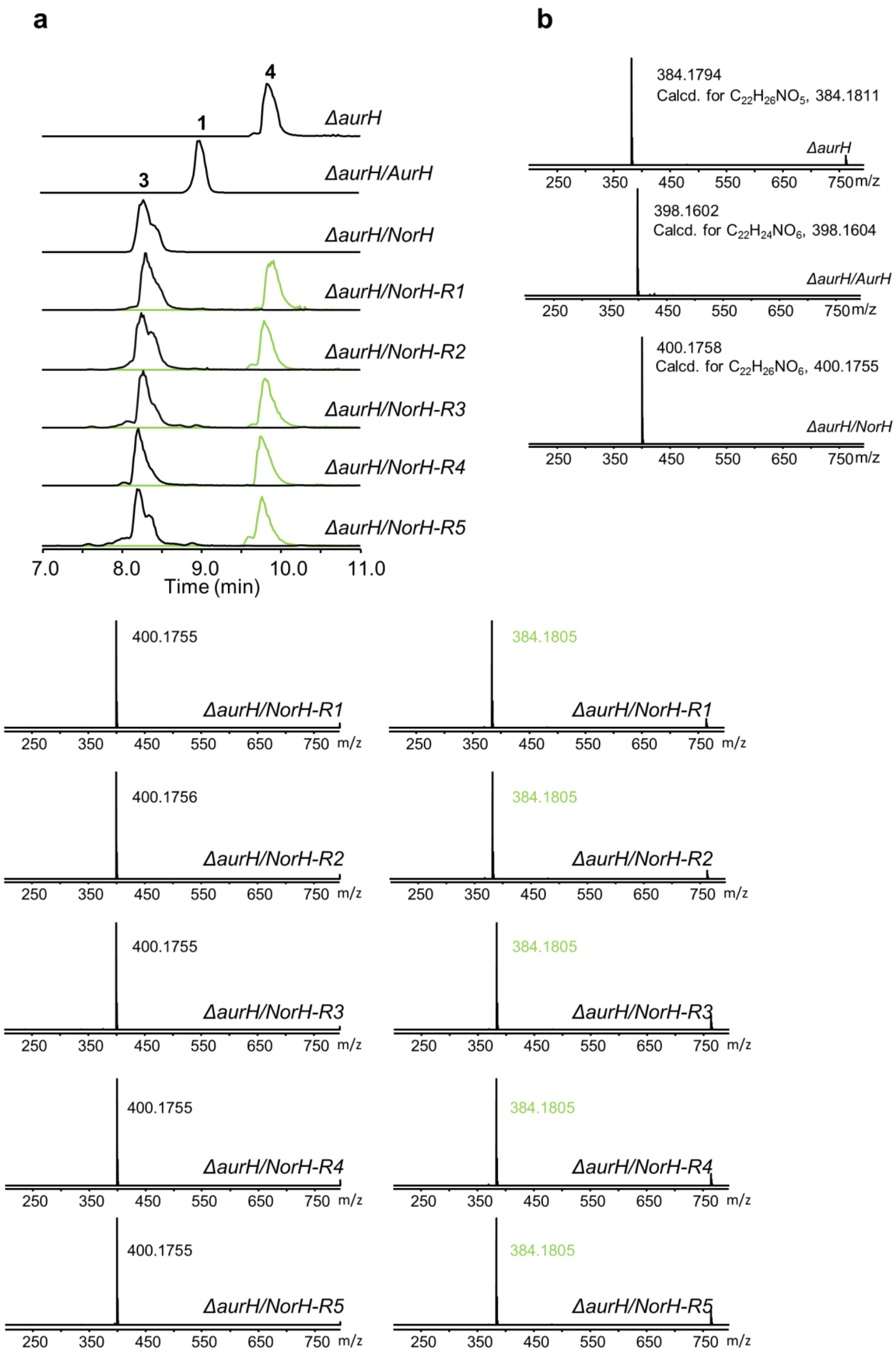

Supplementary Figure 15. The HPLC-HRMS profiles of catalytic activities of region-swapped chimeric NorH variants at extracted ion chromatogram. a) The HPLC-HRMS profile at EIC *m/z* 383.67-384.67 $(M+H)^+$ of authentic reference of 7-deoxyaureothin (**4**, yellowgreen), Δ*aurH*/NorH-R1, Δ*aurH*/NorH-R2, Δ*aurH*/NorH-R3, Δ*aurH*/NorH-R4, and Δ*aurH*/NorH-R5, EIC *m/z* 397.65-398.65 $(M+H)^+$ of authentic reference of aureothin (**1**), EIC

m/z 399.67-400.67 $(M+H)^+$ of authentic reference of 7-OH-deoxyaureothin (**3**), Δ*aurH*/NorH-R1, (v) Δ*aurH*/NorH-R2, Δ*aurH*/NorH-R3, Δ*aurH*/NorH-R4, and Δ*aurH*/NorH-R5. **b**) Observed ions corresponding to those of **a**.

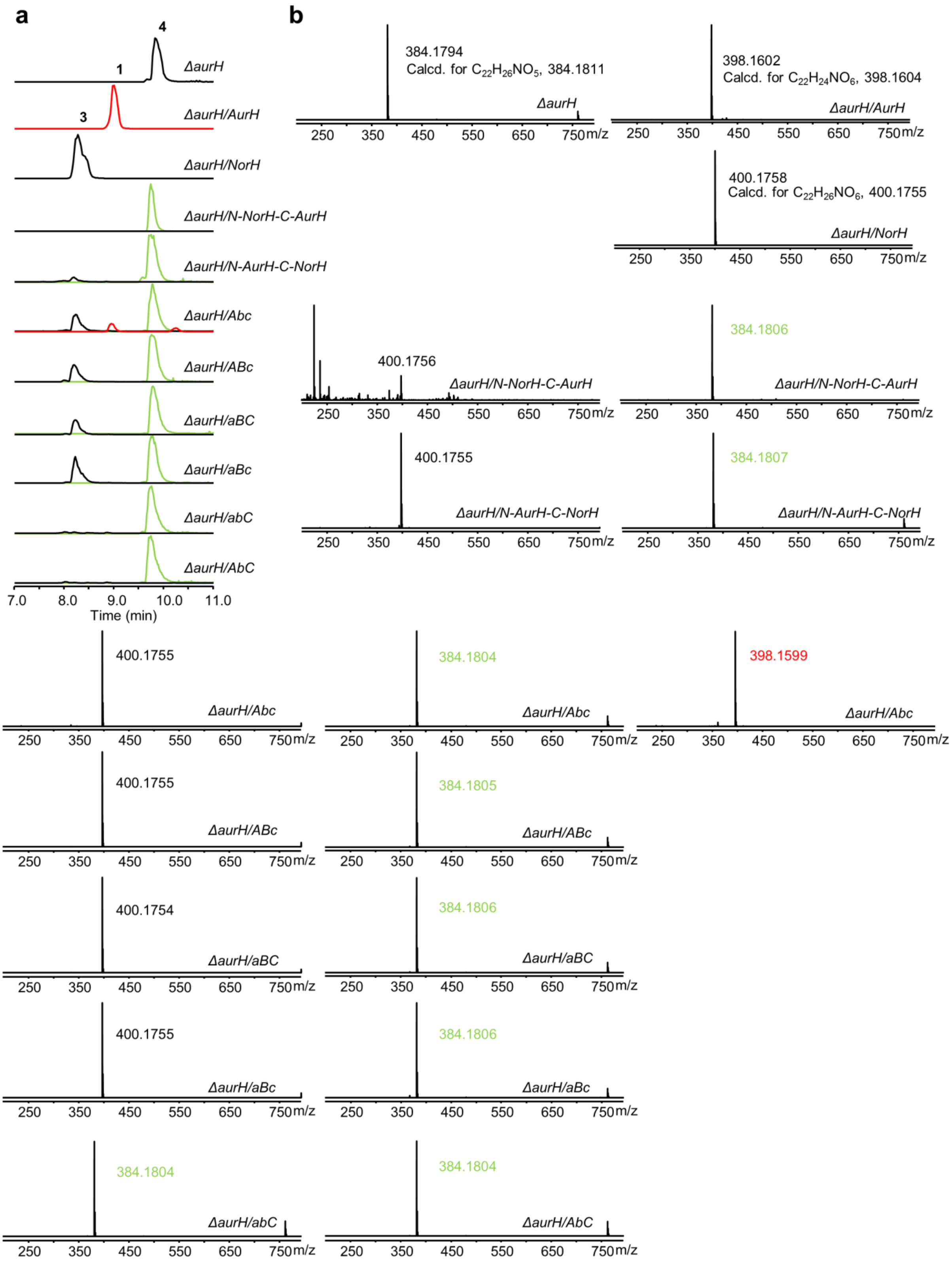

Supplementary Figure 16. The HPLC-HRMS profiles of catalytic activities of hybrid NorH variants at extracted ion chromatogram. **a**) The HPLC-HRMS profile at EIC *m/z* 383.67-384.67 $(M+H)^+$ of authentic reference of 7-deoxyaureothin (**4**, yellowgreen), Δ*aurH*/N-NorH-C-AurH, Δ*aurH*/N-AurH-C-NorH, Δ*aurH*/Abc, Δ*aurH*/ABc, and Δ*aurH*/aBC, Δ*aurH*/aBc, Δ*aurH*/abC, and Δ*aurH*/AbC, EIC *m/z* 397.65-398.65 $(M+H)^+$ of authentic reference of aureothin (**1**, red), and Δ*aurH*/Abc, EIC *m/z* 399.67-400.67 $(M+H)^+$ of authentic reference of

7-OH-deoxyaureothin (**3**), Δ*aurH*/N-NorH-C-AurH, Δ*aurH*/N-AurH-C-NorH, Δ*aurH*/Abc, Δ*aurH*/ABc, and Δ*aurH*/aBC, Δ*aurH*/aBc. **b**) Observed ions corresponding to those of **a**.

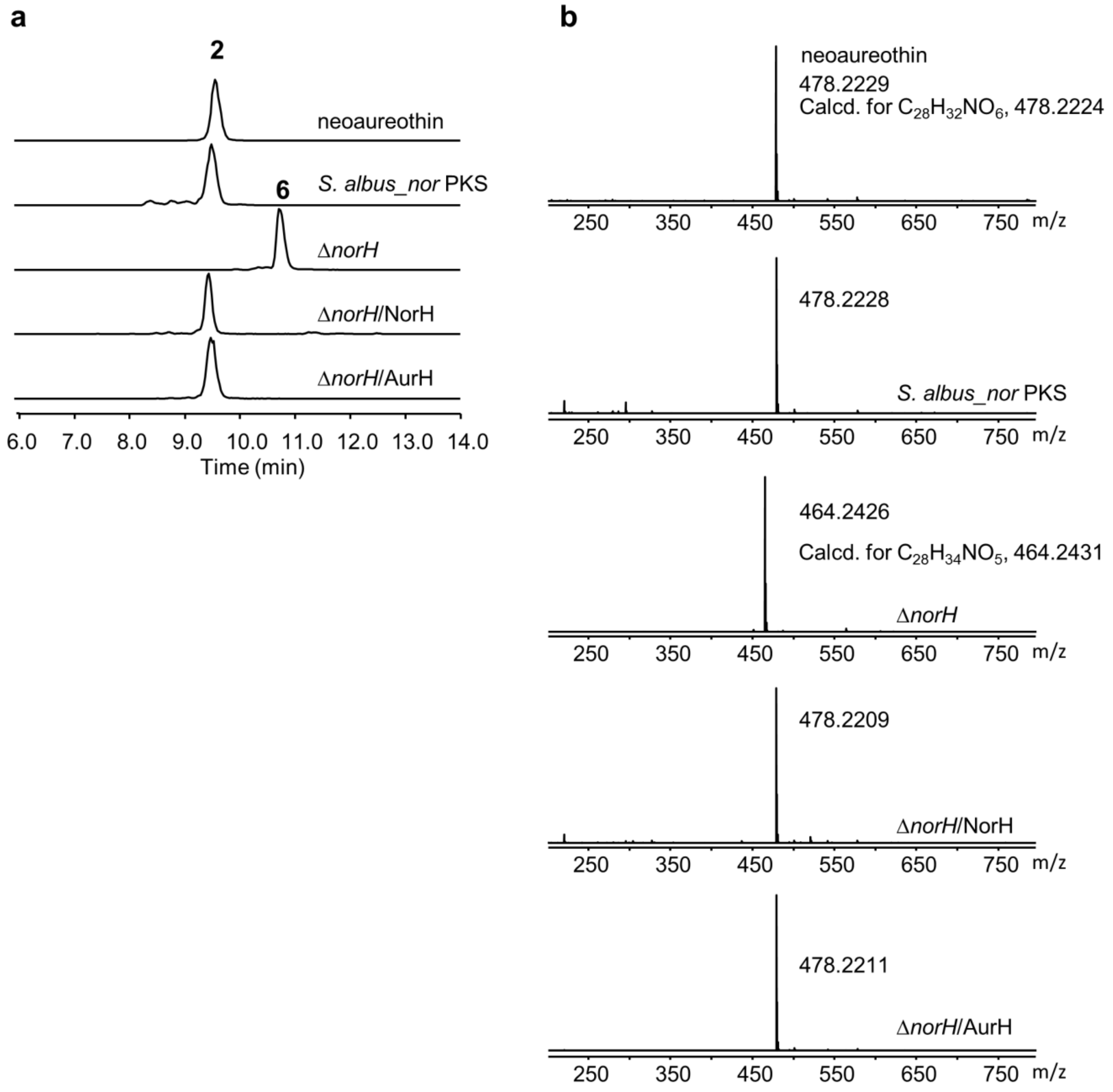

Supplementary Figure 17. The HPLC-HRMS profiles of catalytic activities of NorH at extracted ion chromatogram. a) The HPLC-HRMS profile at EIC *m/z* 477.72−478.72 $(M+H)^+$ of authentic reference of neoaureothin (**2**), and *S. albus_nor* PKS, Δ*norH*/*norH*, Δ*norH*/*aurH,* EIC *m/z* 463.74−464.74 $(M+H)^+$ of *S. albus_nor* PKS Δ*norH*. Deoxyneoaureothin (**6**). **b**) Observed ions corresponding to those of **a**.

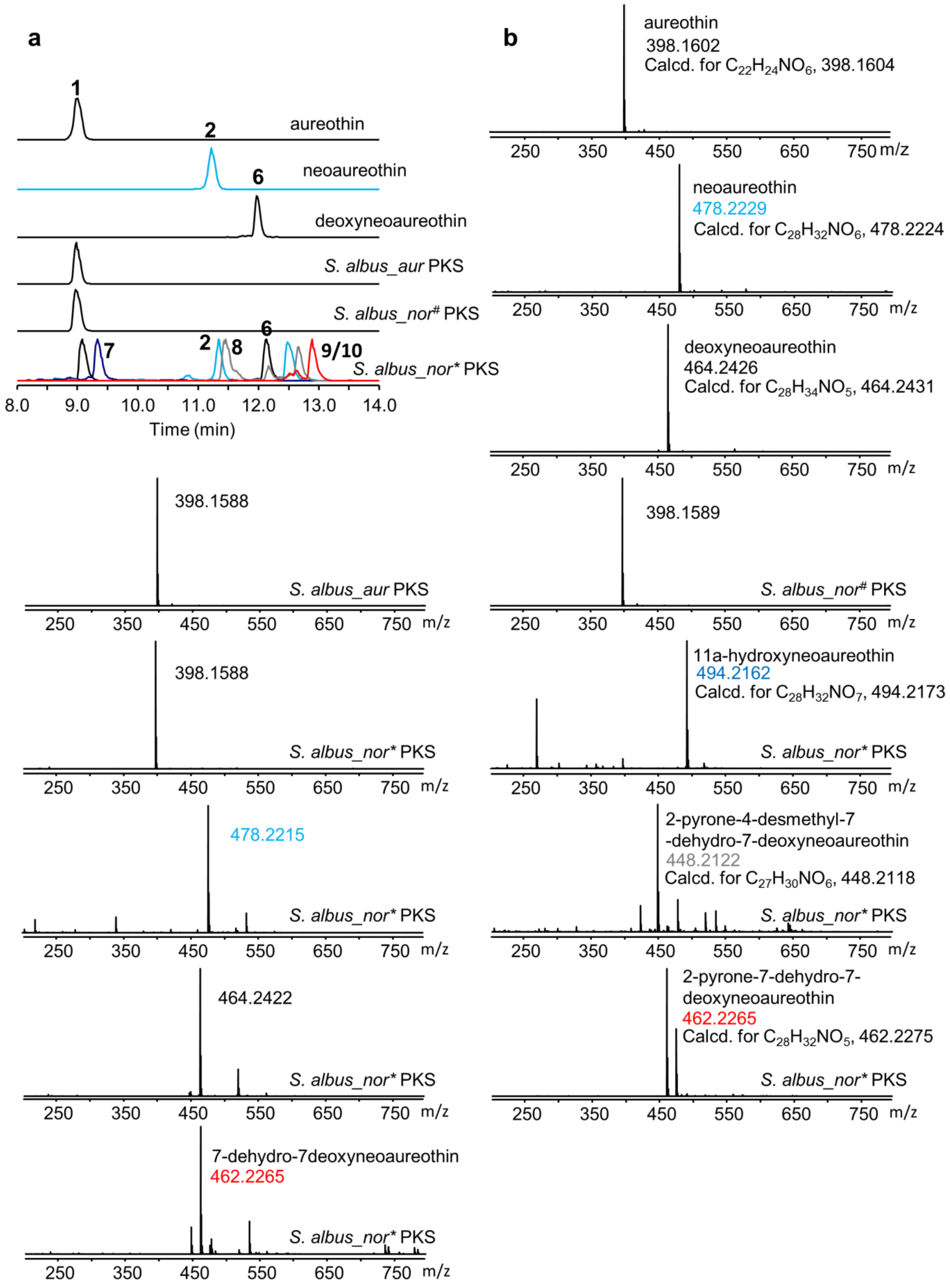

Supplementary Figure 18. The HPLC-HRMS profiles of catalytic activities of NorH at extracted ion chromatogram. a), The HPLC-HRMS profile at EIC *m/z* 397.65−398.65 $(M+H)^+$ of authentic reference of aureothin (**1**), and *S. albus_aur* PKS, *S. albus_nor*# PKS, *S. albus_nor** PKS, EIC *m/z* 477.72-478.72 $(M+H)^+$ of authentic reference of neoaureothin (**2**, skyblue) and *S. albus_nor** PKS, EIC *m/z* 463.73-464.73 $(M+H)^+$ of authentic reference of deoxyneoaureothin (**6**) and *S. albus_nor** PKS, EIC *m/z* 493.71-494.71 $(M+H)^+$, 11a-hydroxyneoaureothin (**7**, navy), of *S. albus_nor** PKS, at EIC *m/z* 447.70-448.70 $(M+H)^+$, 2-

pyrone-4-desmethyl-7-dehydro-7-deoxyneoaureothin (**8**, grey), of *S. albus_nor** PKS, EIC *m/z* 461.72-462.72 $(M+H)^+$, 2-pyrone-7-dehydro-7deoxyneoaureothin (**9**, red) and 7-dehydro-7deoxyneoaureothin (**10**, red) of *S. albus_nor** PKS. **b**), Observed ions corresponding to those of **a**.

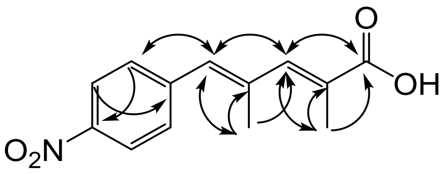

Supplementary Figure 19. Structure and selected HMBC correlations of intermediate 5. HMBC correlations are indicated by arrows.

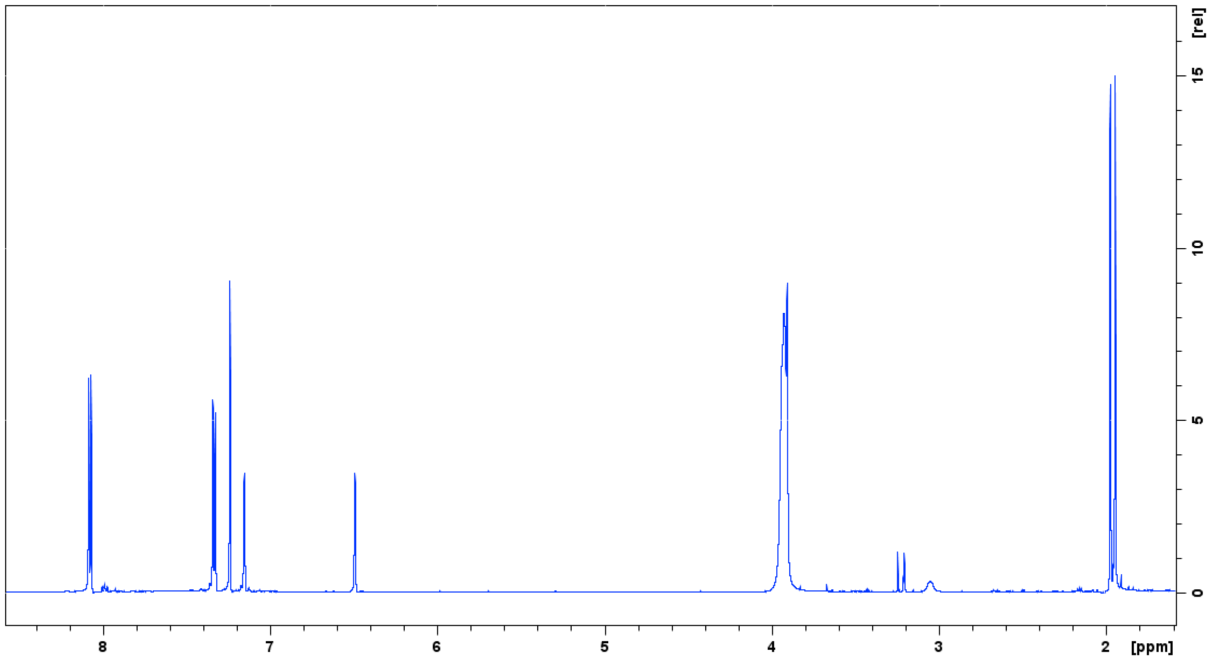

Supplementary Figure 20. ^{1}H NMR spectrum of intermediate 5. Solvent $CDCl_3/CD_3OD$ (*v/v* 2:1).

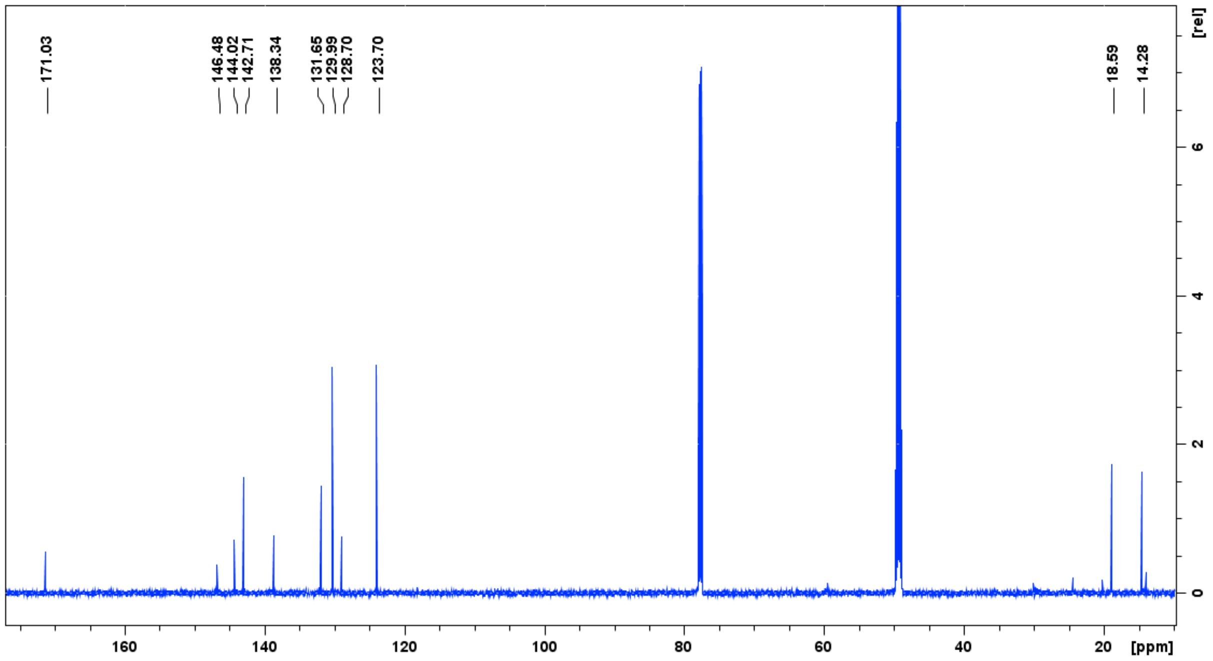

Supplementary Figure 21. ^{13}C NMR spectrum of intermediate 5. Solvent $CDCl_3$/CD_3OD (*v/v* 2:1).

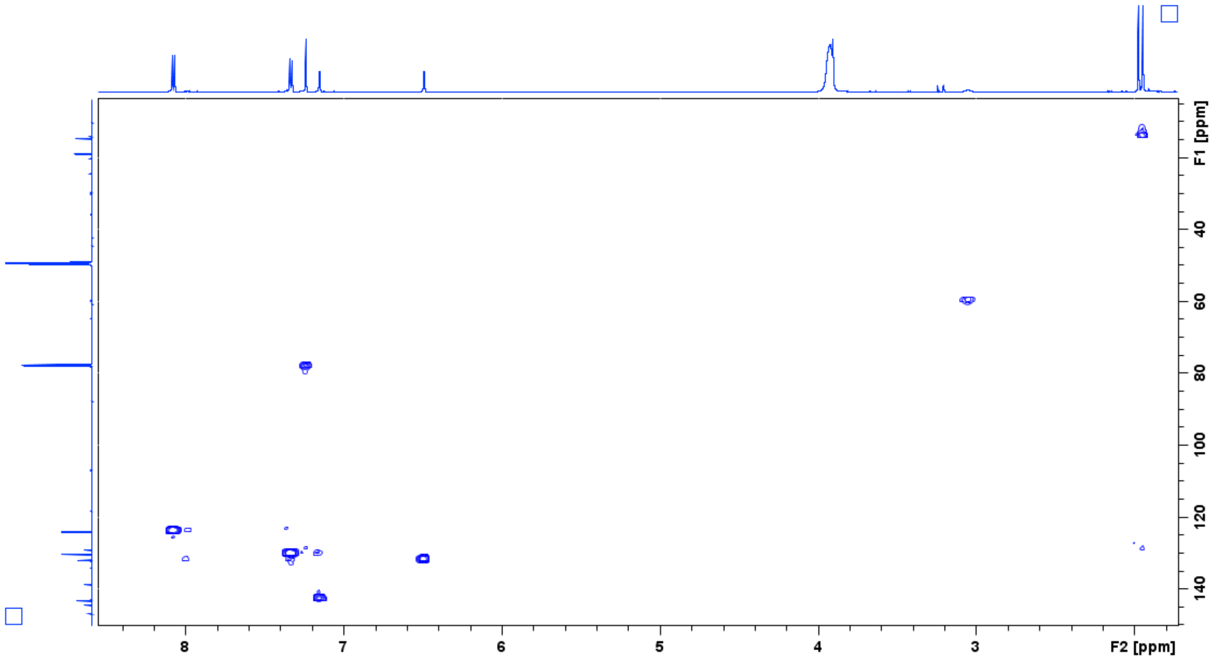

Supplementary Figure 22. HSQC spectrum of intermediate 5. Solvent $CDCl_3/CD_3OD$ (*v/v* 2:1).

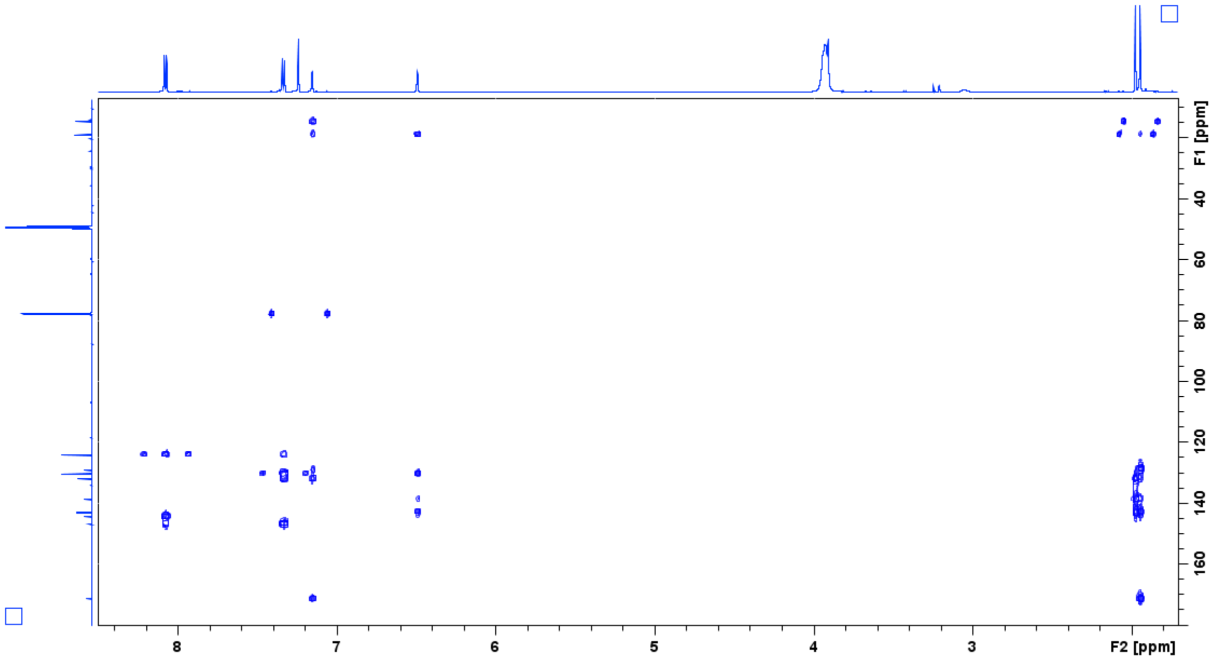

Supplementary Figure 23. HMBC spectrum of intermediate 5. Solvent $CDCl_3/CD_3OD$ (*v/v* 2:1).

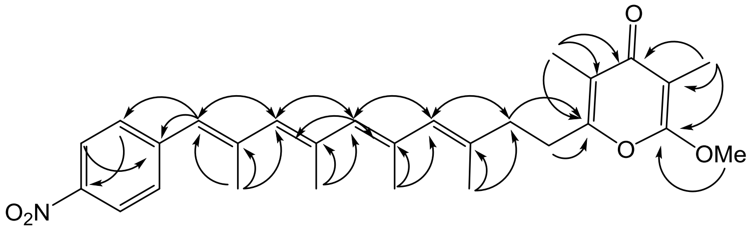

Supplementary Figure 24. Structure and selected HMBC correlations of deoxyneoaureothin. HMBC correlations indicated by arrows, compound number **6**.

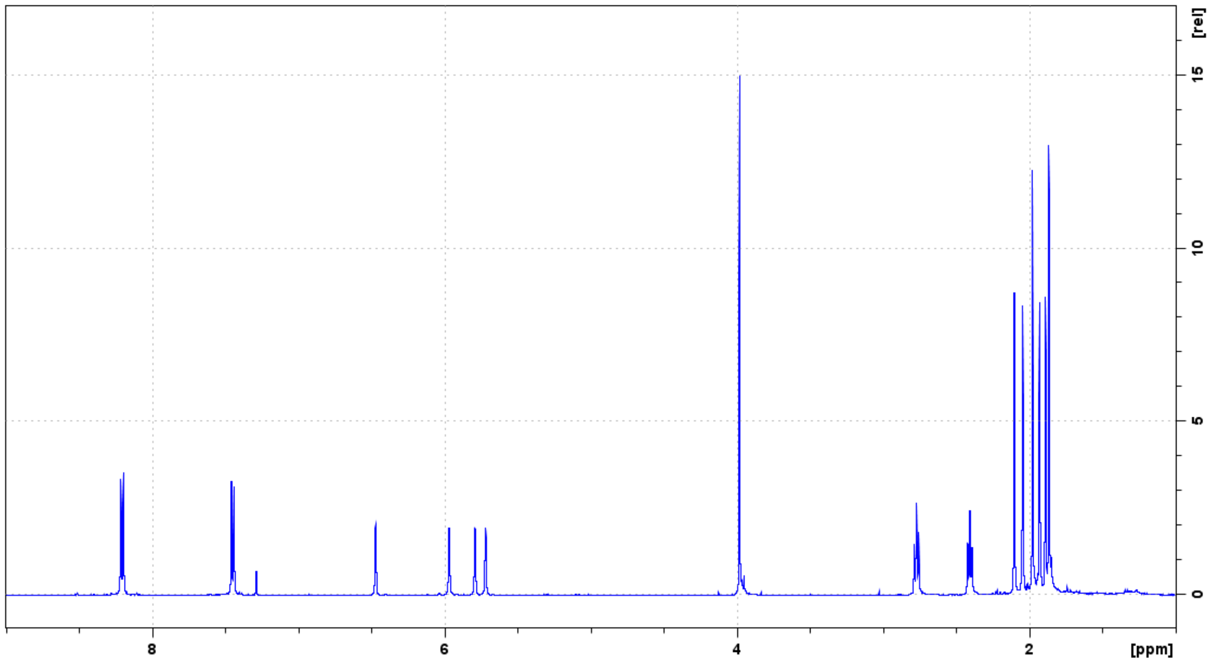

Supplementary Figure 25. ^{1}H NMR spectrum of deoxyneoaureothin. Compound **6.** Solvent $CDCl_3$.

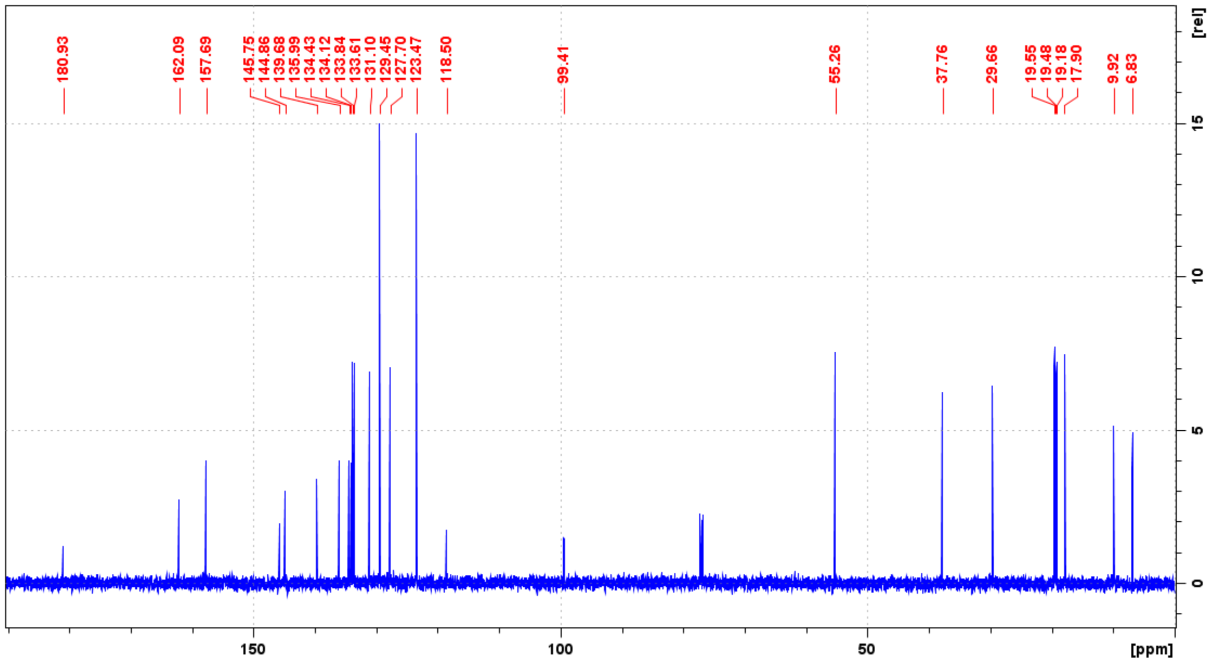

Supplementary Figure 26. ^{13}C NMR spectrum of deoxyneoaureothin. Compound **6.** Solvent $CDCl_3$.

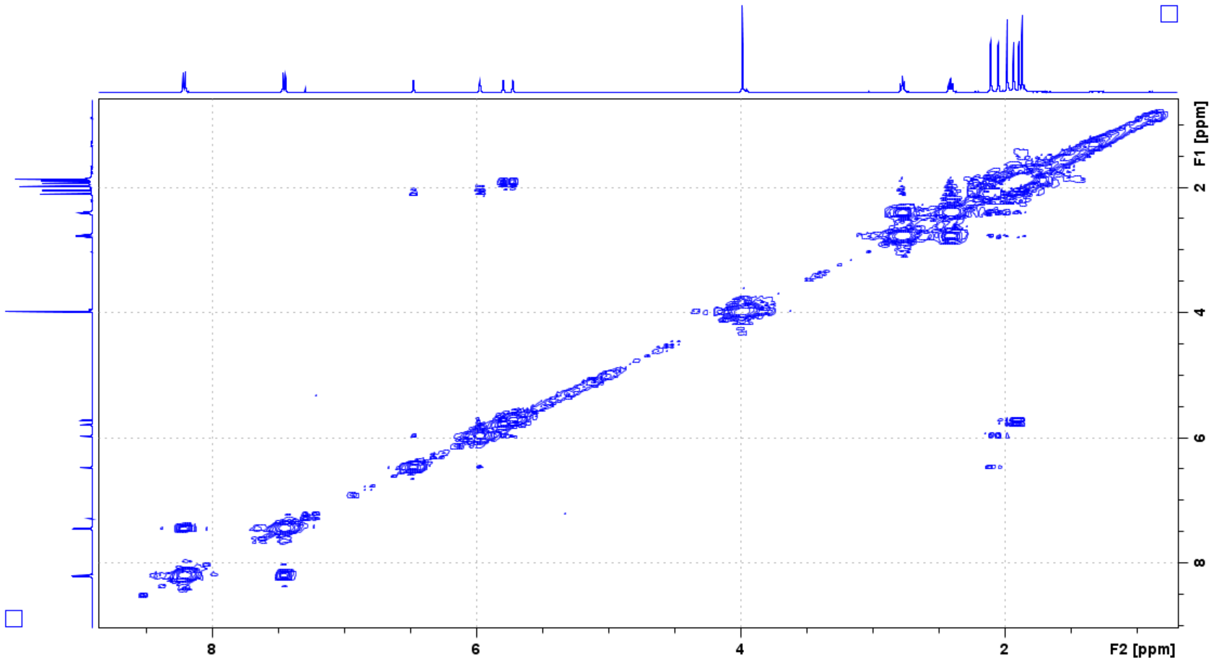

Supplementary Figure 27. ^{1}H-^{1}H COSY spectrum of deoxyneoaureothin. Compound **6.** Solvent $CDCl_3$.

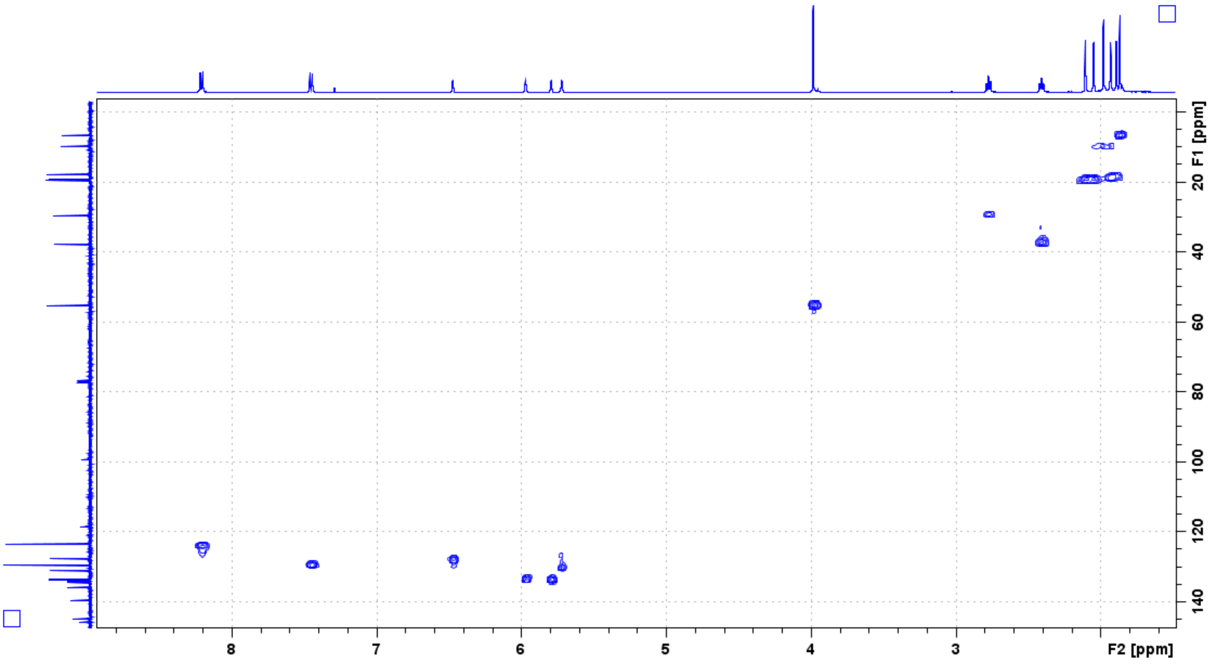

Supplementary Figure 28. HSQC spectrum of deoxyneoaureothin. Compound **6.** Solvent $CDCl_3$.

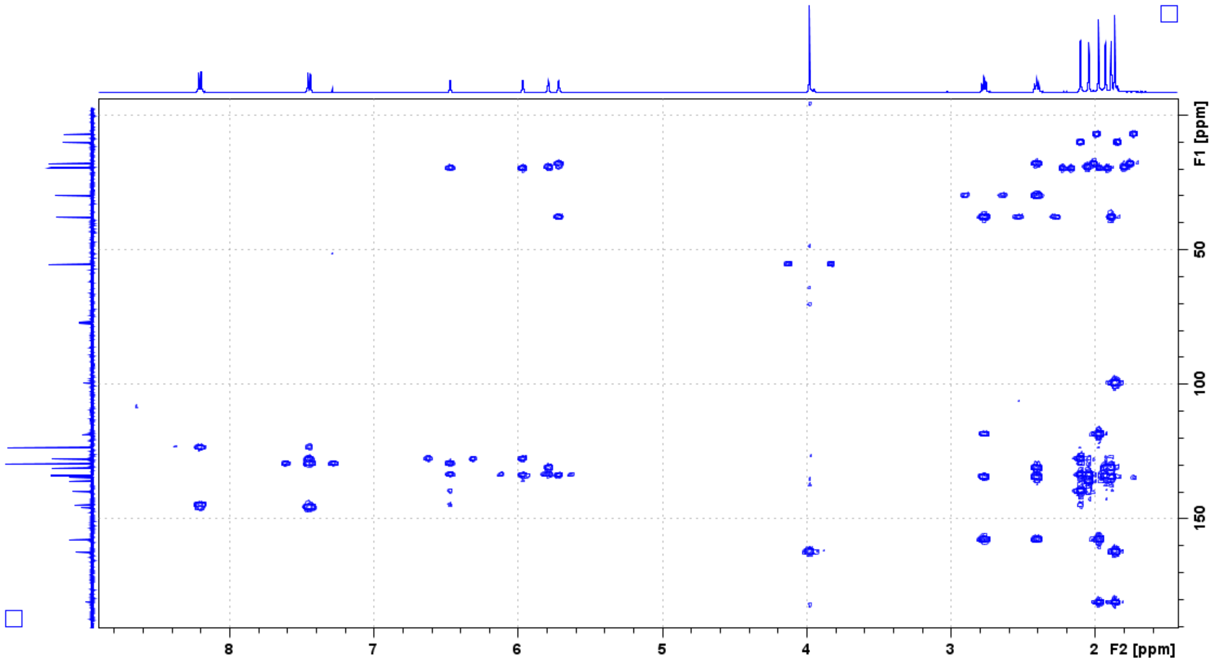

Supplementary Figure 29. HMBC spectrum of deoxyneoaureothin. Compound **6.** Solvent $CDCl_3$.

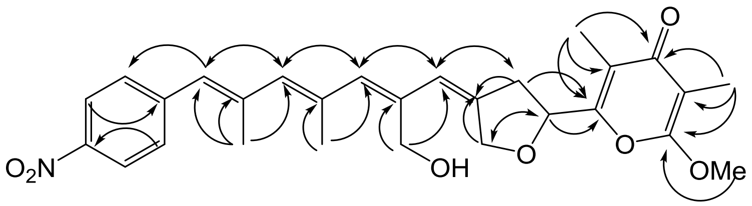

Supplementary Figure 30. Selected HMBC correlations of 11a-hydroxyneoaureothin. Compound **7**. HMBC correlations indicated by arrows.

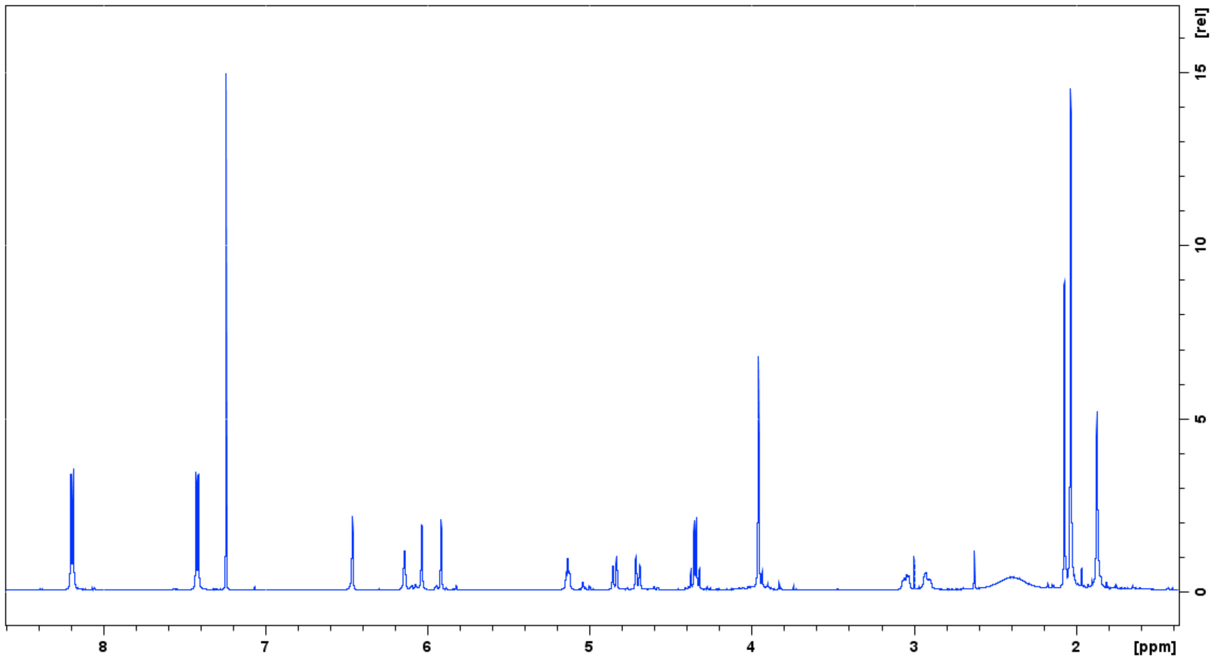

Supplementary Figure 31. ^{1}H NMR spectrum of 11a-hydroxyneoaureothin. Compound **7.** Solvent $CDCl_3$.

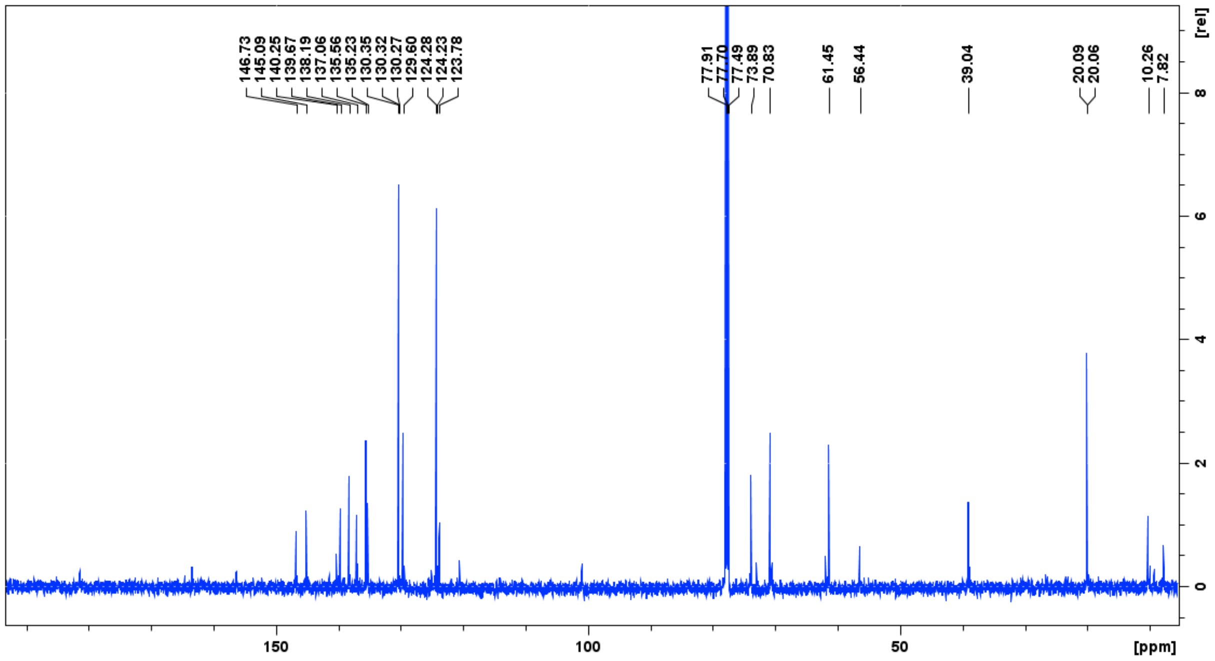

Supplementary Figure 32. ^{13}C NMR spectrum of 11a-hydroxyneoaureothin. Compound **7.** Solvent $CDCl_3$.

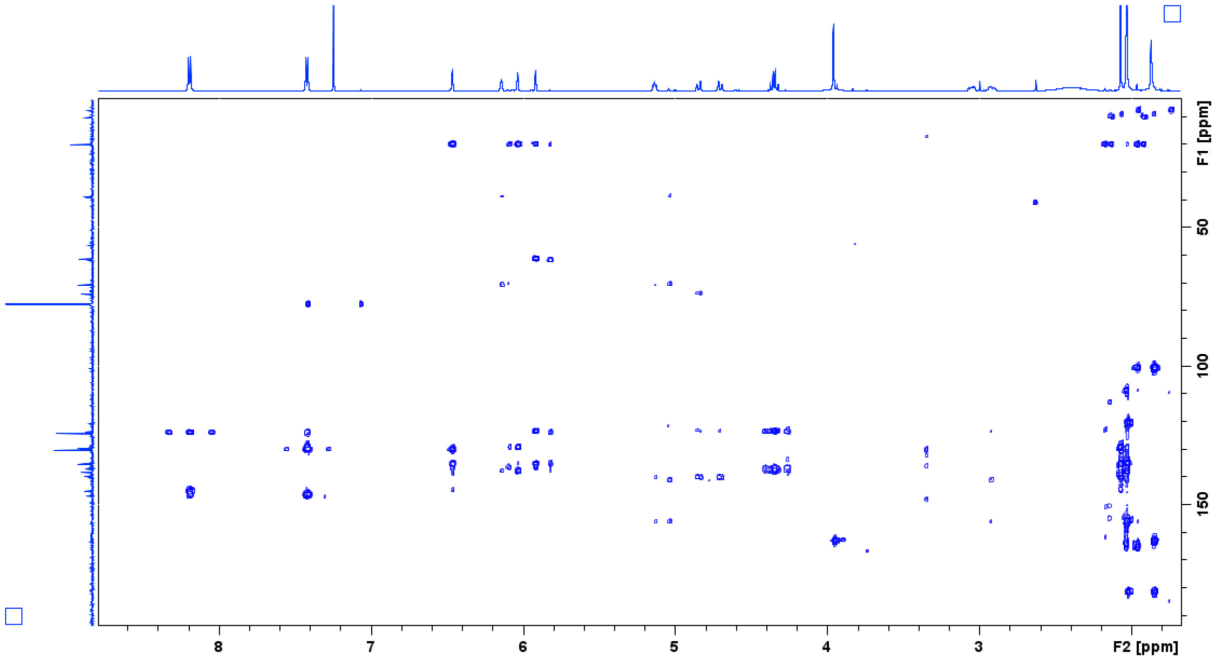

Supplementary Figure 33. HMBC spectrum of 11a-hydroxyneoaureothin. Compound **7.** Solvent $CDCl_3$.

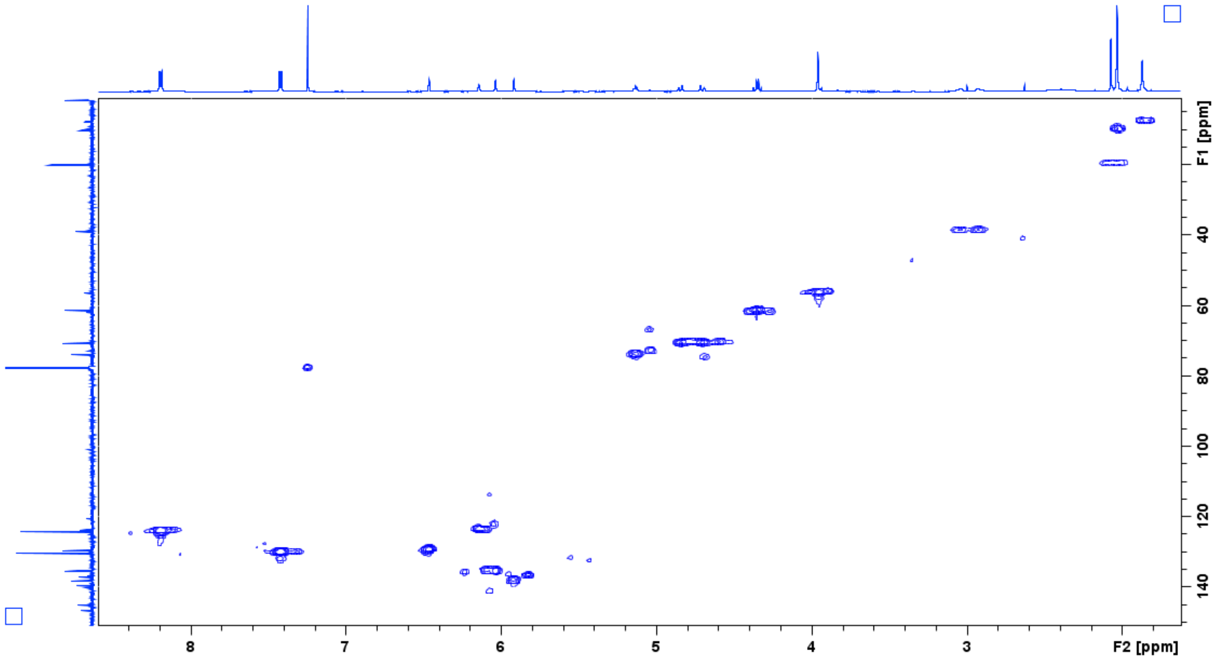

Supplementary Figure 34. HSQC spectrum of 11a-hydroxyneoaureothin. Compound **7.** Solvent $CDCl_3$.

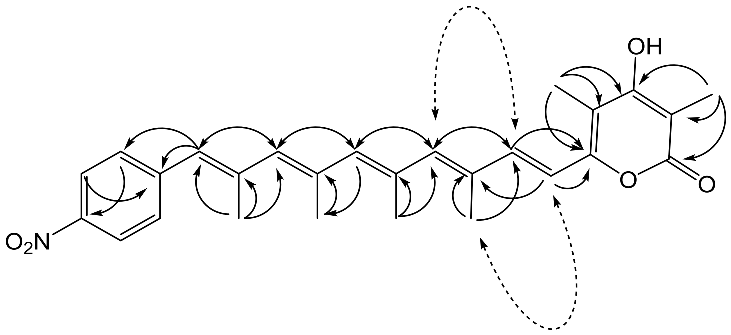

Supplementary Figure 35. Structure and selected 2D NMR correlations of 2-pyrone-4a-desmethyl-7-dehydro-7-deoxyneoaureothin. Compound **8.** HMBC correlations are indicated by arrows, ROESY correlations by dashed arrows.

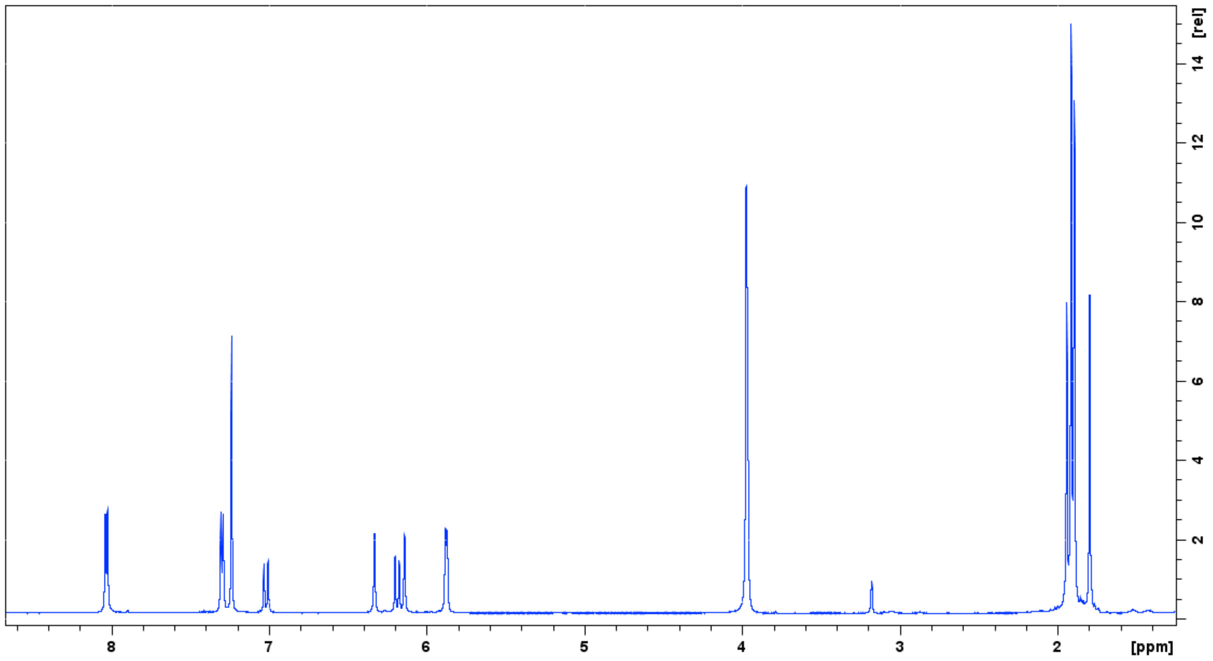

Supplementary Figure 36. ^{1}H NMR spectrum of 2-pyrone-4a-desmethyl-7-dehydro-7-deoxyneoaureothin. Compound **8.** Solvent $CDCl_3/CD_3OD$.

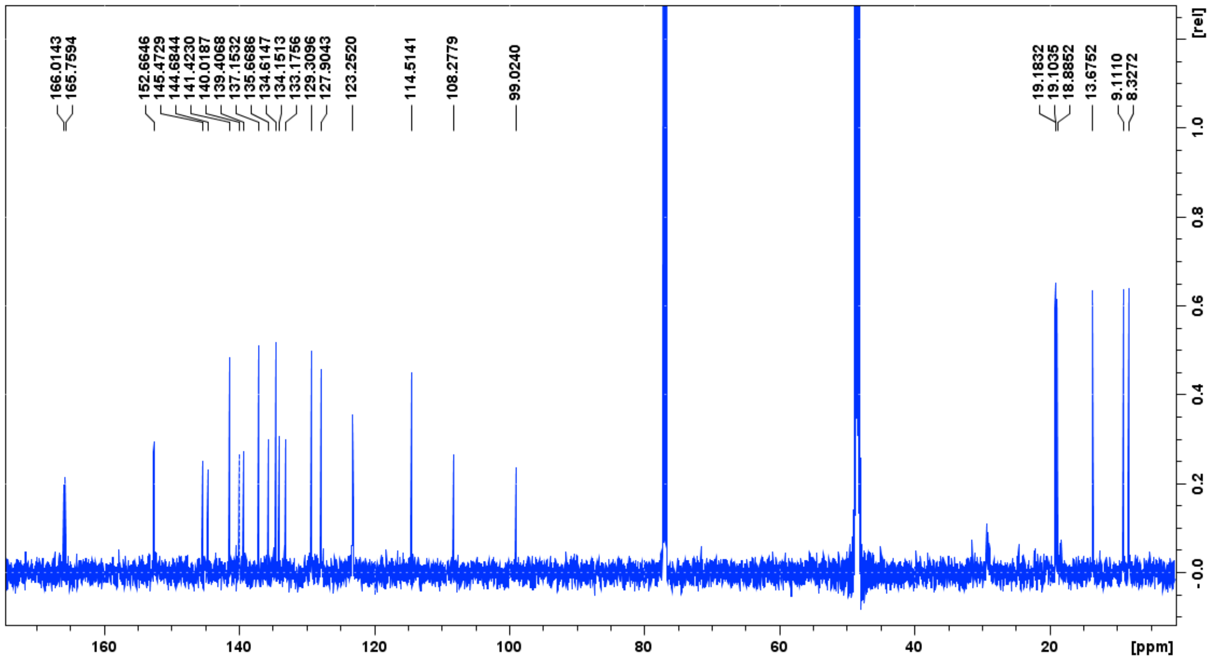

Supplementary Figure 37. ^{13}C NMR spectrum of 2-pyrone-4a-desmethyl-7-dehydro-7-deoxyneoaureothin. Compound **8.** Solvent $CDCl_3/CD_3OD$.

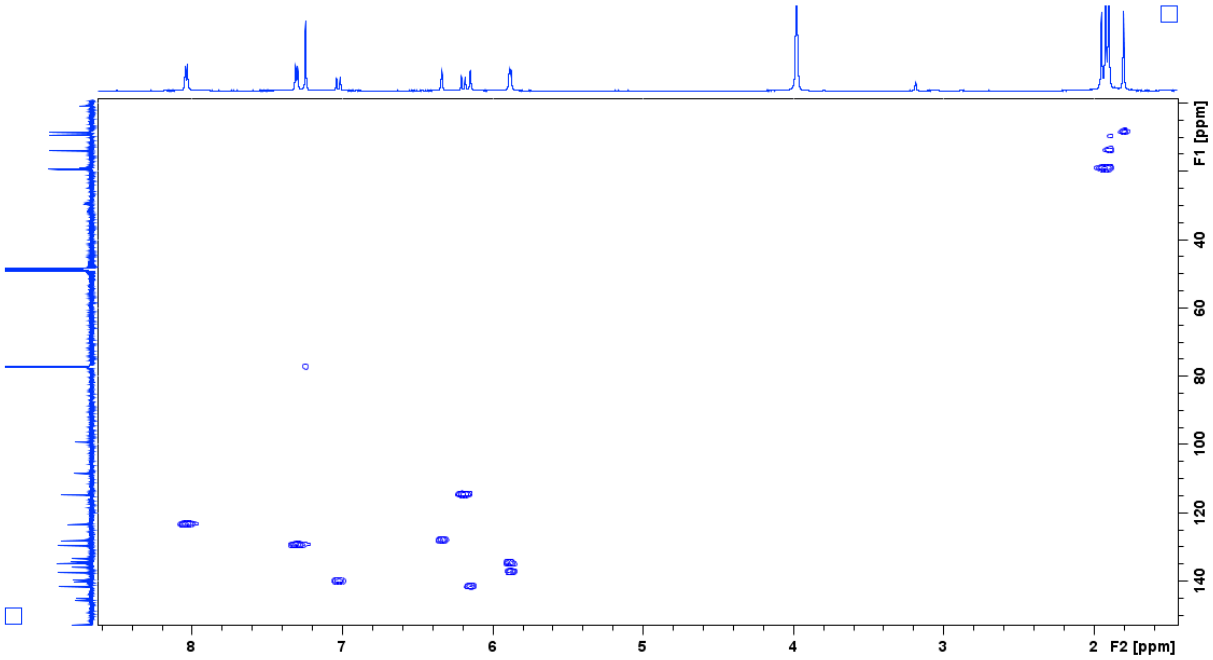

Supplementary Figure 38. HSQC spectrum of 2-pyrone-4a-desmethyl-7-dehydro-7-deoxyneoaureothin. Compound **8.** Solvent $CDCl_3$/CD_3OD.

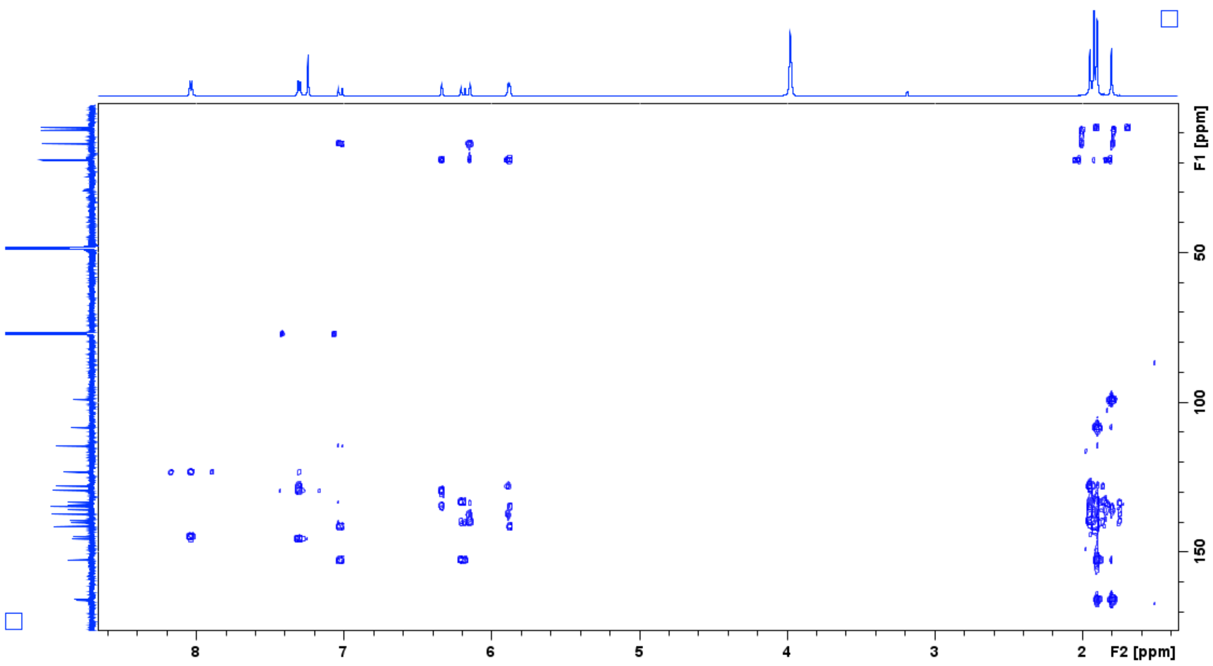

Supplementary Figure 39. HMBC spectrum of 2-pyrone-4a-desmethyl-7-dehydro-7-deoxyneoaureothin. Compound **8.** Solvent $CDCl_3/CD_3OD$.

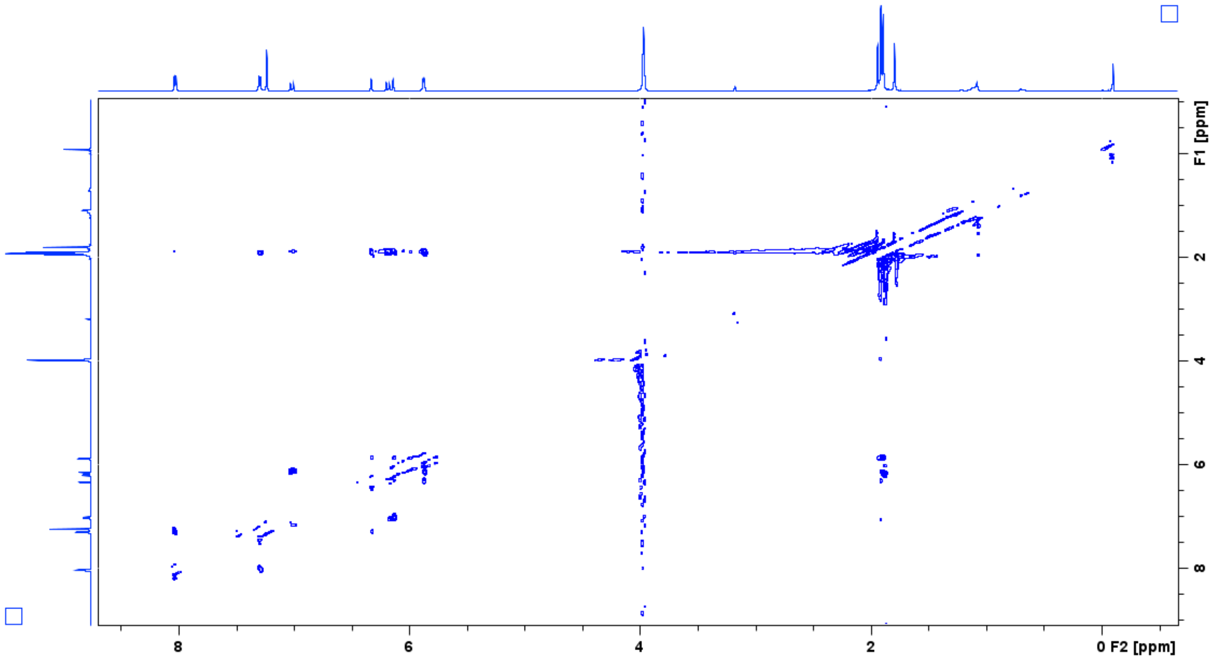

Supplementary Figure 40. ROESY spectrum of 2-pyrone-4a-desmethyl-7-dehydro-7-deoxyneoaureothin. Compound **8.** Solvent $CDCl_3/CD_3OD$.

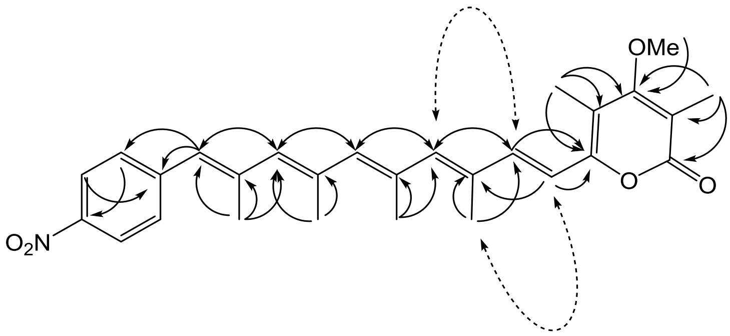

Supplementary Figure 41. Structure and selected 2D NMR correlations of 2-pyrone-7-dehydro-7-depxyneoaureothin. Compound **9.** HMBC correlations are indicated by arrows, ROESY correlations by dashed arrows.

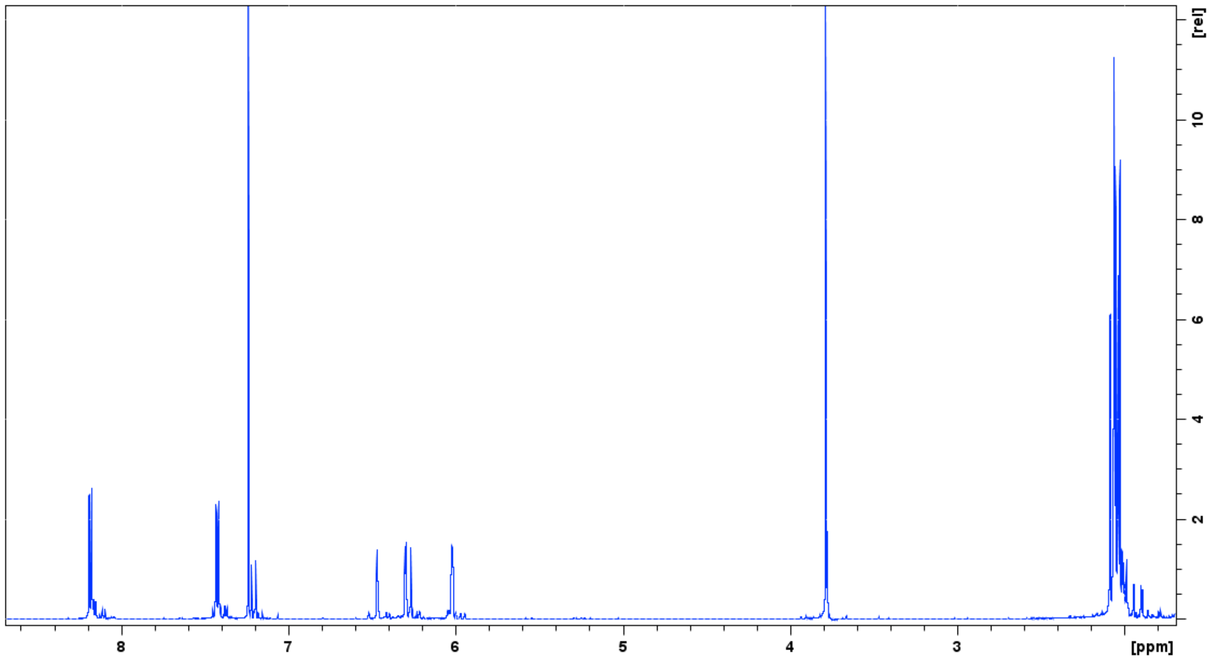

Supplementary Figure 42. ^{1}H NMR spectrum of 2-pyrone-7-dehydro-7-deoxyneoaureothin. Compound **9.** Solvent $CDCl_3$.

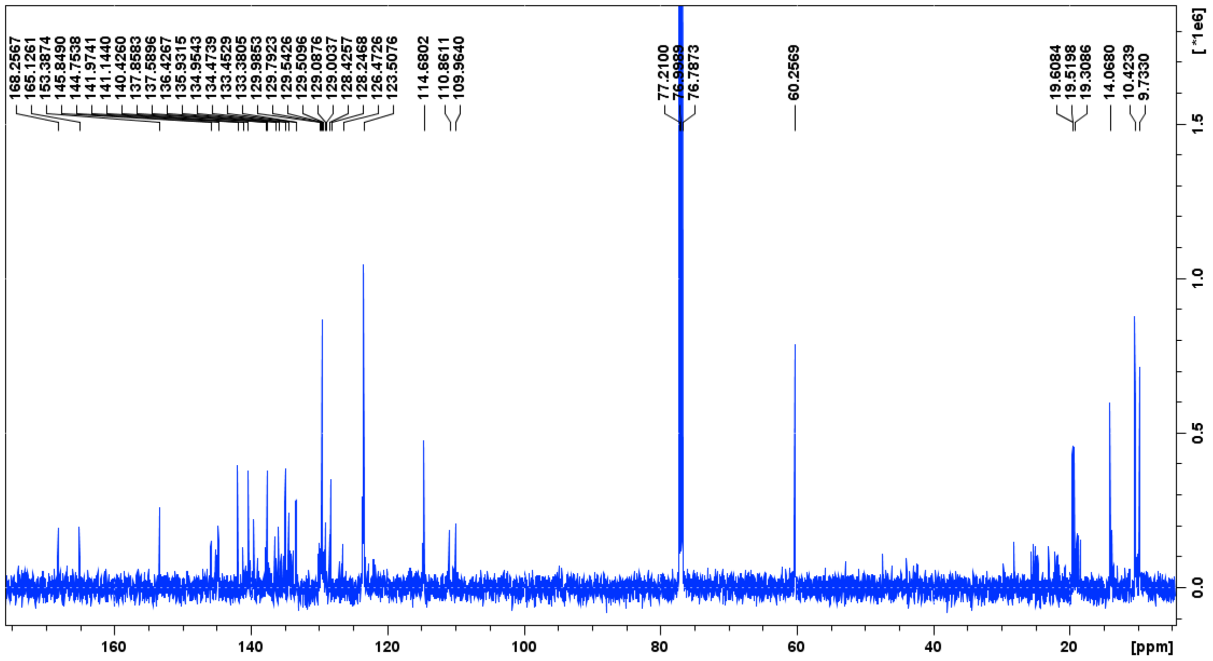

Supplementary Figure 43. ^{13}C NMR spectrum of 2-pyrone-7-dehydro-7-deoxyneoaureothin. Compound **9.** Solvent $CDCl_3$.

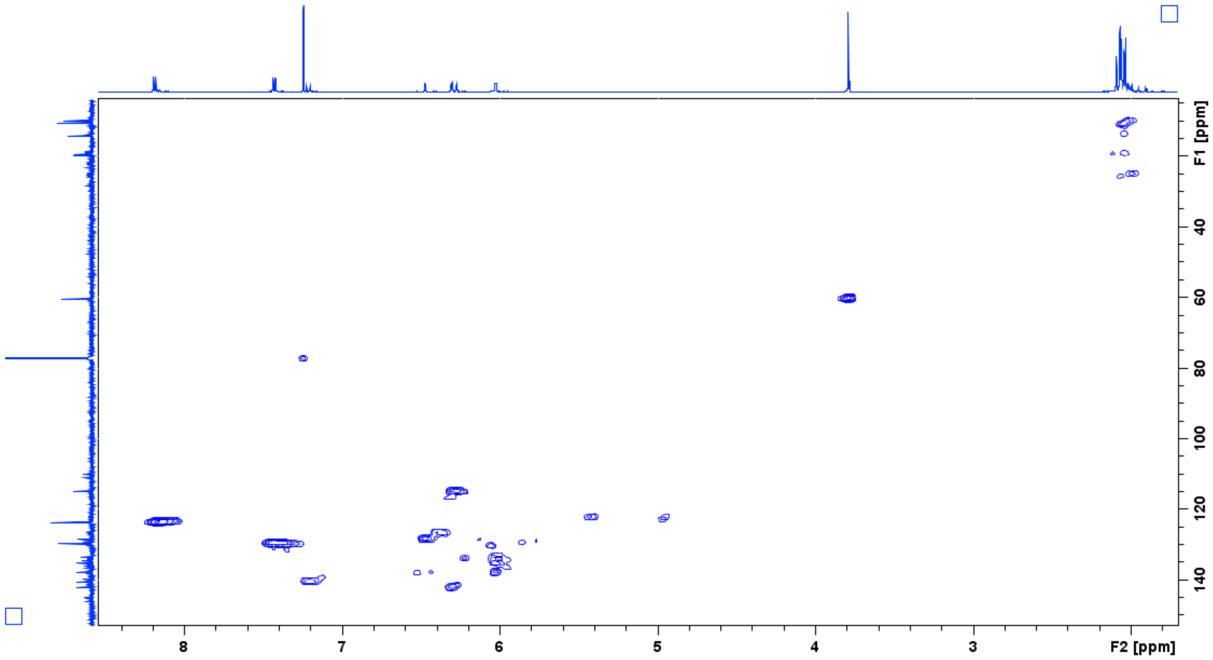

Supplementary Figure 44. HSQC spectrum of 2-pyrone-7-dehydro-7-deoxyneoaureothin. Compound **9.** Solvent $CDCl_3$.

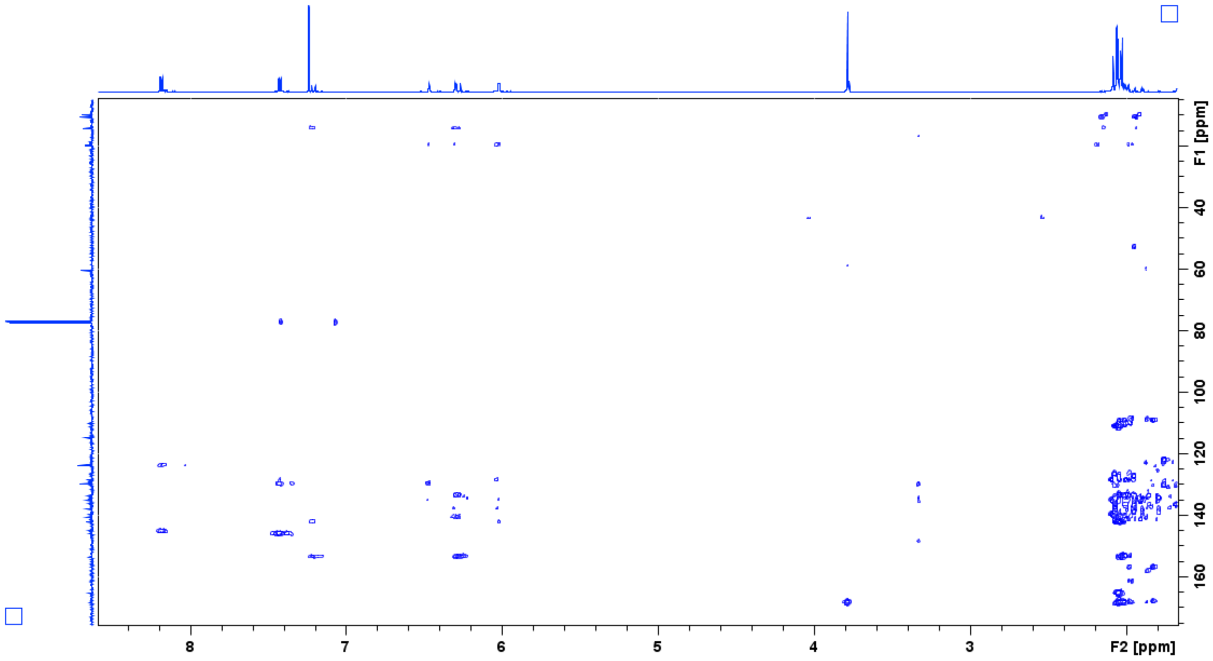

Supplementary Figure 45. HMBC spectrum of 2-pyrone-7-dehydro-7-deoxyneoaureothin. Compound **9.** Solvent $CDCl_3$.

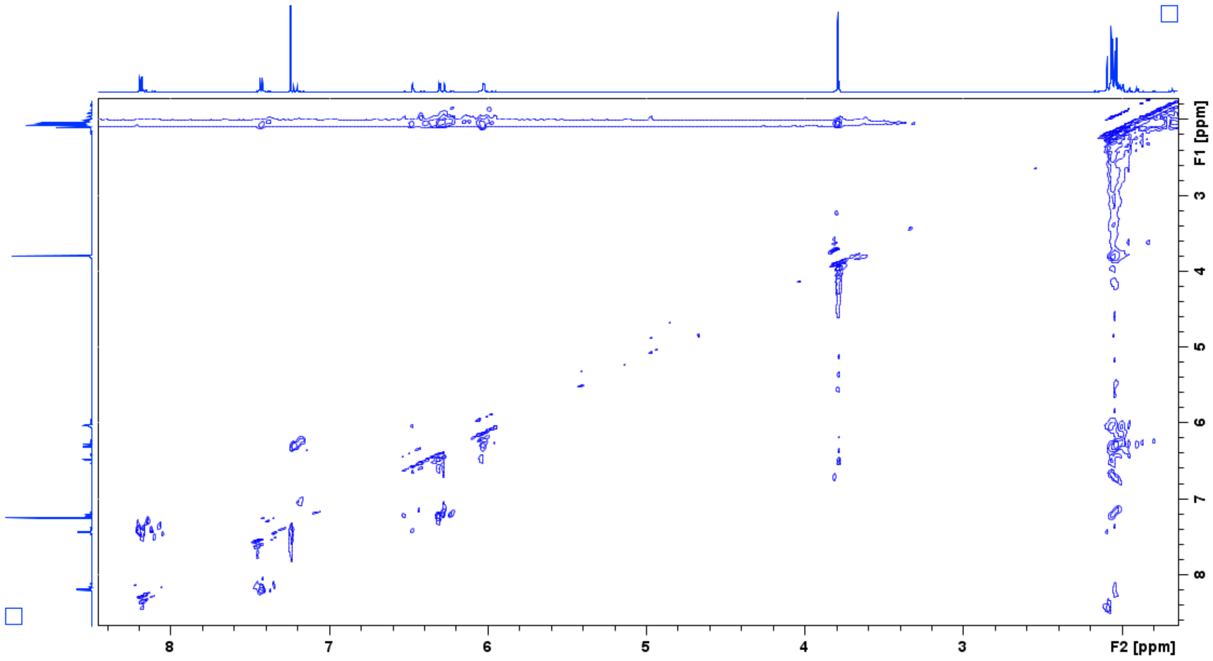

Supplementary Figure 46. ROESY spectrum of 2-pyrone-7-dehydro-7-deoxyneoaureothin. Compound **9.** Solvent $CDCl_3$.

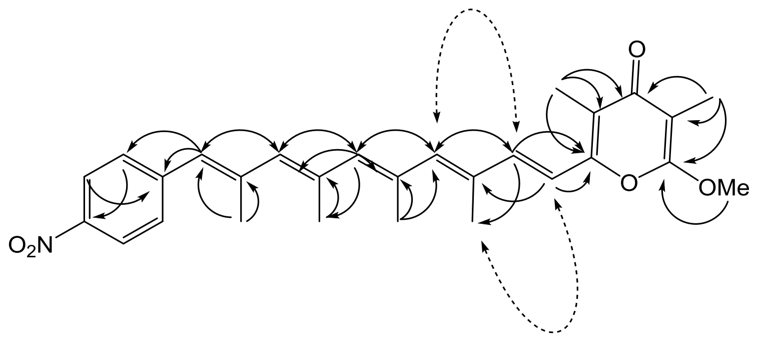

Supplementary Figure 47. Structure and selected 2D NMR correlations. Compound **10.** HMBC correlations are indicated by arrows, ROESY correlations by dashed arrows.

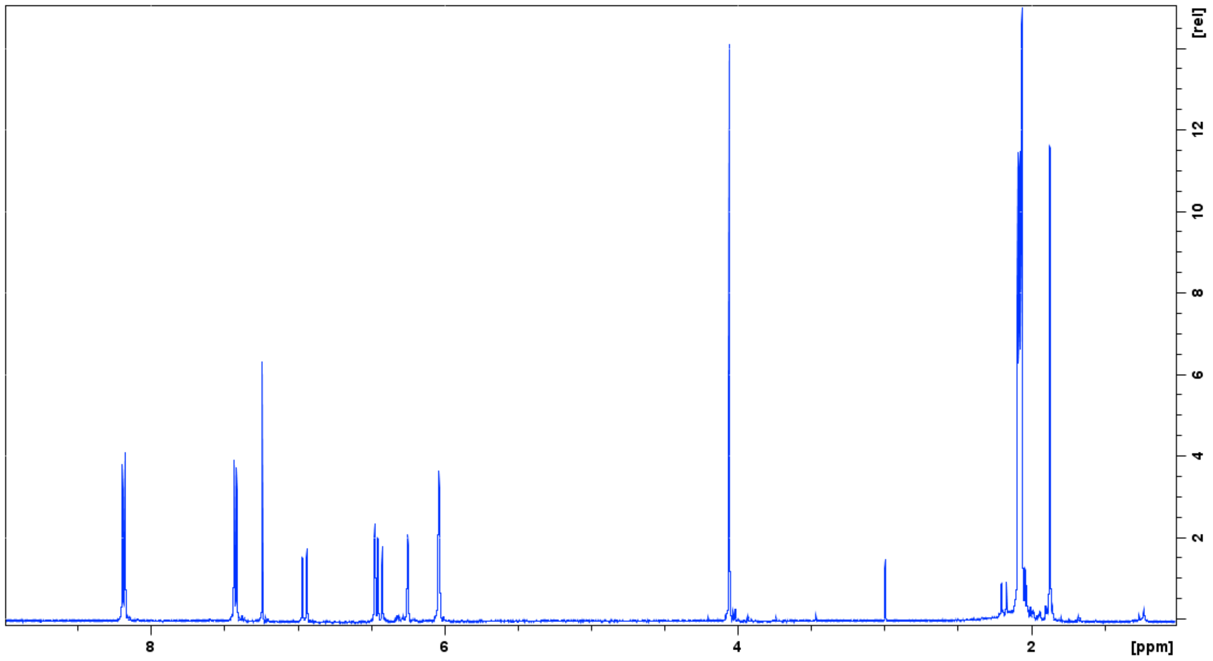

Supplementary Figure 48. ^{1}H NMR spectrum of 7-dehydro-7-deoxyneoaureothin. Compound **10**. Solvent $CDCl_3$.

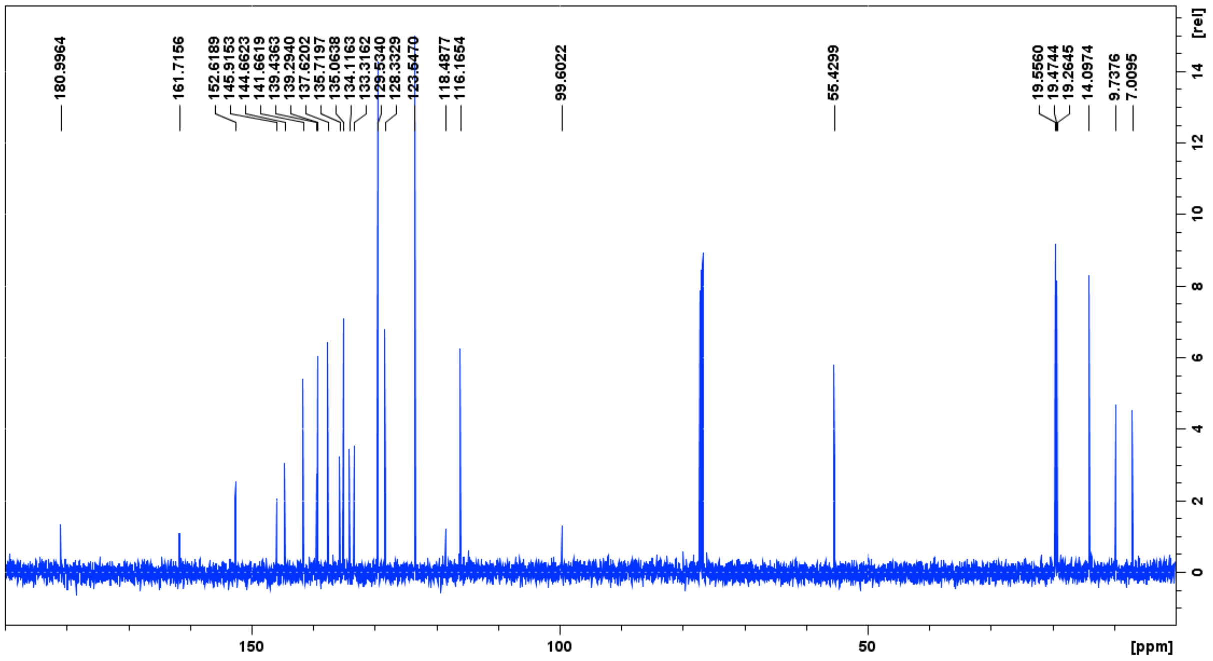

Supplementary Figure 49. ^{13}C NMR spectrum of 7-dehydro-7-deoxyneoaureothin. Compound **10**. Solvent $CDCl_3$.

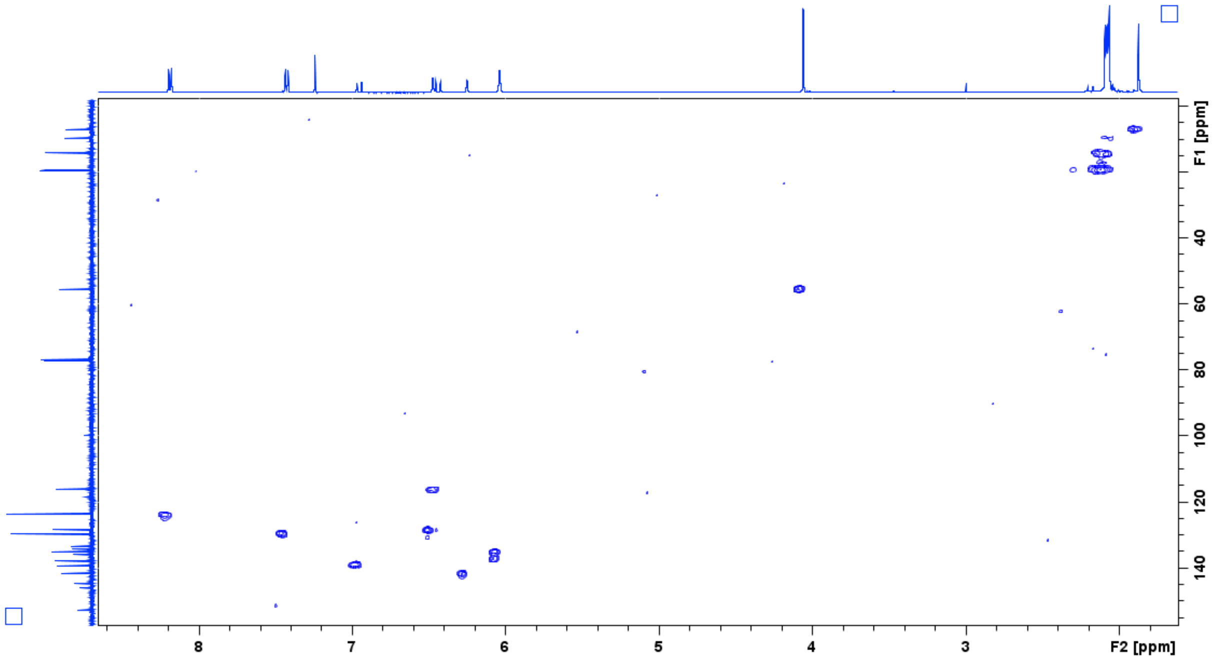

Supplementary Figure 50. HSQC spectrum of 7-dehydro-7-deoxyneoaureothin.
Compound **10**. Solvent $CDCl_3$.

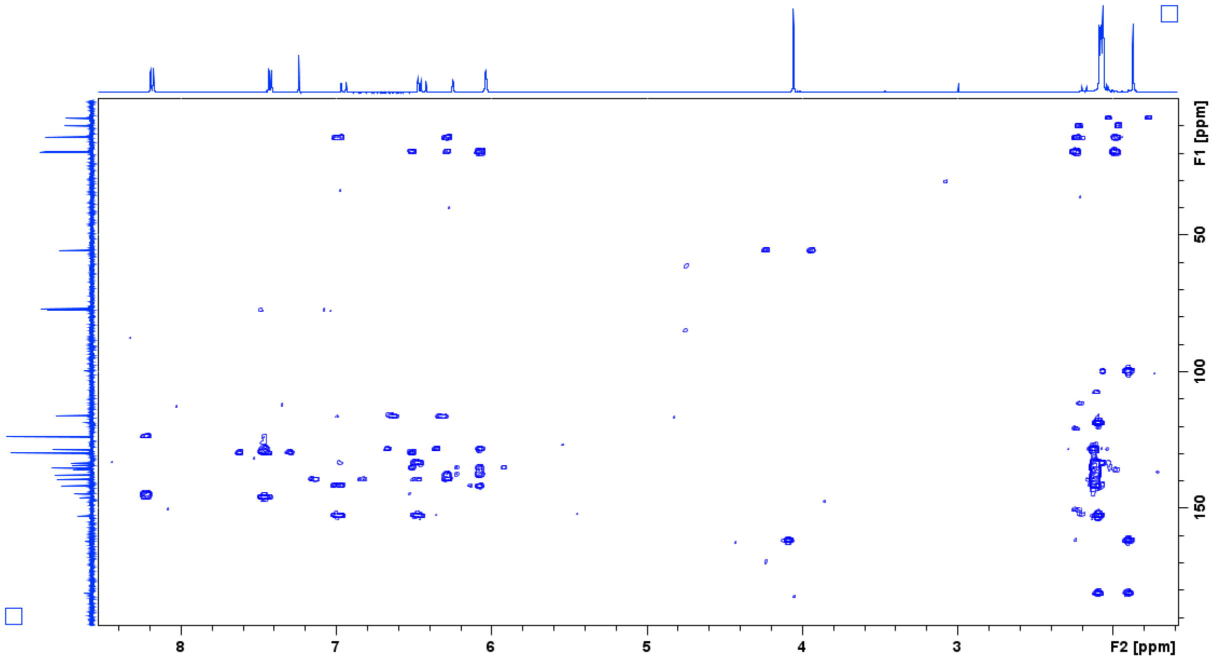

Supplementary Figure 51. HMBC spectrum of 7-dehydro-7-deoxyneoaureothin. Compound **10**. Solvent $CDCl_3$.

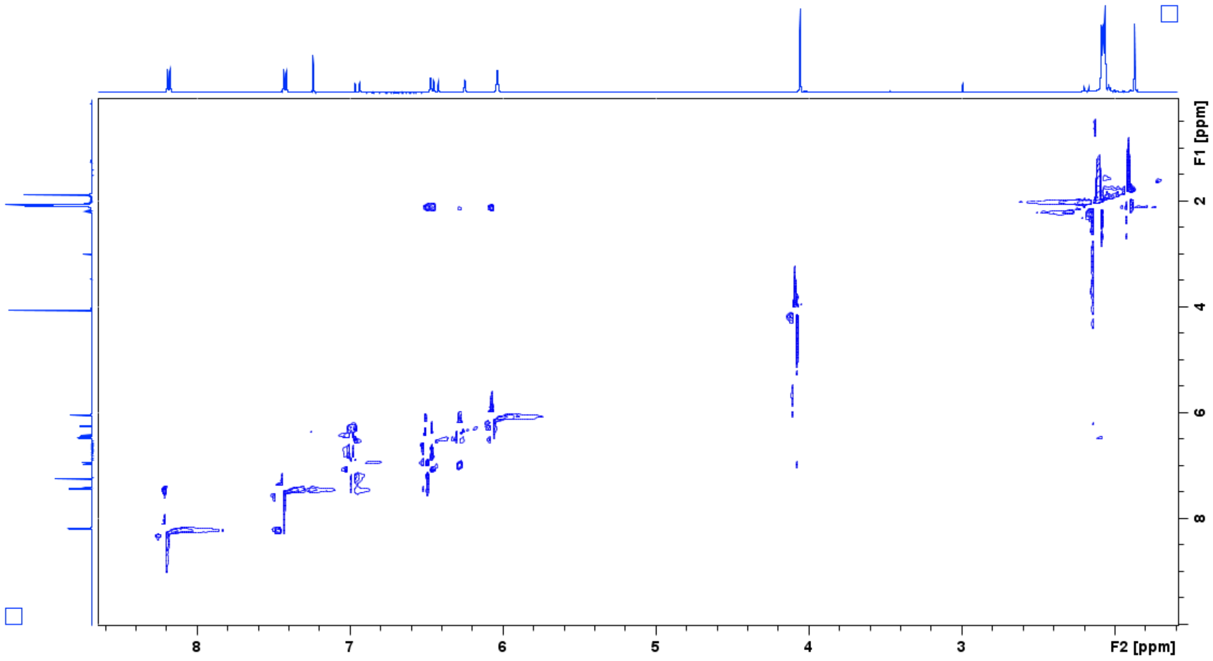

Supplementary Figure 52. ROESY spectrum of 7-dehydro-7-deoxyneoaureothin. Compound **10**. Solvent $CDCl_3$.

Supplementary Table 1. The *aur*- and *nor*-type gene clusters.

	S. thioluteus	***S. caatingaensis***	***S. scabrisoporus* DSM41855**	***S. orinoci***	***S. alboflavus* MDJK44**	***S. alboflavus* B-2373**	***S. ruber* NRRL ISP-5379 B-1661 ISP-5378**	***S.* sp. NRRL B-1348 B-1347**
**Aur/NorD**	CAE02599.1	WP_078871048.1	WP_079027019.1	CAO85889.1	WP_087886696.1	WP_078619364.1	WP_078619364.1	WP_078869010.2
**Aur/NorE**	CAE02600.1	WP_049715219.1	WP_020555942.1	CAO85890.1	WP_087886697.1	WP_030357148.1	WP_030357148.1	WP_018541096.2
**Aur/NorF**	CAE02601.1	WP_049715218.1	WP_020555941.1	CAO85891.1	WP_087886698.1	WP_051811201.1	WP_051811201.1	WP_030681451.2
**Aur/NorJ**	**nf**	**nf**	WP_020555940.1	CAO85892.1	WP_087886699.1	WP_051811200.1	WP_051811200.1	WP_078869021.2
**Aur/NorA**	CAE02602.1	KNB53282	WP_079027018.1	CAO85893.1	WP_087886700.1	WP_051811199.1	WP_051811199.1	WP_051855294
**Aur/NorG**	CAE02603.1	WP_049715217.1	WP_020555938.1	CAO85894.1	WP_030357152.1	WP_030357152.1	WP_030357152.1	WP_030681455.2
**Aur/NorK**	**nf**	**nf**	**nf**	**nf**	WP_087886701.1	WP_030357153.1	WP_030357153.1	WP_030681457.2
**Aur/NorH**	CAE02604.1	WP_049715216.1	WP_063744948.1	CAO85895.1	WP_087886702.1	WP_078619363.1	WP_078619363.1	WP_078869011.2
**NorA'**	**nf**	**nf**	WP_020555936.1	CAO85896.1	WP_087886706.1	WP_051811198.1	WP_065914235	WP_051855297.2
**Aur/NorB**	CAE02605.1	WP_049715215.1	**nf**	CAO85897.1	WP_087886707.1	WP_051811197.1	WP_051811197.1	WP_030681465
**Aur/NorC**	CAE02606.1	WP_053161065.1	WP_052174042.1	CAO85898.1	ARX88036.1	**Gap**	**Gap**	WP_037826258
**Aur/NorI**	CAE02607.1	WP_049715214.1	WP_020555935.1	CAO85899.1	WP_087887989.1	WP_030357158.1	WP_030357158.1	WP_030681469.2

Supplementary Table 2. Proteins used for phylogenies.

Polyketide	Protein	Organism	Accession number
Avermectin[21]	AveA1	*Streptomyces avermitilis*	Q9S0R8
	AveA2		Q9S0R7
	AveA3		Q9S0R4
	AveA4		Q9S0R3
Polyene macrolide[22]	PteA1		Q93H87
	PteA2		Q93H86
	PteA3		Q93H85
	PteA4		Q93H84
	PteA5		Q93H83
Oligomycin[22]	OlmA1		Q93HJ5
	OlmA2		Q93HJ4
	OlmA3		Q93HJ3
	OlmA4		Q93HI8
	OlmA5		Q93HI9
	OlmA6		Q93HJ2
	OlmA7		Q93HJ1
Oleandomycin[23]	OleAI	*Streptomyces antibioticus*	Q9KIV4
	OleAII		Q9KIV3
Niddamycin[24]	NidA1	*Streptomyces caelestis*	O30764
	NidA2		O30765
	NidA3		O30766
	NidA4		O30767
	NidA5		O30768
Monensin[13]	MonAI	*Streptomyces cinnamonensis*	Q846X6
	MonAII		Q846X5
	MonAIII		Q846X4
	MonAIV		Q846X3
	MonAV		Q846X2
	MonAVI		Q864X1
	MonAVII		Q846W5
	MonAVIII		Q846W6
Tylactone[15]	TylG1	*Streptomyces fradiae*	O33954
	TylG2		O33955
	TylG3		O33956
	TylG4		O33957
	TylG5		O33958
FK520[25]	FkbA	*Streptomyces hygroscopicus*	P95814
	FkbB	subsp. *ascomyceticus*	Q9ZGA4
	FkbC		Q9KIE1
Rapamycin[26]	RapA	*Streptomyces hygroscopicus*	Q54297
	RapB		Q54296
	RapC		Q54299
Nanchangmycin[27]	NanA1	*Streptomyces nanchangensis*	Q7WTF5
	NanA2		Q7WTF4
	NanA3		Q7WTF3
	NanA4		Q7WTF2
	NanA5		Q7WTF1
	NanA6		Q7WTF0
	NanA7		Q7WTE3
	NanA8		Q7WTD6
	NanA11		Q7WTD7
Pimaricin[16]	PimS0	*Streptomyces natalensis*	Q9X992
	PimS1		Q9X993

	PimS2		Q9EWA1
	PimS3		Q9EWA2
	PimS4		Q9EWA3
Amphotericin[28]	AmphA	*Streptomyces nodosus*	Q93NW8
	AmphB		Q93NW7
	AmphC		Q93NW6
	AmphI		Q93NX9
	AmphJ		Q93NX8
	AmphK		Q93NX7
Nystatin[12]	NysA	*Streptomyces noursei*	Q9L4W5
	NysB		Q9L4W4
	NysC		Q9L4W3
	NysI		Q9L4X3
	NysJ		Q9L4X2
	NysK		Q9L4X1
Candicidin[29]	FscA	*Streptomyces* str. FR-008	Q9W5Q3
	FscB		Q6W5P9
	FscC		Q6W5Q0
	FscD		Q6W5P6
	FscE		Q6W5P7
	FscF		Q6W5P8
Pikromycin[9]	PikAI	*Streptomyces venezuelae*	Q9ZGI5
	PikAII		Q9ZGI4
	PikAIII		Q9ZGI3
	PikAIV		Q9ZGI2
Erythromycin[8]	DEBS1	*Saccharopolyspora erythraea*	Q5UNP6
	DEBS2		Q5UNP5
	DEBS3		Q5UNP4
Spinosad[14]	SpnA	*Saccharopolyspora spinosa*	Q9ALM6
	SpnB		Q9ALM5
	SpnC		Q9ALM4
	SpnD		Q9ALM3
	SpnE		Q9ALM2
Rifamycin[17]	RifA	*Amycolatopsis mediterranei*	O54666
	RifB		O52545
	RifC		O52790
	RifD		O54591
	RifE		O54593
Mycinamicin[30]	MycAI	*Micromonospora griseorubida*	Q83WF0
	MycAII		Q83WE9
	MycAIII		Q83WE8
	MycAIV		Q83WE7
	MycAV		Q83WE6
Megalomicin[31]	MegAI	*Micromonospora megalomicea*	Q9F830
	MegAII		Q9F829
	MegAIII		Q9F828

Supplementary Table 3. List of primers for site-directed mutagenesis of *norH*.

Primers	Sequence (from 5' to 3', mutated sites are underlined)
I19F-fw	CTGGACTTCATCTCGCCCGGCTTCGCCTGGGACTC
I19F-rv	GCCGGGCGAGATGAAGTCCAGGAACGGCAGG
V71L-fw	CGCGATCTGGTGGACCTGGTCGGGCCGCC
V71L-rv	GACCAGGTCCACCAGATCGCGGAAGCCGGAGA
T291L-fw	CCCACCCTCACCGTGGTGGCCACCAAGTCCG
T291L-rv	GGCCACCACGGTGAGGGTGGGGGCCCAG
T292P-fw	ACCACCCCCGTGGTGGCCACCAAGTCCGCC
T292P-rv	GGTGGCCACCACGGGGGTGGTGGGGGC
W317F-fw	CCGGTCTTTCTGTGCGCCCACTCCGCCCAGC
W317F-rv	GTGGGCGCACAGAAAGACCGGGGTGCCGG
T392L-fw	CCGGAGCTCGGCGTCTCCGGCCCGGAC
T392L-rv	GCCGGAGACGCCGAGCTCCGGGCGCCAG

Supplementary Table 4. Identities/positives of AurH and homologous amino acid sequences.

	AurH-*S. thioluteus*	AurH-*S. caatingaensis*	NorH-*S. scabrisporus*	NorH-*S. orinoci*	NorH-*S. alboflavus* MDJK44	NorH-*S. ruber* 5378	NorH-*S.* sp. B-1347
AurH-*S. thioluteus*	100/100	93/96	63/74	64/74	63/75	64/75	62/74
AurH-*S. caatingaensis*	93/96	100/100	65/75	64/75	64/75	64/75	62/75
NorH-*S. scabrisporus*	63/74	65/75	100/100	76/83	89/92	89/93	79/87
NorH-*S. orinoci*	64/74	64/75	76/83	100/100	79/85	79/84	77/85
NorH-*S. alboflavus* MDJK44	63/75	64/75	89/92	79/85	100/100	99/99	78/85
NorH-*S. ruber* 5378	64/75	64/75	89/93	79/84	99/99	100/100	78/85
NorH-*S.* sp. B-1347	62/74	62/75	79/87	77/85	78/85	78/85	100/100

The identities and positives comparison of AurH, NorH and homologues encoded in genomes of *Streptomyces* species was constructed by protein BLAST in NCBI. As shown in Supplementary Table 4, AurH from *S. thioluteus* is highly similar to AurH from *S. caatingaensis*: 93% identities and 96% positives. The NorH homologues show a high similarity with each other. For example, NorH-*S. alboflavus* MDJK44 and NorH-*S. ruber* 5378 show 99% identities and 99% positives. The positives between AurH and NorH range from 74% to 75%, while the positives between NorH homologues range from 83% to 99%. This indicates that NorH homologous are more similar to each other than to AurH.

Supplementary Table 5. List of primers for region-swapped chimeric *norH* variants.

Primers	Sequence (from 5' to 3', mutated sites are underlined)
AurH-R1-fw	GACCAACTGGCCCGCGACAAGCGGCTGATCTCCGGCTTCCGCGATGTGGT
AurH-R1-rv	GATCAGCCGCTTGTCGCGGGCCAGTTGGTCTGCCTCGGCGTACCTCAGCA
AurH-R2-fw	GGCCTGGTCGACATGGTCGGGACCCCGGAGGGCATCGTCCGCGACTTTAT
AurH-R2-rv	GCCCTCCGGGGTCCCGACCATGTCGACCAGGCCGCGGAAGCCGGAGACCA
AurH-R3-fw	CGGCTGTCCATCGAGACCAACCTCGGCCTGGCGCTCGGCGACCTCAGCCT
AurH-R3-rv	CAGGCCGAGGTTGGTCTCGATGGACAGCCGGCCGACGGTGGCCTGGTCCT
AurH-R4-fw	TCCAACGACCAGGACATCCTCGTCAAGGTGGAGGAGGCGCTGAGCGG
AurH-R4-rv	CTTGACGAGGATGTCCTGGTCGTTGGAGAGCGCCGCCACCAGGTTGG
AurH-R5-fw	CACGAGCTCGGCGTCGCCGGGCCGGACGCCCTGCCACTGCGCTTCGGCGC
AurH-R5-rv	GGCGTCCGGCCCGGCGACGCCGAGCTCGTGGCGCCAGCTGATCTCCCCGG

Supplementary Table 6. List of primers for hybrid *norH/aurH* variants.

Primers	Sequence (from 5' to 3')
N-NorH-fw	GGGCTGCAGGAATTCAACTAGTGATTATCACCATGCGGTG
N-NorH-rv	CAGGGTGCGCAGTTCGTCCTCGTCCAGC
C-AurH-fw	CGAACTGCGCACCCTGGTCGCCACCGTCC
C-AurH-rv	ATACTCTTCCTTTTTCAATTCAGGCCGCGCCGAAGCGCA
N-AurH-fw	GGGCTGCAGGAATTCAACTAGTTTCGGCGAACAGAATG
N-AurH-rv	CAGGGTGCGCAGCTCGTAGTCGTCGAGG
C-NorH-fw	CGAGCTGCGCACCCTGGTGGCCACCCTG
C-NorH-rv-1	ATACTCTTCCTTTTTCAATTCAGGCGCCGAAGCGCAGTG
NorH-A-rv	CCAGGCCGCGCAGCCTGCGGTGGTC
AurH-b-fw	GACCACCGCAGGCTGCGCGGCCTGG

Supplementary Table 7. Substrate recognition of AurH and NorH in relation to compound size.

	**Substrates and resulting structures**			
	deoxy-aureothin	deoxy-homoaureothin	deoxy-neoaureothin	deoxy-homoneoaureothin
	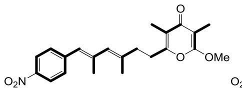	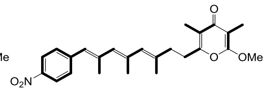	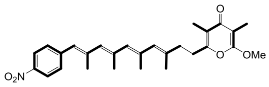	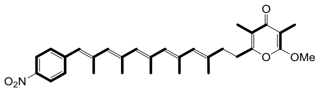
AurH	THF ring	-	THF ring	THF ring
NorH	C-7 oxidation	THF ring	THF ring	THF ring

THF: tetrahydrofuran, -: unknown

Supplementary Table 8. ^{1}H and ^{13}C NMR data for intermediate 5.

Position	^{1}H (J_{Hz})	^{13}C (mult)	HMBC correlations (H to C)
1		171.0 (s)	
2		128.7 (s)	
2a	1.95 (s)	14.3 (q)	C1, 2, 3
3	7.15 (s)	142.7 (d)	C1, 2, 2a, 4, 4a, 5
4		138.3 (d)	
4a	1.98 (s)	18.6 (q)	C4, 5, 6
5	6.49 (s)	131.7 (d)	C3, 4a, 6, 7, 11
6		144.0 (s)	
7,11	7.33 (d 8.7)	130.0 (s)	C5, 7, 8, 9, 10, 11,
8,10	8.08 (d 8.7)	123.7 (d)	C6, 7, 8, 9, 10, 11
9		146.5 (s)	

Supplementary Table 9. ^{1}H and ^{13}C NMR data for deoxyneoaureothin and compound 1.

Position	Deoxyneoaureothin (6)		Compound 1[#]	
	^{1}H (J_{Hz})	^{13}C (mult)	^{1}H (J_{Hz})	^{13}C (mult)
1				
2		162.1 (s)		162.1 (s)
2a	3.94 (s)	55.3 (q)	3.97 (s)	55.3 (q)
3		99.4 (s)		99.4 (s)
3a	1.82 (s)	6.8 (q)	1.85 (s)	6.9 (q)
4		180.9 (s)		181.0 (s)
5		118.5 (s)		118.5 (s)
5a	1.93 (s)	9.9 (q)	1.97 (s)	10.0 (q)
6		157.7 (s)		157.7 (s)
7	2.73 (t 7.5)	29.7 (t)	2.76 (dd 7.3, 7.5)	29.7 (t)
8	2.36 (t 7.5)	37.8 (t)	2.39 (dd 7.3, 7.5)	37.8 (t)
9		134.1 (s)		134.2 (s)
9a	1.84 (s)	17.9 (q)	1.88 (s)	17.9 (q)
10	5.67 (s)	131.1 (d)	5.70 (s)	131.1 (d)
11		134.4 (s)		134.5 (s)
11a	1.88 (s)	19.2 (q)	1.92 (s)	19.2 (q)
12	5.74 (s)	133.8 (d)	5.78 (s)	133.9 (d)
13		136.0 (s)		136.0 (s)
13a	2.00 (s)	19.6 (q)	2.03 (s)	19.6 (q)
14	5.92 (s)	133.6 (d)	5.95 (s)	133.6 (d)
15		139.7 (s)		139.7 (s)
15a	2.06 (s)	19.5 (q)	2.09 (s)	19.5 (q)
16	6.42 (s)	127.7 (d)	6.46 (s)	127.7 (d)
17		144.9 (s)		144.9 (s)
18,22	7.40 (d 8.8)	129.5 (d)	7.43 (d 8.8)	129.5 (d)
19,21	8.16 (d 8.8)	123.5 (d)	8.20 (d 8.8)	123.5 (d)
20		145.8 (s)		145.8 (s)

#: The NMR data of compound 1.[32]

Supplementary Table 10. ^{1}H and ^{13}C NMR data for 11a-hydroxyneoaureothin#.

Position	^{1}H (J_{Hz})	^{13}C (mult)	HMBC correlations (H to C)
1			
2		163.4 (s)	
2a	3.96 (s)	56.4 (q)	C2
3		100.9 (s)	
3a	1.78 (s)	7.8 (q)	C2, 3, 4
4		181.4 (s)	
5		120.6 (s)	
5a	2.03 (s)	10.3 (q)	C4, 5, 6
6		156.3 (s)	
7	5.13 (brt 6.1)	73.9 (d)	C6, 8, 9, 9a
8	2.91 (brdd 15.6, 5.6)	39.0 (t)	C6, 7, 9, 9a, 10
	3.05 (brss 15.6, 6.9)		
9		140.3 (s)	
9a	4.70 (d 14.0)	70.8 (t)	C7, 8, 9, 10
	4.84 (d 14.0)		
10	6.14 (s)	123.8 (d)	C8, 9a, 11a, 12
11		137.1 (s)	
11a	4.33 (d 12.0)	61.5 (t)	C10, 11, 12
	4.36 (d 12.0)		
12	5.91 (s)	138.2 (d)	C10, 11, 11a, 13a, 14
13		135.2 (s)	
13a	2.03 (s)	20.09 (q)	C12, 13
14	6.03 (s)	135.6 (d)	C12, 13a, 15, 15a, 16
15		139.1 (s)	
15a	2.07 (s)	20.06 (q)	C14, 15, 16
16	6.46 (s)	129.6 (d)	C14, 15, 15a, 17, 18, 22
17		145.1 (s)	
18,22	7.42 (d 8.8)	130.3 (d)	C16, 18, 20, 22
19,21	8.19 (d 8.8)	124.3 (d)	C17, 19, 20, 21
20		146.7 (d)	

#Compound **7**.

Supplementary Table 11. ^{1}H and ^{13}C NMR data for 2-pyrone-4-desmethyl-7-dehydro-7-deoxyneoaureothin[#].

Position	^{1}H (J_{Hz})	^{13}C (mult)	HMBC correlations (H to C)
1			
2		166.0 (s)	
3		99.0 (s)	
3a	1.80 (s)	8.3 (q)	C2, 3, 4
4		165.8 (s)	
5		108.3 (s)	
5a	1.89 (s)	9.1 (q)	C4, 5, 6
6		152.7 (s)	
7	6.19 (d 15.3)	114.5 (d)	C6, 8, 9
8	7.02 (d 15.3)	140.0 (d)	C6, 7, 9a, 10
9		133.2 (s)	
9a	1.90 (s)	13.7 (q)	C8, 9, 10
10	6.14 (s)	141.4 (d)	C8, 9, 9a, 11, 11a, 12
11		134.2 (s)	
11a	1.92 (s)	18.9 (s)	C10, 11, 12
12	5.87 (s)	137.2 (d)	C10, 13a, 14
13		135.7 (s)	
13a	1.92 (s)	19.2 (q)	C13
14	5.89 (s)	134.6 (d)	C12, 13a, 16
15		139.4 (s)	
15a	1.94 (s)	19.1 (q)	C14, 15, 16
16	6.33 (s)	127.9 (d)	C14,15a, 17, 18, 22
17		144.7 (s)	
18,22	7.30 (d 8.5)	129.3 (d)	C16, 18, 20, 22
19,21	8.03 (d 8.5)	123.3 (d)	C17, 19, 20, 21
20		145.5 (d)	

[#]Compound **8**.

Supplementary Table 12. ^{1}H and ^{13}C NMR data for 2-pyrone-7-dehydro-7-deoxyneoaureothin[#].

Position	^{1}H (J_{Hz})	^{13}C (mult)	HMBC correlations (H to C)
1			
2		165.1 (s)	
3		110.0 (s)	
3a	2.03 (s)	9.7 (q)	C2, 3, 4
4		168.3 (s)	
4a	3.79 (s)	61.0 (q)	C4
5		110.9 (s)	
5a	2.05 (s)	10.4 (q)	C4, 5, 6
6		153.4 (s)	
7	6.28 (d 15.4)	114.7 (d)	C6, 8, 9
8	7.21 (d 15.4)	140.4 (d)	C6, 9, 9a, 10
9		133.4 (s)	
9a	2.04 (s)	14.1 (q)	C8, 9, 10
10	6.30 (s)	142.0 (d)	C8, 9, 9a, 11a, 12
11		134.5 (s)	
11a	2.062 (s)	19.5 (s)	C10, 11, 12
12	6.02 (s)	137.6 (d)	C10, 13a, 14
13		134.5 (s)	
13a	2.060 (s)	19.3 (q)	C13
14	6.03 (s)	135.0 (d)	C12, 13a, 16
15		139.6 (s)	
15a	2.09 (s)	19.6 (q)	C14, 15, 16
16	6.47 (s)	128.2 (d)	C14,15a, 17, 18, 22
17		144.8 (s)	
18,22	7.42 (d 8.8)	129.5 (d)	C16, 18, 20, 22
19,21	8.18 (d 8.8)	123.5 (d)	C17, 19, 20, 21
20		145.8 (d)	

[#]Compound **9.**

Supplementary Table 13. ^{1}H and ^{13}C NMR data for 7-dehydro-7-deoxyneoaureothin#.

Position	^{1}H (J_{Hz})	^{13}C (mult)	HMBC correlations (H to C)
1			
2		161.7 (s)	
2a	4.06 (s)	55.4 (q)	C2
3		99.6 (s)	
3a	1.87 (s)	7.0 (q)	C2, 3, 4
4		181.0 (s)	
5		118.5 (s)	
5a	2.061 (s)	9.7 (q)	C4, 5, 6
6		152.6 (s)	
7	6.44 (d 15.5)	116.2 (d)	C6, 8, 9
8	6.95 (d 15.5)	139.3 (d)	C6, 9a, 10
9		133.3 (s)	
9a	2.074 (s)	14.1 (q)	C8, 9, 10
10	6.25 (s)	141.7 (d)	C8, 9a, 11a, 12
11		134.1 (s)	
11a	2.08 (s)	19.3 (s)	C10, 11, 12
12	6.041 (s)	137.6 (d)	C10, 13a, 14
13		135.7 (s)	
13a	2.066 (s)	19.6 (q)	C13
14	6.035 (s)	135.1 (d)	C12, 13a, 16
15		139.4 (s)	
15a	2.09 (s)	19.5 (q)	C14, 15
16	6.47 (s)	128.3 (d)	C14,15a, 17, 18, 22
17		144.7 (s)	
18,22	7.42 (d 9.0)	129.5 (d)	C16, 18, 20, 22
19,21	8.18 (d 9.0)	123.5 (d)	C17, 19, 20, 21
20		145.9 (d)	

#Compound **10**.

Supplementary References

1. Buchholz, T.J. et al. Structural basis for binding specificity between subclasses of modular polyketide synthase docking domains. *ACS Chem. Biol.* **4**, 41-52 (2009).
2. Dorival, J. et al. Characterization of Intersubunit Communication in the Virginiamycin trans-Acyl Transferase Polyketide Synthase. *J. Am. Chem. Soc.* **138**, 4155-4167 (2016).
3. Broadhurst, R.W., Nietlispach, D., Wheatcroft, M.P., Leadlay, P.F. & Weissman, K.J. The structure of docking domains in modular polyketide synthases. *Chem. Biol.* **10**, 723-731 (2003).
4. Whicher, J.R. et al. Cyanobacterial polyketide synthase docking domains: a tool for engineering natural product biosynthesis. *Chem. Biol.* **20**, 1340-1351 (2013).
5. Thattai, M., Burak, Y. & Shraiman, B.I. The origins of specificity in polyketide synthase protein interactions. *PLoS Comput. Biol.* **3**, 1827-1835 (2007).
6. Sievers, F. et al. Fast, scalable generation of high-quality protein multiple sequence alignments using Clustal Omega. *Mol. Syst. Biol.* **7**, 539 (2011).
7. Waterhouse, A.M., Procter, J.B., Martin, D.M., Clamp, M. & Barton, G.J. Jalview Version 2--a multiple sequence alignment editor and analysis workbench. *Bioinformatics* **25**, 1189-1191 (2009).
8. Caffrey, P., Bevitt, D.J., Staunton, J. & Leadlay, P.F. Identification of DEBS 1, DEBS 2 and DEBS 3, the multienzyme polypeptides of the erythromycin-producing polyketide synthase from Saccharopolyspora erythraea. *FEBS Lett.* **304**, 225-228 (1992).
9. Xue, Y., Zhao, L., Liu, H.W. & Sherman, D.H. A gene cluster for macrolide antibiotic biosynthesis in *Streptomyces venezuelae*: architecture of metabolic diversity. *Proc. Natl. Acad. Sci. U. S. A.* **95**, 12111-12116 (1998).
10. Caffrey, P., Lynch, S., Flood, E., Finnan, S. & Oliynyk, M. Amphotericin biosynthesis in Streptomyces nodosus: deductions from analysis of polyketide synthase and late genes. *Chem. Biol.* **8**, 713-723 (2001).
11. Yu, T.W. et al. The biosynthetic gene cluster of the maytansinoid antitumor agent ansamitocin from *Actinosynnema pretiosum*. *Proc. Natl. Acad. Sci. U. S. A.* **99**, 7968-7973 (2002).
12. Brautaset, T. et al. Biosynthesis of the polyene antifungal antibiotic nystatin in *Streptomyces noursei* ATCC 11455: analysis of the gene cluster and deduction of the biosynthetic pathway. *Chem. Biol.* **7**, 395-403 (2000).
13. Oliynyk, M. et al. Analysis of the biosynthetic gene cluster for the polyether antibiotic monensin in Streptomyces cinnamonensis and evidence for the role of monB and monC genes in oxidative cyclization. *Mol. Microbiol.* **49**, 1179-1190 (2003).
14. Waldron, C. et al. Cloning and analysis of the spinosad biosynthetic gene cluster of Saccharopolyspora spinosa. *Chem. Biol.* **8**, 487-499 (2001).
15. Castonguay, R. et al. Stereospecificity of ketoreductase domains 1 and 2 of the tylactone modular polyketide synthase. *J. Am. Chem. Soc.* **130**, 11598-11599 (2008).
16. Aparicio, J.F., Colina, A.J., Ceballos, E. & Martin, J.F. The biosynthetic gene cluster for the 26-membered ring polyene macrolide pimaricin. A new polyketide synthase organization encoded by two subclusters separated by functionalization genes. *J. Biol. Chem.* **274**, 10133-10139 (1999).
17. August, P.R. et al. Biosynthesis of the ansamycin antibiotic rifamycin: deductions from the molecular analysis of the rif biosynthetic gene cluster of *Amycolatopsis mediterranei* S699. *Chem. Biol.* **5**, 69-79 (1998).
18. Yuzawa, S. et al. Comprehensive in vitro analysis of acyltransferase domain exchanges in modular polyketide synthases and its application for short-chain ketone production. *ACS Synth. Biol.* **6**, 139-147 (2017).
19. Kelley, L.A., Mezulis, S., Yates, C.M., Wass, M.N. & Sternberg, M.J. The Phyre2 web portal for protein modeling, prediction and analysis. *Nat. Protoc.* **10**, 845-858 (2015).

20. Maier, T., Leibundgut, M. & Ban, N. The crystal structure of a mammalian fatty acid synthase. *Science* **321**, 1315-1322 (2008).
21. Ikeda, H., Nonomiya, T., Usami, M., Ohta, T. & Omura, S. Organization of the biosynthetic gene cluster for the polyketide anthelmintic macrolide avermectin in *Streptomyces avermitilis*. *Proc. Natl. Acad. Sci. U. S. A.* **96**, 9509-9514 (1999).
22. Omura, S. et al. Genome sequence of an industrial microorganism *Streptomyces avermitilis*: deducing the ability of producing secondary metabolites. *Proc. Natl. Acad. Sci. U. S. A.* **98**, 12215-12220 (2001).
23. Shah, S. et al. Cloning, characterization and heterologous expression of a polyketide synthase and P-450 oxidase involved in the biosynthesis of the antibiotic oleandomycin. *J. Antibiot. (Tokyo)* **53**, 502-508 (2000).
24. Kakavas, S.J., Katz, L. & Stassi, D. Identification and characterization of the niddamycin polyketide synthase genes from Streptomyces caelestis. *J. Bacteriol.* **179**, 7515-7522 (1997).
25. Motamedi, H., Cai, S.J., Shafiee, A. & Elliston, K.O. Structural organization of a multifunctional polyketide synthase involved in the biosynthesis of the macrolide immunosuppressant FK506. *Eur. J. Biochem.* **244**, 74-80 (1997).
26. Schwecke, T. et al. The biosynthetic gene cluster for the polyketide immunosuppressant rapamycin. *Proc. Natl. Acad. Sci. U. S. A.* **92**, 7839-7843 (1995).
27. Sun, Y. et al. A complete gene cluster from *Streptomyces nanchangensis* NS3226 encoding biosynthesis of the polyether ionophore nanchangmycin. *Chem. Biol.* **10**, 431-441 (2003).
28. Carmody, M. et al. Biosynthesis of amphotericin derivatives lacking exocyclic carboxyl groups. *J. Biol. Chem.* **280**, 34420-34426 (2005).
29. Chen, S. et al. Organizational and mutational analysis of a complete FR-008/candicidin gene cluster encoding a structurally related polyene complex. *Chem. Biol.* **10**, 1065-1076 (2003).
30. Anzai, Y. et al. Organization of the biosynthetic gene cluster for the polyketide macrolide mycinamicin in *Micromonospora griseorubida*. *FEMS Microbiol. Lett.* **218**, 135-141 (2003).
31. Volchegursky, Y., Hu, Z., Katz, L. & McDaniel, R. Biosynthesis of the anti-parasitic agent megalomicin: transformation of erythromycin to megalomicin in *Saccharopolyspora erythraea*. *Mol. Microbiol.* **37**, 752-762 (2000).
32. Liu, C.X. et al. A new spectinabilin derivative with cytotoxic activity from ant-derived *Streptomyces* sp. 1H-GS5. *J. Asian Nat. Prod. Res.* **19**, 924-929 (2017).
